# Accuracy of health administrative data to identify cases of reportable travel or migration-related infectious diseases in Ontario, Canada

**DOI:** 10.1371/journal.pone.0207030

**Published:** 2018-11-07

**Authors:** Rachel D. Savage, Laura C. Rosella, Natasha S. Crowcroft, Maureen Horn, Kamran Khan, Monali Varia

**Affiliations:** 1 Dalla Lana School of Public Health, University of Toronto, Toronto, Ontario, Canada; 2 ICES, Toronto, Ontario, Canada; 3 Public Health Ontario, Toronto, Ontario, Canada; 4 Department of Laboratory Medicine and Pathobiology, University of Toronto, Toronto, Ontario, Canada; 5 Peel Public Health, Mississauga, Ontario, Canada; 6 Li Ka Shing Knowledge Institute, St. Michael’s Hospital, Toronto, Ontario, Canada; 7 Department of Medicine, Division of Infectious Diseases, University of Toronto, Toronto, Ontario, Canada; Uniformed Services University of the Health Sciences, UNITED STATES

## Abstract

An ongoing challenge of estimating the burden of infectious diseases known to disproportionately affect migrants (e.g. malaria, enteric fever) is that many health information systems, including reportable disease surveillance systems, do not systematically collect data on migrant status and related factors. We explored whether health administrative data linked to immigration records offered a viable alternative for accurately identifying cases of hepatitis A, malaria and enteric fever in Ontario, Canada. Using linked health care databases generated by Ontario’s universal health care program, we constructed a cohort of medically-attended individuals with presumed hepatitis A, malaria or enteric fever in Peel region using diagnostic codes. Immigrant status was ascertained using linked immigration data. The sensitivity and positive predictive value (PPV) of diagnostic codes was evaluated through probabilistic linkage of the cohort to Ontario’s reportable disease surveillance system (iPHIS) as the reference standard. Linkage was successful in 90.0% (289/321) of iPHIS cases. While sensitivity was high for hepatitis A and enteric fever (85.8% and 83.7%) and moderate for malaria (69.0%), PPV was poor for all diseases (0.3–41.3%). The accuracy of diagnostic codes did not vary by immigrant status. A dated coding system for outpatient physician claims and exclusion of new immigrants not yet eligible for health care were key challenges to using health administrative data to identify cases. Despite this, we show that linkages of health administrative and immigration records with reportable disease surveillance data are feasible and have the potential to bridge important gaps in estimating burden using either data source independently.

## Introduction

As populations become more connected and diverse through travel and immigration, there is a growing interest in understanding the health needs of migrant populations to protect their health and that of the broader public [1]. Migrant travellers who travel to their birth countries to visit friends and relatives (VFR travellers) are an important and growing risk group for travel-related infectious diseases [2]. In ethnoculturally diverse regions in Canada and around the world, VFR travellers account for the majority of hepatitis A, malaria, and enteric fever cases reported to public health [3]. These diseases are preventable through safe and effective vaccines or chemoprophylaxis [4–6]; however, travel health services are not reimbursed in many countries with universal health insurance plans including Canada, although some countries (e.g. England and Australia) provide coverage for select services [7–9]. Given emerging trends in globalization and climate change that are increasing opportunities for exposure [10], it is prudent to measure the burden of travel and migration-related infectious diseases to evaluate current policies and develop effective public health interventions.

Reportable disease surveillance systems are the mainstay for burden estimation as many travel-related infectious diseases are notifiable by law [11]. Despite this, an important limitation is they usually lack information on country of birth, region of origin, or immigrant status, which are needed to appropriately target preventive measures. Accordingly, the European Centre for Disease Prevention and Control has called for better data to improve understanding of risk groups of imported malaria in Europe [12]. Another important challenge with public health surveillance data is poor sensitivity as cases may not seek health care or be reported to public health (which varies by disease [13, 14]), or they may seek health care or die from their infection while travelling [15, 16]. These limitations may explain the lack of studies estimating burden of travel or migration-related infectious diseases, despite the value of this information to decision-makers [17].

Routinely collected health administrative data (e.g. data generated during health care delivery such as records of hospitalization, outpatient physician visits, emergency department visits, etc.) offer an efficient and inexpensive alternative to public health surveillance data for burden estimation of medically-attended travel or migration-related diseases. They also provide an opportunity to overcome existing data limitations through population-based linkages with immigration data [18] and to address important gaps if diseases are removed from notifiable disease lists (as in Ontario recently for malaria). Because the validity of these data for identifying travel or migration-related infectious diseases is not well-understood, we sought to examine if health administrative data could be used to accurately identify cases of hepatitis A, malaria and enteric fever compared with reportable disease surveillance data as the reference standard.

## Materials and methods

### Study setting

Peel region is one of Canada’s largest and ethnically-diverse municipalities with approximately 1.4 million residents, half of whom are foreign-born [19]. In this study, we used reportable disease data collected by Peel Public Health (PPH) and health administrative data that were collected as part of Ontario’s government-funded, universal health care.

### Reference standard

Reportable disease data in Ontario are captured in the integrated public health information system (iPHIS), a web-based information system used for reporting of notifiable infectious disease cases to provincial authorities. We extracted data on laboratory-confirmed cases of hepatitis A, malaria, and enteric fever (i.e. typhoid and paratyphoid fever) reported between January 1, 2012 and December 31, 2014 with PPH listed as the responsible health unit for case management.

### Study cohort

We constructed a cohort of medically-attended individuals with presumed hepatitis A, malaria or enteric fever in Peel region through deterministic linkage of the Canadian Institutes for Health Information’s hospital discharge abstract (DAD) and same-day surgeries databases (SDS), National Ambulatory Care Reporting System (NACRS), and Ontario Health Insurance Plan (OHIP) physician claims. These databases include all persons covered under Ontario’s universal health insurance plan (new immigrants with the exception of refugees have a three month wait period before they are eligible for universal health care [20]), and are held at ICES [21]. Diagnoses are coded using the International Classification of Diseases, 10th revision Canada (ICD-10-CA) for all databases aside from OHIP that uses a similar, but not identical, version of ICD-8 that identifies broad disease classifications.

We identified a set of sensitive diagnostic codes to select individuals (S1 File) that were felt to reflect a confirmed diagnosis, symptoms, or interpretation of a diagnostic test for the diseases of interest based on consultation with an infectious disease physician and the literature [22, 23]. For the primary analysis, we restricted the cohort to the most presumed specific codes (i.e. ICD-10 codes B15, B50-54, A01 or OHIP codes 070, 062, 002). There are OHIP diagnostic codes available for viral hepatitis (070) and typhoid and paratyphoid fevers (002) but none for malaria. As a result, we selected code 062 (mosquito-borne viral encephalitis) because of its reference to mosquito-based transmission. We also performed a post-hoc sensitivity analysis using code 136 (other infectious or parasitic diseases), which is referenced in the Resource Manual for Physicians for malaria [24].

Any records with a valid ICES key number (IKN), a unique identifier based on their health card, that met the diagnostic selection criteria between November 20, 2011 and February 11, 2015 were included. This time period provided a six-week buffer relative to the case report date, recognizing that individuals seek health care prior to being reported to public health, and may require follow-up visits.

**Fig 1:**
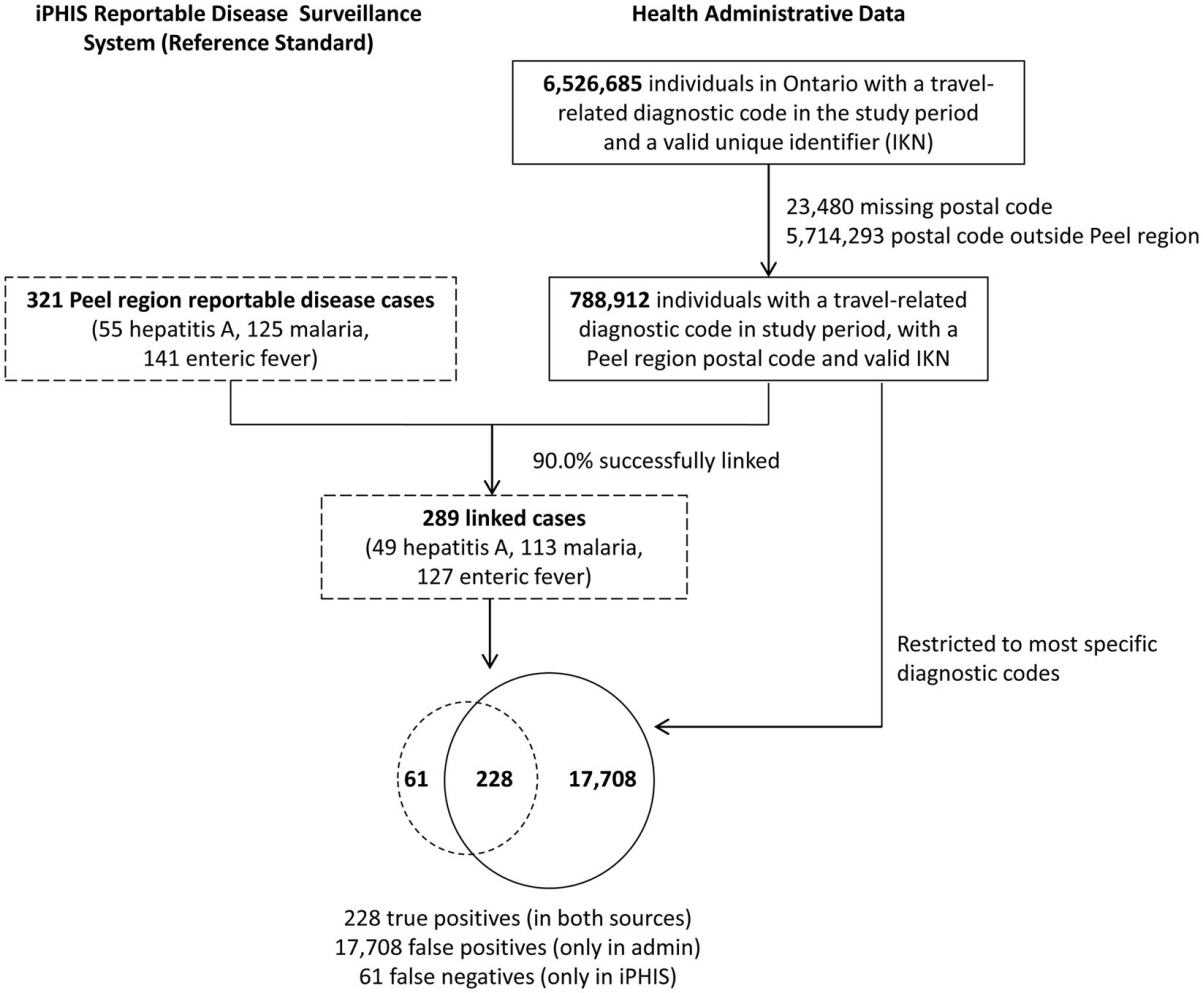
Study flow chart.

Records meeting our inclusion criteria were linked deterministically across databases. These datasets were linked using unique encoded identifiers and analyzed at ICES. Records were further restricted to Peel region residents using the individual’s postal code in the Registered Persons Database (RPDB). We excluded individuals with a missing postal code (0.4%) (Fig 1).

Individuals with health care encounters 3 to 24 months prior to cohort entry were flagged to explore potential misclassification (e.g. illness might have been chronic and non-travel related). Remaining records were linked to the Ontario portion of the Immigration, Refugee and Citizenship Canada’s permanent resident (IRCC-PR) database to ascertain immigrant status. IRCC-PR data capture landed immigrants to Ontario from 1985 to 2012 who obtained a valid Ontario health card number [18]. Immigrant status was of interest given that VFR travellers experience disproportionate travel-related morbidity [25–27] and may also seek care differently or have differential access to diagnosis versus long-term residents [28]. Any individual present in the IRCC-PR was classified as an immigrant, recognizing the important limitation that individuals who immigrated pre-1985 and post-2012 would not be classified. Additionally, new immigrants who currently reside in Ontario but who landed in another province were not classified. The dataset creation plan for the study cohort is in S2 File.

### Linkage

To assess the validity of diagnostic codes in the health administrative data relative to confirmed cases reported to public health, iPHIS data were probabilistically linked via unique identifiers (first and last name, date of birth, sex, address) to the RPDB to obtain the IKN. Cases were then deterministically linked to the study cohort.

### Analysis

Un-linkable cases were excluded (n = 32) but compared on demographic variables to linked cases. In the linked cohort, individuals with ≥1 health encounter during the study period meeting the diagnostic criteria in the health administrative data were identified as test positives. Classifications were made at the individual-level, not at the encounter-level, and prioritized according to travel-related diagnoses. For example, if an individual had two health encounters in OHIP during the study period where the first visit was coded 787 and the second 070, the individual was classified as a test positive for hepatitis A given their second encounter. Individuals who were test positives and who were also confirmed cases in the reference standard (iPHIS) were classified as true positives; individuals that were test positives but not in iPHIS were classified as false positives. Cases in iPHIS that were test negatives (i.e. not present in the health administrative data with a relevant diagnostic code) were classified as false negatives. We also created disease-specific decision rules to exclude health encounters that were unlikely to be related to the disease episode by graphing distribution curves of time between health care encounters and iPHIS dates for all disease codes to identify outlying values.

We calculated two measures of validity relative to the reference standard—sensitivity and positive predictive value (PPV)—as iPHIS does not allow for the identification of true negatives. Sensitivity was calculated as [true positives/(true positives + false negatives)]x100. Positive predictive value was calculated as [true positives/(true positives + false positives)]x100. 95% confidence intervals were calculated using the standard formula for the standard error of a proportion [29]. Validity estimates were stratified by administrative data source and immigrant status.

### Post-hoc sensitivity and additional analyses

We explored whether certain exclusions could improve the specificity of the health administrative data and OHIP, in particular. First, we excluded records with ICD-10 coded health encounters (DAD, NACRS) where the diagnosis prefix was indicated as questionable (DXPREF = Q) to explore the contribution of uncertain diagnoses [30]. Second, we excluded records with a generic immunization fee code (G538) billed 0 to 60 days prior to the individuals’ outpatient/primary care physician diagnostic visit (OHIP) or if the billed fee code G538 had a corresponding diagnostic code of 070 (hepatitis A), 136 (malaria) or 002 (typhoid/paratyphoid fever). This was designed to exclude individuals whose health encounter was a consequence of an immunization, or for whom the diagnostic code(s) may have been used incorrectly to capture an immunization or disease prevention consultation (e.g. chemoprophylaxis). Third, we excluded individuals that had a physician visit with an 070 diagnosis (viral hepatitis) during the study and lookback period to exclude individuals that may have chronic hepatitis.

Knowing that iPHIS is an imperfect standard, we also performed a two-source capture-recapture (CRC) analysis to estimate reporting completeness (S3 File). Analyses were performed in SAS version 9.4 (S4 File for analytic code). Ethics approval was granted by the University of Toronto’s Research Ethics Board (Protocol Reference: 31366).

## Results

Over the three year period, 55 confirmed cases of hepatitis A, 125 of malaria, and 141 of enteric fever (N = 321) were reported to PPH (Fig 1). In the study cohort, there were 788,912 unique individuals with presumed travel or migration-related infections with a corresponding 2,539,975 health care encounters (Fig 1). The majority of these encounters were outpatient/primary care physician visits (2,499,947 or 98.4%), followed by emergency department visits (33,314 or 1.3%), hospitalizations (5,279 or 0.2%) and then same-day surgeries (1,417 or 0.1%).

Probabilistic linkage of iPHIS cases to the RPDB was successful for 90.0% (289/321) of cases. Un-linked cases were more likely to be a recent immigrant or visitor to Ontario compared to linked cases (53.3% vs. 8.7%, P < 0.001), likely because they did not have an Ontario health card, but otherwise were similar in terms of sex, age, disease distribution and foreign-born status.

Incidence rates of hepatitis A, malaria and enteric fever were substantially higher in the health administrative data compared with the reference standard (Table 1).

**Table 1:** Number of cases and disease incidence rates (per 100,000) as determined by the reference standard (iPHIS) compared to health administrative data, November 20, 2011 to February 11, 2015.

Disease	iPHIS^a^		Health Administrative Data			
			Specific Codes		Sensitive Codes	
	n	Rate	n	Rate	n	Rate
Hepatitis A	55	1.36	15,992	350.90	615,903	13,514.34
Malaria	125	3.10	189	4.15	666,007	14,613.73
Enteric Fever	141	3.49	1,755	38.51	542,101	11,894.95

^a^ iPHIS, integrated public health information system (the reference standard).

Sensitivity varied by disease (Table 2) and was lowest for malaria given that there were zero true positives with 062-coded health encounters in OHIP. Over 80% of hepatitis A cases reported to public health were present in health administrative data with diagnostic codes 070 (OHIP) or B15 (ICD-10); however, only 0.3% of those with these codes were a laboratory-confirmed case reported to public health. PPV was similarly low for enteric fever but improved for malaria (PPV = 43.0%). In the post-hoc sensitivity analysis replacing OHIP code 062 with 136, the number of true and false positives rose to 89 and 7,556 respectively, with a decrease in false negatives to 24 [sensitivity = 78.8% (95% CI 71.2–86.3); PPV = 1.2% (95% CI 0.9–1.4)].

**Table 2:** Accuracy of diagnostic codes for hepatitis A, malaria and enteric fever used in health administrative data, overall and by data source, with Ontario’s reportable disease registry (iPHIS) as the reference standard.

Data Source	Disease	Health Admin.	iPHIS case		Sensitivity 95% CI^b^	PPV^a^ 95% CI^b^
			+	-		
All combined	Hepatitis A	+	41	15,951	83.7 (73.3–94.0)	0.3 (0.2–0.3)
		-	8	--		
	Malaria	+	78	111	69.0 (60.5–77.6)	41.3 (34.3–48.3)
		-	35	--		
	Enteric Fever	+	109	1,646	85.8 (79.8–91.9)	6.2 (5.1–7.3)
		-	18	--		
Hospitalizations (ICD-10)	Hepatitis A	+	18	10	36.7 (23.2–50.2)	64.3 (46.5–82.0)
		-	31	--		
	Malaria	+	58	16	51.3 (42.1–60.5)	78.4 (69.0–87.8)
		-	55	--		
	Enteric Fever	+	78	29	61.4 (53.0–69.9)	72.9 (64.5–81.3)
		-	49	--		
Emergency Department Visits (ICD-10)	Hepatitis A	+	11	16	22.4 (10.8–34.1)	40.7 (22.2–59.3)
		-	38	--		
	Malaria	+	74	49	65.5 (56.7–74.3)	60.2 (51.5–68.8)
		-	39	--		
	Enteric Fever	+	60	66	47.2 (38.6–55.9)	47.6 (38.9–56.3)
		-	67	--		
Outpatient physician visits (OHIP)	Hepatitis A	+	39	15,940	79.6 (68.3–90.9)	0.2 (0.2–0.3)
		-	10	--		
	Malaria	+	0	56	0.0 (0.0–0.0)	0.0 (0.0–0.0)
		-	113	--		
	Enteric Fever	+	99	1,580	78.0 (70.7–85.2)	5.9 (4.8–7.0)
		-	28	--		

^a^ PPV, positive predictive value.

^b^ CI, confidence interval.

--no data.

When stratified by health administrative database (Table 2), diagnostic codes in outpatient/primary care physician visit data (OHIP) were more sensitive than hospitalization (DAD) and emergency department visit (NACRS) data for both hepatitis A and enteric fever. For malaria, sensitivity was highest for emergency department visit data. Hospitalization data provided the highest PPV for all diseases.

Sensitivity estimates were similar in immigrants compared to long-standing residents across all diseases (87% vs. 81% hepatitis A; 69% vs. 68% malaria; 84% vs. 89% enteric fever). PPV was similar across groups for hepatitis A (0.3% vs. 0.3%) but higher amongst immigrants for malaria (57.1% vs. 26.5%) and enteric fever (7.8% vs. 4.9%).

We compared true and false positives (Table 3) and found that true positives were more likely to be younger (with the exception of malaria), to not have had similar health encounters in the lookback period, and to be an immigrant. True positives were also more likely to be hospitalized or visit the ED than false positives and to have a higher number of health encounters but spaced over a shorter duration. In total, 53 false positives had a specific diagnostic ICD-10 code assigned to a hospitalization, and likely represent true, unreported disease cases.

**Table 3:** Characteristics of true (TP) and false positive (FP) individuals, by disease.

Characteristics		Hepatitis A			Malaria			Enteric Fever		
		TP (N = 41) n (%)	FP (N = 15,951) n (%)	P-value	TP (N = 78) n (%)	FP (N = 111) n (%)	P-value	TP (N = 109) n (%)	FP (N = 1,646) n (%)	P-value
Sex	Female	20 (48.8)	7,356 (46.1)	0.733	25 (32.1)	59 (53.2)	0.004	47 (43.1)	895 (54.4)	0.022
Age (at cohort entry)	median (IQR^a^)	18 (12–25)	44 (33–55)	< .001	43 (28–55)	29 (12–54)	0.012	27 (9–40)	44 (26–59)	< .001
Immigrant	Yes	20 (48.8)	7,699 (48.3)	0.948	52 (66.7)	39 (35.1)	< .001	61 (56.0)	723 (43.9)	0.014
Codes in lookback	Yes	9 (22.0)	7,361 (46.1)	0.002	17 (21.8)	37 (33.3)	0.084	30 (27.5)	606 (36.8)	0.051
Source^b^	DAD^c^	18 (43.9)	117 (0.7)	< .001	58 (74.4)	16 (14.4)	< .001	87 (79.8)	64 (3.9)	< .001
	NACRS^d^	12 (29.3)	379 (2.4)	< .001	74 (94.9)	49 (44.1)	< .001	76 (69.7)	146 (8.9)	< .001
	OHIP^e^	40 (97.6)	15,950 (100.0)	< .001	70 (89.7)	100 (90.1)	0.938	106 (97.2)	1,641 (99.7)	< .001
Number of health encounters/person	median (IQR)	6 (3–9)	3 (2–6)	< .001	5 (3–7)	3 (2–6)	0.002	6 (4–10)	2 (1–4)	< .001
Days from first to last health encounter	median (IQR)	29 (6–246)	379 (21–772)	0.003	18 (3–87)	273 (5–694)	< .001	47 (13–321)	248 (0–672)	0.158

^a^ IQR, interquartile range.

^b^ not mutually exclusive.

^c^ DAD, hospital discharge abstract database for hospitalizations.

^d^ NACRS, National Ambulatory Care Reporting System for emergency department visits.

^e^ OHIP, Ontario’s universal health insurance plan claims database for reimbursement of outpatient physician services.

A description of cases reported to public health that were not present in the health administrative data is provided in Table 4. Of the 61 false negatives, 18 had no health encounters matching any of the specified diagnostic codes. The remaining 43 cases were present in the health administrative data but did not have a specific diagnostic code. Amongst the 43 cases, 42 (97.7%) accessed primary care, while <6 visited the emergency department or were hospitalised. Overall, false negatives were predominantly younger and male. Almost one-quarter (21.3%) were not eligible for OHIP at the time of their illness. While all cases had PPH indicated as their responsible health unit, only 63.9% had a Peel region postal code assigned in the health administrative data based on their most recent contact with the health system.

**Table 4:** Characteristics of false negatives.

Characteristic	Category	Overall (N = 61)	
		n	%
Sex	Female	18	29.5
Age in years	median (IQR^a^)	38 (18–49)	
Peel region resident	Yes	39	63.9
Immigrant	Yes	38	62.3
World Region	Asia and Pacific	21	55.3
	Other	17	44.7
Country of birth	India	23	37.7
	Other or Unknown	38	85.3
Diagnostic codes in lookback period	Yes	9	14.8
OHIP^b^ eligibility at illness	Yes	48	78.7
OHIP^b^ eligibility at 1 year	Yes	43	70.5
OHIP^b^ eligibility at 2 years	Yes	42	68.9
OHIP^b^ eligibility at 3 years	Yes	39	63.9
Hospitalized	Yes	10	16.4
Travel Associated^c^	Yes	47	77.1

^a^ IQR, interquartile range.

^b^ OHIP, Ontario’s universal health insurance plan claims database for reimbursement of outpatient physician services.

^c^ includes recent immigrant or visitor.

Broadening the diagnostic codes used to classify individuals as having hepatitis A, malaria, or enteric fever improved sensitivity by ~10–15%; however, PPV decreased near zero for every disease (Table 5). All exclusions made in post-hoc sensitivity analyses led to either no change or a slight decrease in sensitivity with only minor improvements in PPV (S5 File).

**Table 5:** Test characteristics of sensitive (low specificity) health administrative diagnostic codes of hepatitis A, malaria and enteric fever diseases with the reportable disease registry iPHIS as the reference standard.

Disease	Health Admin.	iPHIS case		Sensitivity 95% CI^b^	PPV^a^ 95% CI^b^
		+	-		
Hepatitis A	test +	40–50	615,855	98.0% (94.0–100.0)	0.0% (0.0–0.0)
	test -	≤5	--		
Malaria	test +	95	665,912	84.1% (77.3–90.8)	0.0% (0.0–0.0)
	test -	18	--		
Enteric Fever	test +	121	541,980	95.3% (91.6–99.0)	0.0% (0.0–0.0)
	test -	6	--		

^a^ PPV, positive predictive value.

^b^ CI, confidence interval.

test + refers to having a health encounter with a relevant diagnostic code in a health administrative data source; test − refers to the absence of a health encounter with a relevant diagnostic code.

## Discussion

We found that diagnostic codes in Ontario’s health care databases had good sensitivity (69–86%) to identify true cases of hepatitis A, malaria and enteric fever reported to public health. An additional 10–15% of cases were present in health administrative data but with less specific codes. Despite this, only a small proportion of individuals with diagnostic codes for these diseases were true cases identified in reportable disease surveillance data (PPV range: 0–41%).

While the utility and validity of health administrative data for chronic disease surveillance has been extensively studied worldwide, the use of these data for infectious diseases is still in its infancy [23, 31]. As the potential of these data expand, growing to encompass electronic health records and population-level integrated laboratory databases, characterization of the limitations and strengths of health administrative data for identifying infectious disease cases is needed. In our study, we identify several challenges in the context of travel and migration-related infectious diseases. The first is that recent immigrants may be underrepresented in burden studies reliant solely on health administrative data if universal health coverage policies exclude these new residents. Secondly, the predictive accuracy of these data is dependent on precise and up-to-date diagnostic coding systems that have substantial diagnostic specificity [32]. In Ontario, the lack of disease-specific codes in primary care largely drove the poor PPV we observed (e.g. codes did not distinguish between hepatitis types, nor was there a dedicated code for malaria). As primary care is often the first and only point of contact with the health care system for infected individuals, improving coding in this setting is particularly important. Updating the antiquated coding system of physician billings to the latest coding system (i.e. ICD-10) could vastly improve both the sensitivity and PPV of health administrative data for travel and migration-related diseases and bring alignment with other health care data. In the meantime, quality improvement initiatives may help standardize how current codes are being used by physicians.

Beyond coding systems, there are challenges inherent in assigning diagnoses for infections. For example, disease diagnoses are rarely confirmed by laboratory testing at the time the physician sees the individual. This uncertainty can lead to false positives if disease codes include those with suspected, not confirmed, infections [23], and also false negatives if generic codes are assigned to indicate no diagnosis (e.g. we found that half of the malaria false negatives in our study were assigned code 999 (no diagnosis) in OHIP). Linking primary care databases with electronic laboratory data could address this challenge. Lastly and perhaps the most difficult challenge to address are physician-related barriers to accurate coding. Through interviews with professional coding specialists, Tang et al. recently identified incomplete and nonspecific documentation, errors and discrepancies within patient charts, disconnects between physician and coder terminologies, and lack of communication between physicians and coders as key barriers to accurate coding [33]. Study authors acknowledge that these barriers have structural and systemic causes that often extend beyond the control of physicians and as such, are complex to address. Despite this, as the use of health administrative data for research, surveillance, and evaluation grows, and with it the need to evaluate health care to maximize efficiencies and health outcomes, improving the accuracy of these data should be prioritized so that they can reliably inform decision-making [33].

Despite these challenges, there are strengths of health administrative data for travel and migration-related infectious diseases that can be leveraged to further improve its accuracy and utility. First, health administrative data are linkable to reportable disease surveillance data and fill important gaps by providing objective measures of health care utilization and outcomes of travel or migration-related disease cases. Further linkage with immigration records can enable study on health care burden and cost of these infections broadly and also in migrants and be used for resource prioritization and setting. We also identified that hospitalization records, on their own, are likely useful for accurately identifying severe cases of hepatitis A, malaria and enteric fever given the relatively high PPV of diagnostic codes compared with ED and primary care data. Lastly, we identified a number of demographic and health encounter-related differences between true and false positives that may be exploited to improve the accuracy of health administrative data for infectious diseases. For example, Yu et al (2013) have shown that a classification algorithm that incorporates additional administrative information associated with a hospitalization can improve the accuracy of administrative data to detect individuals with community-acquired pneumonia [34]. Together, our findings suggest that health administrative data have the potential to add value to infectious disease surveillance, and merit further investigation of how their utility can be improved.

Although we took measures to be comprehensive in our selection of diagnostic codes, it is possible that alternative codes are being used by physicians, which may have underestimated the accuracy of health administrative data. In our study, 16% of laboratory-confirmed malaria cases reported to public health were not present in the health administrative data with any of the diagnostic codes we felt were relevant. In a subsequent study, we reviewed the complete clinical record of cases and found that a small proportion (<5%) of cases of malaria and enteric fever were additionally assigned OHIP codes 038 (septicemia) and 780 (convulsions, ataxia, vertigo, headache); however, as these codes were not detected in false negative cases, failure to include these would not have changed our sensitivity estimates (data not shown). We recommend any future studies of travel and migration-related infectious diseases using OHIP data conduct a chart review to validate case definitions first, where possible, to optimize accuracy.

A second limitation of our study is the use of postal codes to restrict our study cohort to Peel residents. Postal code is assigned based on the address reported on the individual’s health card but is supplemented with other ICES-held datasets that collect residence information at each health encounter [35]. Postal codes are estimated annually to select the most recent data as of July 1st as the reference date [35]. Therefore, movement in and out of Peel within one year may mean that individuals who lived in Peel at the time of their diagnosis but later moved in that same year may have been excluded (or vice-versa). During our study period, ~80,000 persons/year moved to Peel (<6% of total population) and ~65,000 moved out of Peel (<5%), and so it is not expected that misclassifications would appreciably change our findings.

Lastly, as iPHIS is an imperfect standard, some of the individuals we classified as false positives may have been true disease cases. To assess the impact of this potential misclassification, we used capture-recapture methods to estimate an additional 37 cases not captured in iPHIS or hospitalization records, for an iPHIS reporting completeness of 74% (95% CI 61–83%) for hepatitis A, 82% (95% CI 75–88%) for malaria, and 78% (95% CI 71–84%) for enteric fever (S2 File). These estimates are much higher than what was recently reported for pertussis in Ontario using a similar methodology [36], suggesting that the impact of this misclassification would likely be small. Indeed, if we moved these 37 cases from the false positive to true positive category, there would be <5% absolute improvement in sensitivity and <20% absolute improvement in PPV. Investigating false positives can identify if there are opportunities to improve hospital/physician reporting practices to public health.

## Conclusion

Our findings highlight challenges and strengths of using health administrative data to identify cases of travel or migration-related infectious disease, and caution against the use of these data on their own (i.e. without linkage to records confirming infection) to identify cases. Linking these data with reportable disease surveillance data and immigration records can, however, bridge important gaps in estimating burden using either data source independently. Linkages with electronic laboratory data, and initiatives aimed at improving the coding of health administrative data, particularly in primary care settings, offer potential to further improve the utility of these data for understanding the health needs of migrants.  

## Supporting information

S1 File Diagnostic codes used to identify presumed cases of hepatitis A, malaria and enteric fever in Ontario health administrative data. (PDF)

S2 File Dataset creation plan for health administrative data (study) cohort. (PDF)

S3 File Capture-recapture analysis to estimate degree of underreporting of hepatitis A, malaria and enteric fever in Ontario. (PDF)

S4 File SAS analytic code. (PDF)

S5 File Sensitivity analyses. (PDF)
