# Supplementary material for: Accuracy of health administrative data to identify cases of reportable travel or migration-related infectious diseases in Ontario, Canada
Source: PLoS One. 2018 Nov 7;13(11):e0207030. doi: 10.1371/journal.pone.0207030 (PMC6221317; doi:10.1371/journal.pone.0207030)
Supplement: S1 File — (PDF) [file pone.0207030.s001.pdf]

Supporting Information File 1: Diagnostic codes used to identify presumed cases of hepatitis A, malaria and enteric fever in Ontario health administrative data

An individual was selected into the health administrative cohort if they had a health encounter with at least one of the sensitive diagnostic codes during the study period; the cohort was restricted to specific diagnostic codes for the primary analysis.

**Disease**	**Diagnostic Code Type**	**Sensitive**	**Specific**
Hepatitis A	ICD[a]-10-CA	B15 (acute hepatitis A) or B19 (unspecified viral hepatitis) or A09 (diarrhea and gastroenteritis of presumed infectious origin) or A08.3 (other viral enteritis) or A08.4 (viral intestinal infection, unspecified) or A08.5 (other specified intestinal infections)	B15
	OHIP[b]	070 (viral hepatitis) or 009 (diarrhea, gastro-enteritis, viral gastro-enteritis) or 079 (other viral diseases) or 136 (other infectious or parasitic diseases) or 787 (anorexia, nausea and vomiting, heartburn, dysphagia, hiccough, hematemesis, jaundice, ascites, abdominal pain, melena, masses)	070
Malaria	ICD-10-CA	B50–B54 (malarial disease) or P37.3, P37.4 (congenital malaria) or B64 (unspecified protozoal disease)	B50–4
	OHIP	062 (Mosquito-borne viral encephalitis) or 136 (other infectious or parasitic diseases) or 781 (leg cramps, leg pain, muscle pain, joint pain, arthralgia, joint swelling, masses) or 784 (headache) or 787 (anorexia, nausea and vomiting, heartburn, dysphagia, hiccough, hematemesis, jaundice, ascites, abdominal pain, melena, masses)	062
Enteric Fever	ICD-10-CA	A01 (typhoid and paratyphoid fevers) or A02.1 (salmonella sepsis) or A02.9 (salmonella infection, unspecified) or A04.9 (bacterial intestinal infection, unspecified) or A05.9 (bacterial foodborne intoxication, unspecified) or A09 (diarrhea and gastroenteritis of presumed infectious origin) or A49.9 (bacterial infection, unspecified)	A01
	OHIP	002 (typhoid and paratyphoid fevers) or 009 or 003 (other salmonella infections) or 005 (food poisoning) or 136 or 784 or 787	002

[a] ICD, International Classification of Disease. [b] OHIP, Ontario's universal health insurance plan claims database for reimbursement of outpatient physician services.
