# Supplementary material for: Accuracy of health administrative data to identify cases of reportable travel or migration-related infectious diseases in Ontario, Canada
Source: PLoS One. 2018 Nov 7;13(11):e0207030. doi: 10.1371/journal.pone.0207030 (PMC6221317; doi:10.1371/journal.pone.0207030)
Supplement: S2 File — (PDF) [file pone.0207030.s002.pdf]

Supporting Information File 2: Dataset creation plan for health administrative data (study) cohort

Project Initiation<br>This Section must be Completed Prior to Project Dataset(s) Creation	
**Project Title:**	Measuring the burden of imported infectious diseases - Objective 1
**Project TRIM number:**	2016 0900 723 000
**Research Program:**	PCPH
**Site:**	ICES Central
**Project Objectives:**	*Insert Project Objectives as listed in the approved ICES Project PIA*<br>1. To evaluate the extent to which health administrative data can be used to accurately identify cases of travel-related disease in Ontario, and to measure the validity of diagnostic codes through comparison with reportable disease surveillance data.
**ICES Project PIA Initial Approval Date:**	*The ICES Employee or agent who is responsible for creating the Project Dataset(s) is responsible for ensuring there is an approved ICES Project PIA and verifying the date of approval prior to creating the Project Dataset(s)*<br>2015-May-26
**Principal Investigator (PI):**	Rachel Savage
**Is the PI an ICES Student/Trainee?**	☒ ICES Student ☐ ICES Fellow ☐ ICES Post-Doctoral Trainee ☐ Visiting Scholar
**Responsible ICES Scientist:**	*Name the Responsible ICES Scientist if the PI is not a Full Status ICES Scientist*<br>Dr. Laura Rosella
**Project Team Member(s) Responsible for Project Dataset Creation and/or Statistical Analysis (list all):**	*The person(s) named (ICES Analyst, Appointed Analyst, Analytic Epidemiologist, PI, and/or Student) are responsible for creating the Project Dataset(s) and/or statistical analysis*<br>Laura Holder (ICES analyst)<br>Rachel Savage (student)
**Other ICES Research Practice Project Team Members (list all):**	Alex Kopp (methodologist)
**Confirmation that DCP is consistent with Project Objectives:**	*The following individuals must confirm that the ICES Data provided for in this DCP is relevant (e.g., with respect to cohort, timeframe, and variables) and required to achieve the Project Objectives stated in the ICES Project PIA <u>prior to initial Project Dataset creation</u>: 1) PI; 2) Responsible ICES Scientist if the PI is not a Full Status ICES Scientist, or a second ICES Scientist or the Scientific Program Lead if the PI is creating both the DCP and the Project Dataset[s]; 3) ICES Research Practice Staff creating the DCP; and 4) ICES Analytic Staff (ICES Employee or agent responsible for creating the Project Dataset[s]).*<br>***Principal Investigator*** Rachel Savage 2015-Mar-27<br>***Responsible ICES Scientist or Second ICES Scientist/Lead*** Laura Rosella 2015-Apr-09<br>***ICES Research Practice Staff Creating the DCP*** Rachel Savage 2015-Mar-27<br>***ICES Analytic Staff*** Laura Holder 2016-Nov-08
**Designated ICES Research Practice Staff accountable for Project Documentation:**	*The person named (ICES staff) is accountable for ensuring that the approved ICES Project PIA, PIA Amendments, and DCP are saved on the T Drive, ensuring PIA Amendments are submitted as required, ensuring DCP Amendments are documented, and sharing the final DCP with the PI/Responsible ICES Scientist at project completion*<br>Rachel Savage, Laura Holder, Erika Yates

Project Initiation
This Section must be Completed Prior to Project Dataset(s) Creation

**DCP Creation Date and Author:**	*Date DCP was finalized prior to Project Dataset(s) creation*	*Name of person who created the DCP*
	Date	***Name***
	2015-Mar-27	Rachel Savage

ICES Data
This Section must be Completed Prior to Project Dataset(s) Creation

The ICES Employee or agent who is responsible for creating the Project Dataset(s) must ensure that this list includes only data listed in the ICES Project PIA	*Changes to this list after initial ICES Project PIA approval require a PIA Amendment*	*Mandatory for all datasets that are available by individual year*
**List of ICES Data (Acronyms)**	***Dataset***	***Years (where applicable)***
	**CIHI DAD**	2009 to 2015 (2008/09 to 2014/15)
	**CIHI SDS**	2009 to 2015 (2008/09 to 2014/15)
	**NACRS**	2009 to 2015 (2008/09 to 2014/15)
	**OHIP**	2009 to 2015 (2008/09 to 2014/15)
	**RPBD**	2009 to 2015
	**CIC / IRCC**	1985 to 2012

Project Amendments and Reconciliation

**ICES Project PIA Amendment History:**	*Privacy approval date*	*Person who submitted amendment*	*Note that any changes to the list of ICES Data or Project Objectives require a PIA Amendment*
	Date	***Name***	***Amendment***
	2016-Nov-16	Rachel Savage	Revised project start and end dates
	2018-Mar-27	Rachel Savage	Revised project completion date
**DCP Amendment History:**	*Date DCP amended*	*Person who made the DCP amendment*	*Note that any DCP amendments involving changes to the list of ICES Data or Project Objectives require a PIA Amendment*
	Date	***Name***	***Amendment***
	2016-11-08	Rachel Savage	Modified accrual start/end section and OHIP eligibility added as an other variable
**Date Programs/DCP reconciled**	*The person(s) creating the dataset and/or analyzing the data are responsible for ensuring that the final DCP reflects the final program(s) when the project is completed*		
	yyyy-mon-dd		

Project Cohort

**Study Design**	☐ Cohort study	☐ Matched cohort study	☐ Case-control study

Project Cohort

	☐ Cross-sectional study ☒ Other (specify): Validation
**Index Event / Inclusion Criteria**	Index Date: Date of first health care encounter with a travel-related diagnostic code (listed in Appendix A) during study accrual period. For hospitalization data, use admission date, not discharge date.<br><br>Inclusion Criteria: Patients with presumed travel-related illness (test positives) based on diagnostic codes listed in Appendix A during study accrual period.
**Estimated Size of Cohort**	150-200 confirmed cases in reference standard; 200-300 test positives

Exclusions (in order)

Step	Description
1	Invalid IKN
2	Missing postal code
3	Do not exclude but flag if previous health care encounter for a travel-related illness (see Appendix A) in past 90 to 730 days (between 3-24 months prior)
4	Postal code is outside of Peel region, unless the individual is in iPHIS data set. Then create flag to indicate if postal code is within Peel region or not.

Project Time Frame Definitions

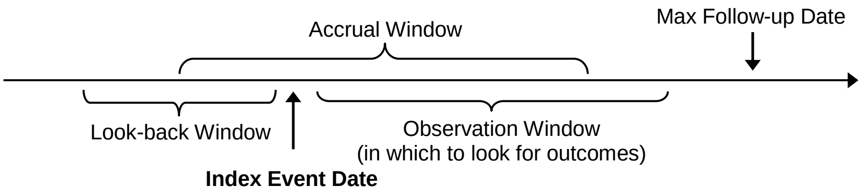

**Accrual Start/End Dates**	To select the health administrative cohort:<br>Accrual start date: November 20, 2011<br>Accrual end date: February 11, 2015
**Max Follow-up Date**	February 11, 2015
**When does observation window terminate?**	February 11, 2015
**Lookback Window(s)**	Number of days to look back to exclude those patients whose symptoms/illness are likely to be chronic and non-travel related: 3 to 24 months prior to index event date (i.e. days -90 to -730)

Variable Definitions

**Main Exposure or Risk Factor**	"Test Positives":<br>• Identify all patients with a health care encounter for a travel-related illness (defined as a patient with a diagnostic code as outlined in Appendix A) within accrual period<br>• Restrict to patients who reside in Peel Region (based on postal code in RPDB) unless in iPHIS data set. Retain all patients who are in iPHIS data set, regardless

Variable Definitions

	of region, but create a flag to identify those with a Peel region postal code vs. not.
**Primary Outcome Definition**	Peel Public Health's reportable disease data set of confirmed travel cases, known as iPHIS, has been probabilistically linked to the RPDB, assigning an IKN to all cases. To assess outcome, use IKN to determine if patient is present in both (iPHIS and health administrative data), or only one data set.<br><br>The following classifications will be made at the individual level, not the encounter level (recognizing that a patient may have multiple encounters during study accrual period):<br><br>True Positive<br>• Defined as a patient who is "test positive" (i.e. identified to have a travel-related illness in the health administrative data) AND is a confirmed case in iPHIS<br><br>False Positive<br>• Defined as a patient who is "test positive" AND is <u>not</u> in iPHIS<br><br>False Negative<br>• Defined as a confirmed case in iPHIS AND is not identified as "test positive" from the health administrative data
**Secondary Outcome Definition(s)**	
**Baseline Characteristics**	Immigrant Status - if patient is present in the CIC data (1985-2012)<br>Age at cohort entry date (RPDB)<br>Sex (RPDB)
**Other Variables**	OHIP eligible (year 1, 2, 3)<br>• Flag whether patient was eligible for OHIP in the year prior to, two years prior to, and three years prior to the case report date in iPHIS (this will help identify whether cases did not seek health care because they were ineligible)

Analysis Plan and Dummy Tables

Reference Standard
The validity of the diagnostic codes to accurately identify a case of travel-related disease will be assessed for three disease groups (hepatitis A, malaria, and enteric fever) by comparison to laboratory confirmed cases of infectious disease reported to Peel Public Health and captured in iPHIS (the reportable disease registry) as the reference standard. This will be achieved through a probabilistic linkage via unique patient identifiers (first and last name, date of birth, sex, address) to the disease-specific cohorts of presumed travel-related cases from the health administrative data.

Measures of Validity
Two unadjusted measures of validity of the diagnostic codes will be calculated for each disease and layer of codes: positive predictive value (PPV), and sensitivity (**Table 1**). Estimates will also be stratified by immigrant status and source area, as well as age, sex and health care database (sample size permitting), to assess whether there is heterogeneity in code validity across subgroups. For patients classified as immigrants, 'source area' will be determined based on the patient's country of birth and World Bank classifications: Africa, East Asia and Pacific, Europe and Central Asia, Latin America & Caribbean,

Analysis Plan and Dummy Tables

Middle East and North Africa, and South Asia.

Descriptive statistics will be used to determine the cause for discrepancies between datasets (i.e. to explore characteristics of patients classified as false positives and false negatives).

Shell Table:

Table 1: Validity of health administrative diagnostic codes to identify cases of disease X.

Health administrative cohort	Reference Standard – iPHIS	
	Disease +	No Disease -
Test + (presumed cases)	True Positive (TP)	False Positive (FP)
Test – (presumed non-cases)	False Negative (FN)	n/a

Sensitivity = TP / (TP + FN)
Positive Predictive Value = TP / (TP + FP)

***

Appendix A: Diagnostic coding criteria used for the selection of test positives and test negatives of varying expected specificity per travel-related disease

Disease	Hepatitis A		
Expected Specificity	High	Moderate	Low
ICD-10 Diagnostic Codes	B15 (acute hepatitis A)	B15 or B19 (unspecified viral hepatitis)	**B15** or **B19** or **A09** (diarrhea and gastroenteritis of presumed infectious origin) or **A08.3** (other viral enteritis) or **A08.4** (viral intestinal infection, unspecified) or **A08.5** (other specified intestinal infections)
OHIP Diagnostic Codes	070 (viral hepatitis)	070 or 009 (diarrhea, gastro-enteritis, viral gastro-enteritis)	**070** or **009** or **079** (other viral diseases) or **136** (other infectious or parasitic diseases) or **787** (anorexia, nausea and vomiting, heartburn, dysphagia, hiccough, hematemesis, *jaundice*, ascites, abdominal pain, melena, masses)
Disease	Malaria		
Expected Specificity	High	Moderate	Low
ICD-10 Diagnostic Codes	B50–B54 (malarial disease)	B50–B54 or P37.3, P37.4 (congenital malaria)	**B50–B54** (malarial disease) or **P37.3, P37.4** (congenital malaria) or **B64** (unspecified protozoal disease)
OHIP Diagnostic Codes	062 (Mosquito-borne viral encephalitis)	062	**062** or **136** (other infectious or parasitic diseases) or **781** (leg cramps, leg pain, muscle pain, joint pain, arthralgia, joint swelling, masses) or **784** (headache) or **787** (anorexia, nausea and vomiting, heartburn, dysphagia, hiccough, hematemesis, jaundice, ascites, abdominal pain, melena, masses)
Disease	Paratyphoid and Typhoid Fever		
Expected Specificity	High	Moderate	Low
ICD-10 Diagnostic Codes	A01 (typhoid and paratyphoid fevers)	A01 or A02.9 (salmonella	**A01** or **A02.1** (salmonella sepsis) or **A02.9** or **A04.9** (bacterial intestinal infection,

Analysis Plan and Dummy Tables

		infection, unspecified)	unspecified) or **A05.9** (bacterial foodborne intoxication, unspecified) or **A09** (diarrhea and gastroenteritis of presumed infectious origin) or **A49.9** (bacterial infection, unspecified)
OHIP Diagnostic Codes	002 (typhoid and paratyphoid fevers)	002 or 009	**002** or **009** or **003** (other salmonella infections) or **005** (food poisoning) or **136** or **784** or **787**

Quality Assurance Activities

**Unix Directory of SAS Programs**	
**Unix Directory of Final Dataset(s)**	*The final analytic dataset for each cohort includes all the data required to create the baseline tables and run all the models. It should include all covariates for all models such as patient risk factors, hospital characteristics, physician characteristics, exposure measures (continuous, categorical) and outcomes. It should include covariates that were considered but didn't make the final cut. This would permit an analyst to easily re-run the models in the future.*

UNIX README file available: ☐Yes ☐No

Date results of quality assurance tools shared with project team (where applicable):

	**%assign**	yyyy-mon-dd
	**%evolution**	yyyy-mon-dd
	**%dinexplore**	yyyy-mon-dd
	**%track / %exclude**	yyyy-mon-dd
	**%codebook**	yyyy-mon-dd

Additional comments:
