# Supplementary material for: Accuracy of health administrative data to identify cases of reportable travel or migration-related infectious diseases in Ontario, Canada
Source: PLoS One. 2018 Nov 7;13(11):e0207030. doi: 10.1371/journal.pone.0207030 (PMC6221317; doi:10.1371/journal.pone.0207030)
Supplement: S3 File — (PDF) [file pone.0207030.s003.pdf]

Supporting Information File 3: Capture-recapture analysis to estimate degree of underreporting of hepatitis A, malaria and enteric fever in Ontario

Objectives and methods

To estimate the reporting completeness of hepatitis A, malaria and enteric fever cases to public health through a two-source capture-recapture analysis, linking reportable disease surveillance data (iPHIS) with hospitalization data. Reportable travel or migration-related disease cases from iPHIS that were linkable (289/321) to the Registered Persons Database were used for this analysis, given that un-linkable cases would not have had a health card / been eligible to be captured in hospitalization records. The 289 cases were then linked via their scrambled health card number (ICES key number, IKN) to the medically-attended cohort of individuals with presumed travel-related disease based on the diagnostic codes outlined in the study.

We used a two-source capture-recapture method to estimate reporting completeness in iPHIS, based on iPHIS (source 1) and hospitalization records (source 2). Individuals who met the surveillance definition for a confirmed case were designated as cases in the iPHIS data, while individuals who had a hospitalization with a diagnostic code of B15 (hepatitis A), B50-54 (malaria) or A01 (enteric fever) in any of the 20 diagnostic fields (not restricted to the most responsible diagnosis field) were designated as cases in the hospitalization data. We determined the number of cases that matched between the two datasets and the numbers included only in iPHIS and only in the hospitalization data (**Fig 1**).

Two-sample capture–recapture analyses were performed. Because one of the key assumptions of capture-recapture analyses is the absence of false-positive cases, we adjusted our analysis by the positive predictive values (PPV) estimated in objective 2 for the hospitalization data [PPV=64.3% hepatitis A, 78.4% malaria, 72.9% enteric fever] [1,2]. We calculated the number of cases not captured in either data source (d), and also the estimated total universe of cases (N) (**Fig 1**). Reporting completeness was estimated by dividing the number of (linked) reported cases to iPHIS (n_1) by the total number of cases estimated from the capture-recapture analysis (N). Binomial exact methods were used to calculate 95% confidence intervals for these proportions in Stata v15.0. All other analyses were performed in Microsoft Excel.

Fig 1. Formulae to estimate the additional number of cases not captured in either data source (d) and the total number of estimated cases (N) using two-source capture-recapture analyses.

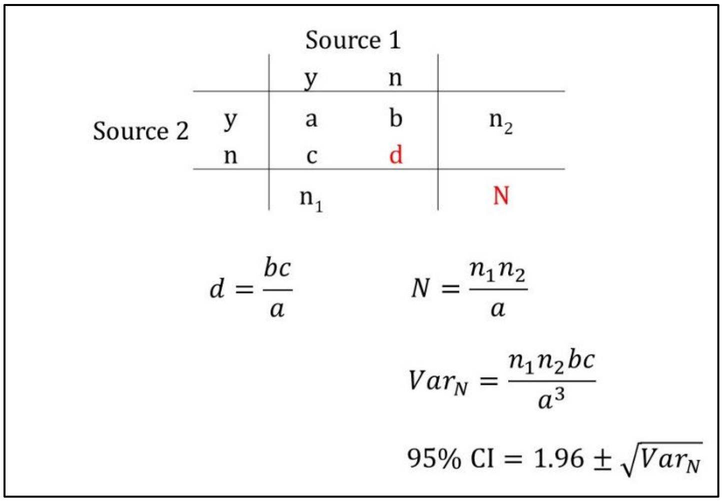

Results

Adjusted for PPV, we estimated an additional 11 hepatitis A, 12 malaria, and 14 enteric fever cases that were not captured in the reportable disease surveillance data (iPHIS) or hospitalization data, for a reporting completeness in iPHIS of 74% (95% CI 61-83%), 82% (95% CI 75-88%), and 78% (95% CI 71-84%), respectively (**Table 1**).

Table 1. Two-source capture–recapture estimates of travel-related disease cases in Peel region, 2012-2014

Disease		iPHIS only	DAD only	Both sources	Estimated total no. cases, n (95% CI)	iPHIS completeness, % (95% CI)
Hepatitis A	Crude	31	10	18	76 (59-93)	64 (53-75)
	PPV adjusted	31	6	18	67 (54-79)	74 (61-83)
Malaria	Crude	55	16	58	144 (132-156)	78 (71-85)
	PPV adjusted	55	13	58	137 (127-148)	82 (75-88)
Enteric fever	Crude	50	29	77	175 (162-188)	73 (65-79)
	PPV adjusted	50	21	77	162 (151-172)	78 (71-84)

PPV, positive predictive value. iPHIS, integrated public health reporting system. DAD, discharge abstract database for hospitalization records. CI, confidence interval.

References

1. Hook EB, Regal RR. Capture-recapture methods in epidemiology: methods and limitations. Epidemiol Rev. 1995;17(2):243-64.

2. Granerod J, Cousens S, Davies NW, Crowcroft NS, Thomas SL. New estimates of incidence of encephalitis in England. Emerg Infect Dis. 2013;19(9).
