# Supplementary material for: Accuracy of health administrative data to identify cases of reportable travel or migration-related infectious diseases in Ontario, Canada
Source: PLoS One. 2018 Nov 7;13(11):e0207030. doi: 10.1371/journal.pone.0207030 (PMC6221317; doi:10.1371/journal.pone.0207030)
Supplement: S4 File — (PDF) [file pone.0207030.s004.pdf]

Supporting Information File 4: Analytic SAS Code

```
/*Measuring validity (sensitivity and PPV) of administrative diagnostic codes
for travel-related disease*/

/*Written: Jan 31 2017 by Rachel Savage*/
/*Revised: July 6 2017*/


libname MYDATA '/sasroot/projects/pcph/p0900.723.000/02may2017';
run;

libname DATASETS '/sasroot/projects/pcph/p0900.723.000';
run;


/*Description of Reference Std (true positives)*/

data refstd;
set MYDATA.iphis_full_02may2017;
run;

proc contents data=refstd position;
run; /*321 cases*/

proc sort data=refstd;
by accurateepisodedate;
run; /*dec 20, 2011 to dec 17, 2014 - 3 years of data*/

proc sort data=refstd;
by reporteddate;
run; /*jan 6 2012 to dec 24 2014*/

proc freq data=refstd;
tables valikn;
run; /*289 with valid IKN, 32 missing - 321 cases total*/

proc freq data=refstd;
where valikn="V";
tables episodedatetype;
run; /*94.8% based on symptom onset*/


/*Check for duplicate IKNs*/
proc freq data=refstd;
tables ikn/noprint out=keylist;
run;

proc print;
where count ge 2;
run;
/*Two cases were sick twice (in two separate travel encounters) - keep all*/
/*Count as separate cases*/

proc freq data=refstd;
```

```
tables sex ageattimeofillness disease countryofbirth travelassociated
primarytravelcountry pretravelconsult interventiontype;
run;

proc freq data=refstd;
tables disease;
run;

proc freq data=refstd;
where ikn ne ' ';
tables disease;
run;


/*/

/*Compare linked and unlinked cases*/

data refstd;
set refstd;
if valikn = ' ' then valikn = 'N';
run;

proc freq data=refstd;
tables valikn;
run;

proc freq data=refstd;
by valikn;
tables city sex ageattimeofillness disease countryofbirth travelassociated
primarytravelcountry pretravelconsult interventiontype;
run;

data refstd;
set refstd;
length city2 $20;
if city = 'BRAMPTON' then city2 = 'Brampton';
else if city = 'MISSISSAUGA' then city2 = 'Mississauga';
else if city2 = ' ' then city2 = 'Other';
run;

proc freq data=refstd;
tables city2*valikn / chisq;
run;

data refstd;
set refstd;
if city in ('BRAMPTON' 'MISSISSAUGA' 'INGLEWOOD') then peel = 1;
else if peel = . then peel = 0;
run;

proc freq data=refstd;
tables peel*valikn / chisq;
run;

proc freq data=refstd;
tables sex*valikn / chisq;
```

```
run;

data refstd;
set refstd;
agegp=.;
if ageattimeofillness < 18 then agegp=1;
else if ageattimeofillness ge 18 and ageattimeofillness le 64 then agegp=2;
else if ageattimeofillness > 64 then agegp=3;
run;

proc freq data=refstd;
tables agegp*valikn / chisq;
run;

proc means median min max data=refstd;
by valikn;
var ageattimeofillness;
run;

proc NPAR1WAY data=refstd wilcoxon;
class valikn;
var ageattimeofillness;
run;

data refstd;
set refstd;
if disease in ("HEPATITIS A") then diseasegp=1;
else if disease in ("MALARIA") then diseasegp=2;
else if disease in ("PARATYPHOID FEVER" "TYPHOID FEVER") then diseasegp=3;
run;

proc freq data=refstd;
tables diseasegp*valikn / chisq norow;
run;

data refstd;
set refstd;
length cob2 $20;
if countryofbirth = 'Canada'  then cob2 = 'Canada';
else if cob2 = ' ' then cob2 = 'Other';
run;

proc freq data=refstd;
tables cob2*valikn / chisq norow;
run;

data refstd;
set refstd;
if travelassociated = 'Yes' then travel='Y';
else if travelassociated = 'No' or travelassociated = 'Unknown' then
travel='N';
else if travelassociated = 'Recent immigrant' or travelassociated = 'Visitor'
then travel='O';
run;

proc freq data=refstd;
tables travel*valikn / chisq norow;
```

```
run;

data refstd;
set refstd;
if interventiontype = 'HOSPITALIZATION' then hosp='Y';
else if interventiontype = ' ' then hosp='N';
run;

proc freq data=refstd;
tables hosp*valikn / chisq norow;
run;



/*Look at administrative health cohort created by ICES*/

data cohort;
set MYDATA.admin_codes_iphis_02may2017;
run;


/***/


/**** FALSE POSITIVES ****/


/**** ALL DISEASES FALSE POSITIVES *****/


data cohort_fp (where=(iphis=0 and first_admin_code_date ne .));
set cohort;
run;

proc sort data=cohort_fp;
by ikn first_admin_code_date;
run;

data uniqueikn;
set cohort_fp;
if first.ikn then count=0;
count + 1;
if last.ikn then output;
by ikn;
run;

/*** HEPATITIS A ***/

data cohort_fp;
set cohort_fp;
hep=0;
if     dxcode     in: ('070' '009' '079' '136' '787')  then hep=1;
else if dx10code_1 in: ('B15' 'B19' 'A09' 'A083' 'A084' 'A085') then hep=1;
else if dx10code_2 in: ('B15' 'B19' 'A09' 'A083' 'A084' 'A085') then hep=1;
else if dx10code_3 in: ('B15' 'B19' 'A09' 'A083' 'A084' 'A085') then hep=1;
run;
```

```
proc freq data=cohort_fp;
tables hep;
run;

data cohort_fp_hep (where=(hep=1));
set cohort_fp;
run;

proc sort data=cohort_fp_hep;
by dx10code_1;
run;

proc freq data=cohort_fp_hep;
tables dxcode dx10code:;
run;


data cohort_fp_hep;
set cohort_fp_hep;
length      dxcode_clean $3;
if          dxcode in: ('009') then dxcode_clean='009';
if          dxcode in: ('079') then dxcode_clean='079';
if          dxcode in: ('136') then dxcode_clean='136';
if          dxcode in: ('787') then dxcode_clean='787';
else  dxcode_clean=dxcode;
run;

data cohort_fp_hep;
set cohort_fp_hep;
length dx10code1_clean $7;
dx10code1_clean=dx10code_1;
if dx10code_1 in: ('A09') then dx10code1_clean='A09';
run;

data cohort_fp_hep;
set cohort_fp_hep;
length dx10code2_clean $7;
dx10code2_clean=dx10code_2;
if dx10code_2 in: ('A09') then dx10code2_clean='A09';
run;

data cohort_fp_hep;
set cohort_fp_hep;
length dx10code3_clean $7;
dx10code3_clean=dx10code_3;
if dx10code_3 in: ('A09') then dx10code3_clean='A09';
run;

proc freq data=cohort_fp_hep;
tables dxcode_clean dx10code1_clean dx10code2_clean dx10code3_clean;
run;


/*how many unique individuals?*/

proc sort data=cohort_fp_hep;
by ikn first_admin_code_date;
```

```
run;

data uniqueikn_hep;
set cohort_fp_hep;
if first.ikn then count=0;
count + 1;
if last.ikn then output;
by ikn;
run;

/*Look at time between first and last date in individuals with >1 episode*/

data cohort_fp_hep;
set cohort_fp_hep;
datediffo=date-first_admin_code_date;
run;

proc sort data=cohort_fp_hep;
by ikn first_admin_code_date date;
run;

data episodeperiod_hep;
set cohort_fp_hep;
if first.ikn then count=0;
count + 1;
if last.ikn then output;
by ikn;
run;

proc means mean median min max data=episodeperiod_hep;
var datediffo count;
run;

proc freq data=episodeperiod_hep;
tables datediffo count;
run;

proc univariate data=episodeperiod_hep;
var datediffo count;
histogram;
run;

/*determine how many FP patients have an admin code in lookback period (based
on first encounter)*/

proc sort data=cohort_fp_hep;
by ikn date;
run;

data lookback_hep;
set cohort_fp_hep;
if first.ikn then count=0;
count + 1;
if first.ikn then output;
by ikn;
run;
```

```
proc freq data=lookback_hep;
tables admin_code_in_lookback;
run;


/*now, flatten so one row per IKN:*/

data cohort_fp_hep (rename=(dx10code_1=dx10code1_ dx10code_2=dx10code2_
dx10code_3=dx10code3_));
set cohort_fp_hep;
run;

%MAKEWIDE(data          = cohort_fp_hep,
           out          = source_wide,
           id           = ikn,
           var          = source);

data source_wide;
set source_wide;
run;

%MAKEWIDE(data          = cohort_fp_hep,
           out          = date_wide,
           id           = ikn,
           var          = date);

data date_wide;
set date_wide;
run;

%MAKEWIDE(data          = cohort_fp_hep,
           out          = dxcode_wide,
           id           = ikn,
           var          = dxcode);

data dxcode_wide;
set dxcode_wide;
run;

%MAKEWIDE(data          = cohort_fp_hep,
           out          = dx10code1_wide,
           id           = ikn,
           var          = dx10code1_);

data dx10code1_wide;
set dx10code1_wide;
run;

%MAKEWIDE(data          = cohort_fp_hep,
           out          = dx10code2_wide,
           id           = ikn,
           var          = dx10code2_);

data dx10code2_wide;
set dx10code2_wide;
run;
```

```
%MAKEWIDE(data          = cohort_fp_hep,
           out          = dx10code3_wide,
           id           = ikn,
           var          = dx10code3_);

data dx10code3_wide;
set dx10code3_wide;
run;


/*now merge together*/
data hepa_wide_fp;
merge source_wide
date_wide
dxcode_wide
dx10code1_wide
dx10code2_wide
dx10code3_wide;
by ikn;
run;


/*1 - high specificity (h_sp)*/

data ha1;
set hepa_wide_fp;
array dxc[285] $ dxcode1-dxcode285;
array dx10c1[285] $dx10code1_1-dx10code1_285;
array dx10c2[285] $dx10code2_1-dx10code2_285;
array dx10c3[285] $dx10code3_1-dx10code3_285;
h_sp=0;
do i=1 to 285;
if           dxc[i] in:('070')              then h_sp=1;
else if dx10c1[i] in: ('B15')       then h_sp=1;
else if dx10c2[i] in: ('B15')       then h_sp=1;
else if dx10c3[i] in: ('B15')       then h_sp=1;
end;
run;

proc freq data=ha1;
tables h_sp;
run; /*15,591 meet this criteria */


/*2 - moderate specificity (m_sp)*/

data ha1;
set ha1;
array dxc[285] $ dxcode1-dxcode285;
array dx10c1[285] $dx10code1_1-dx10code1_285;
array dx10c2[285] $dx10code2_1-dx10code2_285;
array dx10c3[285] $dx10code3_1-dx10code3_285;
m_sp=0;
do i=1 to 285;
if           dxc[i] in:('009')              then m_sp=1;
else if dx10c1[i] in: ('B19')       then m_sp=1;
else if dx10c2[i] in: ('B19')       then m_sp=1;
```

```
else if dx10c3[i] in: ('B19')       then m_sp=1;
end;
run;

proc freq data=ha1;
tables m_sp;
run;

/*3 - low specificity (l_sp)*/

data ha1;
set ha1;
array dxc[285] $ dxcode1-dxcode285;
array dx10c1[285] $dx10code1_1-dx10code1_285;
array dx10c2[285] $dx10code2_1-dx10code2_285;
array dx10c3[285] $dx10code3_1-dx10code3_285;
l_sp=0;
do i=1 to 285;
if    dxc[i] in: ('079' '136' '787') then l_sp=1;
else if dx10c1[i] in: ('A09' 'A083' 'A084' 'A085') then l_sp=1;
else if dx10c2[i] in: ('A09' 'A083' 'A084' 'A085') then l_sp=1;
else if dx10c3[i] in: ('A09' 'A083' 'A084' 'A085') then l_sp=1;
end;
run;

proc freq data=ha1;
tables l_sp;
run;



data ha1;
set ha1;
array dxc[285] $ dxcode1-dxcode285;
c_070=0;
do i=1 to 285;
if    dxc[i] in: ('070') then c_070=1;
end;
run;

data ha1;
set ha1;
array dx10c1[285] $dx10code1_1-dx10code1_285;
array dx10c2[285] $dx10code2_1-dx10code2_285;
array dx10c3[285] $dx10code3_1-dx10code3_285;
c_B15=0;
do i=1 to 285;
if dx10c1[i] in: ('B15')            then c_B15=1;
else if dx10c2[i] in: ('B15')       then c_B15=1;
else if dx10c3[i] in: ('B15')       then c_B15=1;
end;
run;

data ha1;
set ha1;
array dx10c1[285] $dx10code1_1-dx10code1_285;
array dx10c2[285] $dx10code2_1-dx10code2_285;
```

```
array dx10c3[285] $dx10code3_1-dx10code3_285;
c_A084=0;
do i=1 to 285;
if dx10c1[i] in: ('A084')          then c_A084=1;
else if dx10c2[i] in: ('A084')     then c_A084=1;
else if dx10c3[i] in: ('A084')     then c_A084=1;
end;
run;

data ha1;
set ha1;
array dx10c1[285] $dx10code1_1-dx10code1_285;
array dx10c2[285] $dx10code2_1-dx10code2_285;
array dx10c3[285] $dx10code3_1-dx10code3_285;
c_A09=0;
do i=1 to 285;
if dx10c1[i] in: ('A09')           then c_A09=1;
else if dx10c2[i] in: ('A09')      then c_A09=1;
else if dx10c3[i] in: ('A09')      then c_A09=1;
end;
run;

data ha1;
set ha1;
array dx10c1[285] $dx10code1_1-dx10code1_285;
array dx10c2[285] $dx10code2_1-dx10code2_285;
array dx10c3[285] $dx10code3_1-dx10code3_285;
c_B19=0;
do i=1 to 285;
if dx10c1[i] in: ('B19')           then c_B19=1;
else if dx10c2[i] in: ('B19')      then c_B19=1;
else if dx10c3[i] in: ('B19')      then c_B19=1;
end;
run;

data ha1;
set ha1;
array dxc[285] $ dxcode1-dxcode285;
array dx10c1[285] $dx10code1_1-dx10code1_285;
array dx10c2[285] $dx10code2_1-dx10code2_285;
array dx10c3[285] $dx10code3_1-dx10code3_285;
code009=0;
do i=1 to 285;
if dxc[i] in:('009')         then code009=1;
end;
run;

data ha1;
set ha1;
array dxc[285] $ dxcode1-dxcode285;
array dx10c1[285] $dx10code1_1-dx10code1_285;
array dx10c2[285] $dx10code2_1-dx10code2_285;
array dx10c3[285] $dx10code3_1-dx10code3_285;
code079=0;
do i=1 to 285;
if dxc[i] in:('079')         then code079=1;
end;
```

```
run;

data ha1;
set ha1;
array dxc[285] $ dxcode1-dxcode285;
array dx10c1[285] $dx10code1_1-dx10code1_285;
array dx10c2[285] $dx10code2_1-dx10code2_285;
array dx10c3[285] $dx10code3_1-dx10code3_285;
code787=0;
do i=1 to 285;
if    dxc[i] in:('787')       then code787=1;
end;
run;


data ha1;
set ha1;
array dxc[285] $ dxcode1-dxcode285;
array dx10c1[285] $dx10code1_1-dx10code1_285;
array dx10c2[285] $dx10code2_1-dx10code2_285;
array dx10c3[285] $dx10code3_1-dx10code3_285;
codea08=0;
do i=1 to 285;
if dx10c1[i] in: ('A083' 'A084' 'A085')   then codea08=1;
else if dx10c2[i] in: ('A083' 'A084' 'A085') then codea08=1;
else if dx10c3[i] in: ('A083' 'A084' 'A085') then codea08=1;
end;
run;

proc freq data=ha1;
tables c_070 c_B15 c_A084 c_A09 c_B19 code009 code079 code787 codea08;
run;


/*Now merge with admin cohort to describe sex, age, immigrant status, admin
in lookback, source, etc for Table 3*/

data ha1;
set ha1;
array s[285] $ source1-source285;
hosp=0;
do i=1 to 285;
      if    s[i] in: ('DAD') then hosp=1;
      end;
      run;

data ha1;
set ha1;
array s[285] $ source1-source285;
ed=0;
do i=1 to 285;
      if    s[i] in: ('NACRS') then ed=1;
      end;
      run;

data ha1;
set ha1;
```

```
array s[285] $ source1-source285;
pc=0;
do i=1 to 285;
      if    s[i] in: ('OHIP') then pc=1;
      end;
      run;

data ha1;
set ha1;
array s[285] $ source1-source285;
sds=0;
do i=1 to 285;
      if    s[i] in: ('SDS') then sds=1;
      end;
      run;

proc freq data=ha1;
tables hosp ed pc sds;
run;


data ha1;
set ha1;
if    (hosp=1 or ed=1 or sds=1) and pc=1  then codes='Both';
else if  pc=1                             then codes='OHIP';
else if  hosp=1 or ed=1 or sds=1          then codes='ICD10';
run;

proc freq data=ha1;
tables codes;
run;


/*now merge with episodeperiod_hep to get datediffo and count summaries*/

data epi_hep (keep=ikn datediffo count);
set episodeperiod_hep;
run;

proc sort data=epi_hep;
by ikn;
run;

data ha2_summary;
merge ha1(in=hep) epi_hep (in=period);
by ikn;
if hep and period;
run;

proc means mean median min max data=ha2_summary;
var datediffo count;
run;


/*now merge with admin data - first create data set from original cohort with
1 obs per patient based on first health encounter*/
proc sort data=cohort;
```

```
by ikn first_admin_code_date;
run;

data ha3 (keep=ikn sex age_cohort_entry cic_immigrant
admin_code_in_lookback);
set cohort;
if first.ikn then output;
by ikn;
run;

data ha4;
merge ha3 (in=cohort) ha2_summary(in=hep);
by ikn;
if cohort and hep;
run;


proc freq data=ha4;
tables sex cic_immigrant admin_code_in_lookback;
run;

proc means median min max data=ha4;
var age_cohort_entry;
run;


/*now save as a permanent dataset - all hep A FP*/
/*can filter h_sp=1 to get only those with high spec codes*/

data DATASETS.final_hepa_fp_02may2017;
set ha4;
run;



/*HIGH SPEC ANALYSIS*/

data ha4;
set DATASETS.final_hepa_fp_02may2017;
run;


data ha6 (where=(h_sp=1));
set ha4;
run;

proc freq data=ha6;
tables sex cic_immigrant admin_code_in_lookback hosp ed pc sds;
run;

proc freq data=ha6;
tables c_070*c_B15;
run;

proc means mean median min max data=ha6;
var age_cohort_entry datediffo count;
run;
```

```
proc freq data=ha6;
tables c_070 c_B15 c_A084 c_A09 c_B19 code009 code079 code787 codea08;
run;


/*describe those hosp*/
data ha_hosp (where=(hosp=1));
set ha6;
run;

proc freq data=ha_hosp;
tables sex cic_immigrant admin_code_in_lookback;
run;

proc means mean median min max data=ha_hosp;
var age_cohort_entry;
run;

proc freq data=ha1;
tables c_070 c_B15 c_A084 c_A09 c_B19 code009 code079 code787 codea08;
run;



/***** MALARIA *****/

data cohort_fp;
set cohort_fp;
mal=0;
      if    dxcode     in: ('062' '136' '781' '784' '787')  then mal=1;
      else if dx10code_1 in: ('B5' 'P373' 'P374' 'B64')           then mal=1;
      else if dx10code_2 in: ('B5' 'P373' 'P374' 'B64')           then mal=1;
      else if dx10code_3 in: ('B5' 'P373' 'P374' 'B64')           then mal=1;
      run;

proc freq data=cohort_fp;
tables mal;
run;

data cohort_fp_mal (where=(mal=1));
set cohort_fp;
run;

proc sort data=cohort_fp_mal;
by dx10code_1;
run;

proc freq data=cohort_fp_mal;
tables dxcode dx10code:;
run;


data cohort_fp_mal;
set cohort_fp_mal;
length      dxcode_clean $3;
if          dxcode in: ('136') then dxcode_clean='136';
```

```
if          dxcode in: ('781') then dxcode_clean='781';
if          dxcode in: ('784') then dxcode_clean='784';
if          dxcode in: ('787') then dxcode_clean='787';
else  dxcode_clean=dxcode;
run;


data cohort_fp_mal;
set cohort_fp_mal;
length dx10code1_clean $7;
dx10code1_clean=dx10code_1;
if dx10code_1 in: ('B5')      then dx10code1_clean='B50-B54';
run;

data cohort_fp_mal;
set cohort_fp_mal;
length dx10code2_clean $7;
dx10code2_clean=dx10code_2;
if dx10code_2 in: ('B5') then dx10code2_clean='B50-B54';
run;

data cohort_fp_mal;
set cohort_fp_mal;
length dx10code3_clean $7;
dx10code3_clean=dx10code_3;
if dx10code_3 in: ('B5') then dx10code3_clean='B50-B54';
run;

proc freq data=cohort_fp_mal;
tables dxcode_clean dx10code1_clean dx10code2_clean dx10code3_clean;
run;



/*how many unique individuals?*/

proc sort data=cohort_fp_mal;
by ikn first_admin_code_date;
run;

data uniqueikn_mal;
set cohort_fp_mal;
if first.ikn then count=0;
count + 1;
if last.ikn then output;
by ikn;
run; /*665,912 unique IKNs */


/*Look at time between first and last date in individuals with >1 episode*/

data cohort_fp_mal;
set cohort_fp_mal;
datediffo=date-first_admin_code_date;
run;

proc sort data=cohort_fp_mal;
```

```
by ikn first_admin_code_date date;
run;

data episodeperiod_mal;
set cohort_fp_mal;
if first.ikn then count=0;
count + 1;
if last.ikn then output;
by ikn;
run;

proc sort data=episodeperiod_mal;
by datediffo;
run;

proc means mean median min max data=episodeperiod_mal;
var datediffo count;
run;

proc freq data=episodeperiod_mal;
tables datediffo count;
run;

proc univariate data=episodeperiod_mal;
var datediffo count;
histogram;
run;


/*determine how many FP patients have an admin code in lookback period (based
on first encounter)*/

proc sort data=cohort_fp_mal;
by ikn date;
run;

data lookback_mal;
set cohort_fp_mal;
if first.ikn then count=0;
count + 1;
if first.ikn then output;
by ikn;
run;

proc freq data=lookback_mal;
tables admin_code_in_lookback;
run;



/*now, flatten so one row per IKN:*/

data cohort_fp_mal (rename=(dx10code_1=dx10code1_ dx10code_2=dx10code2_
dx10code_3=dx10code3_));
set cohort_fp_mal;
run;
```

```
%MAKEWIDE(data          = cohort_fp_mal,
           out          = source_wide,
           id           = ikn,
           var          = source);

data source_wide;
set source_wide;
run;

%MAKEWIDE(data          = cohort_fp_mal,
           out          = date_wide,
           id           = ikn,
           var          = date);

data date_wide;
set date_wide;
run;

%MAKEWIDE(data          = cohort_fp_mal,
           out          = dxcode_wide,
           id           = ikn,
           var          = dxcode);

data dxcode_wide;
set dxcode_wide;
run;

%MAKEWIDE(data          = cohort_fp_mal,
           out          = dx10code1_wide,
           id           = ikn,
           var          = dx10code1_);

data dx10code1_wide;
set dx10code1_wide;
run;

%MAKEWIDE(data          = cohort_fp_mal,
           out          = dx10code2_wide,
           id           = ikn,
           var          = dx10code2_);

data dx10code2_wide;
set dx10code2_wide;
run;

%MAKEWIDE(data          = cohort_fp_mal,
           out          = dx10code3_wide,
           id           = ikn,
           var          = dx10code3_);

data dx10code3_wide;
set dx10code3_wide;
run;


/*now merge together*/
data mal_wide_fp;
```

```
merge source_wide
date_wide
dxcode_wide
dx10code1_wide
dx10code2_wide
dx10code3_wide;
by ikn;
run;


/*1 - highly specificity (h_sp)*/

data m1;
set mal_wide_fp;
array dxc[298] $ dxcode1-dxcode298;
array dx10c1[298] $dx10code1_1-dx10code1_298;
array dx10c2[298] $dx10code2_1-dx10code2_298;
array dx10c3[298] $dx10code3_1-dx10code3_298;
h_sp=0;
do i=1 to 298;
      if           dxc[i] in:('062') then h_sp=1;
      else if dx10c1[i] in: ('B5')  then h_sp=1;
      else if dx10c2[i] in: ('B5')  then h_sp=1;
      else if dx10c3[i] in: ('B5')  then h_sp=1;
      end;
      run;

proc freq data=m1;
tables h_sp;
run; /*111 meet this criteria*/


/*2 - moderate specificity (m_sp)*/

data m1;
set m1;
array dxc[298] $ dxcode1-dxcode298;
array dx10c1[298] $dx10code1_1-dx10code1_298;
array dx10c2[298] $dx10code2_1-dx10code2_298;
array dx10c3[298] $dx10code3_1-dx10code3_298;
m_sp=0;
do i=1 to 298;
      if           dxc[i] in:('062')             then m_sp=1;
      else if dx10c1[i] in: ('P373' 'P374')     then m_sp=1;
      else if dx10c2[i] in: ('P373' 'P374')     then m_sp=1;
      else if dx10c3[i] in: ('P373' 'P374')     then m_sp=1;
      end;
      run;

proc freq data=m1;
tables m_sp;
run;



/*3 - low specificity (l_sp)*/
```

```
data m1;
set m1;
array dxc[298] $ dxcode1-dxcode298;
array dx10c1[298] $dx10code1_1-dx10code1_298;
array dx10c2[298] $dx10code2_1-dx10code2_298;
array dx10c3[298] $dx10code3_1-dx10code3_298;
l_sp=0;
do i=1 to 298;
      if           dxc[i] in: ('136' '781' '784' '787')       then l_sp=1;
      else if dx10c1[i] in: ('B64')                           then l_sp=1;
      else if dx10c2[i] in: ('B64')                           then l_sp=1;
      else if dx10c3[i] in: ('B64')                           then l_sp=1;
      end;
      run;

proc freq data=m1;
tables l_sp;
run;


/*code distribution for low specificity FP*/

data m1;
set m1;
array dxc[298] $ dxcode1-dxcode298;
c_062=0;
do i=1 to 298;
      if    dxc[i] in: ('062') then c_062=1;
      end;
      run;

data m1;
set m1;
array dx10c1[298] $dx10code1_1-dx10code1_298;
array dx10c2[298] $dx10code2_1-dx10code2_298;
array dx10c3[298] $dx10code3_1-dx10code3_298;
c_B5=0;
do i=1 to 298;
      if dx10c1[i] in: ('B5')       then c_B5=1;
      else if dx10c2[i] in: ('B5')  then c_B5=1;
      else if dx10c3[i] in: ('B5')  then c_B5=1;
      end;
      run;

data m1;
set m1;
array dxc[298] $ dxcode1-dxcode298;
c_136=0;
do i=1 to 298;
      if    dxc[i] in: ('136') then c_136=1;
      end;
      run;

data m1;
set m1;
array dxc[298] $ dxcode1-dxcode298;
c_781=0;
```

```
do i=1 to 298;
      if    dxc[i] in: ('781') then c_781=1;
      end;
      run;

data m1;
set m1;
array dxc[298] $ dxcode1-dxcode298;
c_787=0;
do i=1 to 298;
      if    dxc[i] in: ('787') then c_787=1;
      end;
      run;

data m1;
set m1;
array dx10c1[298] $dx10code1_1-dx10code1_298;
array dx10c2[298] $dx10code2_1-dx10code2_298;
array dx10c3[298] $dx10code3_1-dx10code3_298;
c_B64=0;
do i=1 to 298;
      if dx10c1[i] in: ('B64')            then c_B64=1;
      else if dx10c2[i] in: ('B64')       then c_B64=1;
      else if dx10c3[i] in: ('B64')       then c_B64=1;
      end;
      run;

proc freq data=m1;
tables c_062 c_B5 c_136 c_781 c_787;
run;

proc freq data=m1;
tables c_B64;
run;


data m1;
set m1;
array s[298] $ source1-source298;
hosp=0;
do i=1 to 298;
      if    s[i] in: ('DAD') then hosp=1;
      end;
      run;

data m1;
set m1;
array s[298] $ source1-source298;
ed=0;
do i=1 to 298;
      if    s[i] in: ('NACRS') then ed=1;
      end;
      run;

data m1;
set m1;
array s[298] $ source1-source298;
```

```
pc=0;
do i=1 to 298;
       if    s[i] in: ('OHIP') then pc=1;
       end;
       run;

data m1;
set m1;
array s[298] $ source1-source298;
sds=0;
do i=1 to 298;
       if    s[i] in: ('SDS') then sds=1;
       end;
       run;

proc freq data=m1;
tables hosp ed pc sds;
run;



/*now merge with episodeperiod_mal to get datediffo and count summaries*/

data epi_mal (keep=ikn datediffo count);
set episodeperiod_mal;
run;

proc sort data=epi_mal;
by ikn;
run;

data m2_summary;
merge epi_mal (in=period) m1(in=mal);
by ikn;
if period and mal;
run;

proc means mean median min max data=m2_summary;
var datediffo count;
run;


/*now merge back in with original cohort to describe characteristics of FPs*/
/*can filter h_sp=1 to describe high spec FP*/


/*create data set from original cohort with 1 obs per patient based on first
health encounter*/
proc sort data=cohort;
by ikn first_admin_code_date;
run;

data uniqueikn;
set cohort;
if first.ikn then count=0;
count + 1;
if last.ikn then output;
```

```
by ikn;
run;

data m3 (keep=ikn cohort_entry age_cohort_entry sex cic_immigrant cic_fsrce
admin_code_in_lookback);
set uniqueikn;
run;

data m4;
merge m3 (in=cohort) m2_summary(in=mal);
by ikn;
if cohort and mal;
run;

data m4;
set m4;
agegp=.;
if     age_cohort_entry < 18 then agegp=1;
else if age_cohort_entry ge 18 and age_cohort_entry le 64 then agegp=2;
else if age_cohort_entry ge 65 then agegp=3;
run;

proc univariate data=m4;
var age_cohort_entry;
run;

proc freq data=m4;
tables agegp sex cic_immigrant cic_fsrce admin_code_in_lookback;
run;


/*create perm dataset*/
data DATASETS.final_mal_fp_02may2017;
set m4;
run;

/*forgot to run code freq - July 6, 2017*/
data m;
set DATASETS.final_mal_fp_02may2017;
run;

data m (where=(h_sp=1));
set m;
run;

proc freq data=m;
tables c_B5 c_062 c_136 c_781 c_787;
run;



/**HIGH SPEC ANALYSIS**/

data m5 (where=(h_sp=1));
set m4;
run;
```

```
proc univariate data=m5;
var age_cohort_entry;
run;

proc freq data=m5;
tables agegp sex cic_immigrant cic_fsrce admin_code_in_lookback;
run;

proc means mean median min max data=m5;
var datediffo count;
run;

proc freq data=m5;
tables hosp ed pc sds;
run;

proc freq data=m5;
tables c_062*c_B5;
run;


/*describe codes of those hosp*/
data m4_hosp (where=(hosp=1));
set m4;
run;

proc freq data=m4_hosp;
tables c_B5;
run;

proc means mean median min max data=m4_hosp;
var age_cohort_entry;
run;

proc freq data=m4_hosp;
tables agegp sex cic_immigrant cic_fsrce admin_code_in_lookback;
run;




/**********TYPHOID+PARATYPHOID FEVER**********/

data cohort_fp;
set cohort_fp;
tpt=0;
      if dxcode in: ('002' '009' '003' '005' '136' '784' '787') then tpt=1;
      else if dx10code_1 in: ('A01' 'A029' 'A021' 'A049' 'A059' 'A09' 'A499')
then tpt=1;
      else if dx10code_2 in: ('A01' 'A029' 'A021' 'A049' 'A059' 'A09' 'A499')
then tpt=1;
      else if dx10code_3 in: ('A01' 'A029' 'A021' 'A049' 'A059' 'A09' 'A499')
then tpt=1;
      run;

proc freq data=cohort_fp;
tables tpt;
```

```
run;

data cohort_fp_tpt (where=(tpt=1));
set cohort_fp;
run;

proc freq data=cohort_fp_tpt;
tables dxcode dx10code:;
run;


data cohort_fp_tpt;
set cohort_fp_tpt;
length      dxcode_clean $3;
if          dxcode in: ('009') then dxcode_clean='009';
if          dxcode in: ('136') then dxcode_clean='136';
if          dxcode in: ('784') then dxcode_clean='784';
if          dxcode in: ('787') then dxcode_clean='787';
else  dxcode_clean=dxcode;
run;

data cohort_fp_tpt;
set cohort_fp_tpt;
length      dx10code1_clean $7;
            dx10code1_clean=dx10code_1;
if          dx10code_1 in: ('A01')  then dx10code1_clean='A01';
else if     dx10code_1 in: ('A09')  then dx10code1_clean='A09';
run;

data cohort_fp_tpt;
set cohort_fp_tpt;
length      dx10code2_clean $7;
            dx10code2_clean=dx10code_2;
if          dx10code_2 in: ('A01')  then dx10code2_clean='A01';
else if     dx10code_2 in: ('A09')  then dx10code2_clean='A09';
run;

data cohort_fp_tpt;
set cohort_fp_tpt;
length      dx10code3_clean $7;
            dx10code3_clean=dx10code_3;
if          dx10code_3 in: ('A01')  then dx10code3_clean='A01';
else if     dx10code_3 in: ('A09')  then dx10code3_clean='A09';
run;

proc freq data=cohort_fp_tpt;
tables dxcode_clean dx10code1_clean dx10code2_clean dx10code3_clean;
run;



/*how many unique individuals?*/

proc sort data=cohort_fp_tpt;
by ikn first_admin_code_date;
run;
```

```
data uniqueikn_tpt;
set cohort_fp_tpt;
if first.ikn then count=0;
count + 1;
if last.ikn then output;
by ikn;
run; /*541,980unique IKNs*/


/*Look at time between first and last date in individuals with >1 episode*/

data cohort_fp_tpt;
set cohort_fp_tpt;
datediffo=date-first_admin_code_date;
run;

proc sort data=cohort_fp_tpt;
by ikn first_admin_code_date date;
run;

data episodeperiod_tpt;
set cohort_fp_tpt;
if first.ikn then count=0;
count + 1;
if last.ikn then output;
by ikn;
run;

proc sort data=episodeperiod_tpt;
by datediffo;
run;

proc means mean median min max data=episodeperiod_tpt;
var datediffo count;
run;

proc freq data=episodeperiod_tpt;
tables datediffo count;
run;

proc univariate data=episodeperiod_tpt;
var datediffo;
histogram;
run;


/*determine how many FP patients have an admin code in lookback period (based
on first encounter)*/

proc sort data=cohort_fp_tpt;
by ikn date;
run;

data lookback_tpt;
set cohort_fp_tpt;
if first.ikn then count=0;
count + 1;
```

```
if first.ikn then output;
by ikn;
run;

proc freq data=lookback_tpt;
tables admin_code_in_lookback;
run;


/*Now go back and describe the constellation of codes PER unique IKN*/

data cohort_fp_tpt (rename=(dx10code_1=dx10code1_ dx10code_2=dx10code2_
dx10code_3=dx10code3_));
set cohort_fp_tpt;
run;


/*need to flatten only one row per ikn/individual level*/

%MAKEWIDE(data          = cohort_fp_tpt,
          out           = source_wide,
          id            = ikn,
          var           = source);

data source_wide;
set source_wide;
run;

%MAKEWIDE(data          = cohort_fp_tpt,
          out           = date_wide,
          id            = ikn,
          var           = date);

data date_wide;
set date_wide;
run;

%MAKEWIDE(data          = cohort_fp_tpt,
          out           = dxcode_wide,
          id            = ikn,
          var           = dxcode);

data dxcode_wide;
set dxcode_wide;
run;

%MAKEWIDE(data          = cohort_fp_tpt,
          out           = dx10code1_wide,
          id            = ikn,
          var           = dx10code1_);

data dx10code1_wide;
set dx10code1_wide;
run;

%MAKEWIDE(data          = cohort_fp_tpt,
          out           = dx10code2_wide,
```

```
            id          = ikn,
            var         = dx10code2_);

data dx10code2_wide;
set dx10code2_wide;
run;

%MAKEWIDE(data          = cohort_fp_tpt,
            out         = dx10code3_wide,
            id          = ikn,
            var         = dx10code3_);

data dx10code3_wide;
set dx10code3_wide;
run;


/*now merge together*/
data tpt_wide_fp;
merge source_wide
date_wide
dxcode_wide
dx10code1_wide
dx10code2_wide
dx10code3_wide;
by ikn;
run;



/*Create 6 variables to describe whether individual falls into high_specific
mod_specific or low_specific codes

/*1 - high_sp*/

data t1;
set tpt_wide_fp;
array dxc[285] $ dxcode1-dxcode285;
array dx10c1[285] $dx10code1_1-dx10code1_285;
array dx10c2[285] $dx10code2_1-dx10code2_285;
array dx10c3[285] $dx10code3_1-dx10code3_285;
h_sp=0;
do i=1 to 285;
      if          dxc[i] in: ('002')      then h_sp=1;
      else if dx10c1[i] in: ('A01')       then h_sp=1;
      else if dx10c2[i] in: ('A01')       then h_sp=1;
      else if dx10c3[i] in: ('A01')       then h_sp=1;
      end;
      run;

proc freq data=t1;
tables h_sp;
run; /*1646 meet this criteria*/


/*2 - moderate specificity (m_sp)*/
```

```
data t1;
set t1;
array dxc[285] $ dxcode1-dxcode285;
array dx10c1[285] $dx10code1_1-dx10code1_285;
array dx10c2[285] $dx10code2_1-dx10code2_285;
array dx10c3[285] $dx10code3_1-dx10code3_285;
m_sp=0;
do i=1 to 285;
      if          dxc[i] in:('009')       then m_sp=1;
      else if dx10c1[i] in: ('A029')      then m_sp=1;
      else if dx10c2[i] in: ('A029')      then m_sp=1;
      else if dx10c3[i] in: ('A029')      then m_sp=1;
      end;
      run;

proc freq data=t1;
tables m_sp;
run;


/*3 - low specificity (l_sp)*/

data t1;
set t1;
array dxc[285] $ dxcode1-dxcode285;
array dx10c1[285] $dx10code1_1-dx10code1_285;
array dx10c2[285] $dx10code2_1-dx10code2_285;
array dx10c3[285] $dx10code3_1-dx10code3_285;
l_sp=0;
do i=1 to 285;
      if    dxc[i] in: ('003' '005' '136' '784' '787') then l_sp=1;
      else if dx10c1[i] in: ('A021' 'A049' 'A059' 'A09' 'A499')then l_sp=1;
      else if dx10c2[i] in: ('A021' 'A049' 'A059' 'A09' 'A499')then l_sp=1;
      else if dx10c3[i] in: ('A021' 'A049' 'A059' 'A09' 'A499')then l_sp=1;
      end;
      run;

proc freq data=t1;
tables l_sp;
run;


/*code distribution for low spec FP*/

data t1;
set t1;
array dxc[285] $ dxcode1-dxcode285;
c_002=0;
do i=1 to 285;
      if    dxc[i] in: ('002') then c_002=1;
      end;
      run;

data t1;
set t1;
array dx10c1[285] $dx10code1_1-dx10code1_285;
array dx10c2[285] $dx10code2_1-dx10code2_285;
```

```
array dx10c3[285] $dx10code3_1-dx10code3_285;
c_A01=0;
do i=1 to 285;
      if dx10c1[i] in: ('A01')            then c_A01=1;
      else if dx10c2[i] in: ('A01')       then c_A01=1;
      else if dx10c3[i] in: ('A01')       then c_A01=1;
      end;
      run;

data t1;
set t1;
array dx10c1[285] $dx10code1_1-dx10code1_285;
array dx10c2[285] $dx10code2_1-dx10code2_285;
array dx10c3[285] $dx10code3_1-dx10code3_285;
codeA02=0;
do i=1 to 285;
      if dx10c1[i]            in: ('A021' 'A029')     then codeA02=1;
      else if dx10c2[i]       in: ('A021' 'A029')     then codeA02=1;
      else if dx10c3[i]       in: ('A021' 'A029')     then codeA02=1;
      end;
      run;

data t1;
set t1;
array dxc[285] $ dxcode1-dxcode285;
code009=0;
do i=1 to 285;
      if    dxc[i]            in: ('009') then code009=1;
      end;
      run;

data t1;
set t1;
array dxc[285] $ dxcode1-dxcode285;
code003=0;
do i=1 to 285;
      if    dxc[i]            in: ('003') then code003=1;
      end;
      run;

data t1;
set t1;
array dxc[285] $ dxcode1-dxcode285;
code136=0;
do i=1 to 285;
      if    dxc[i]            in: ('136') then code136=1;
      end;
      run;

data t1;
set t1;
array dxc[285] $ dxcode1-dxcode285;
code787=0;
do i=1 to 285;
      if    dxc[i]            in: ('787') then code787=1;
      end;
      run;
```

```
proc freq data=t1;
tables c_002 c_A01 codeA02 code009 code003 code136 code787;
run;

data t1;
set t1;
array s[285] $ source1-source285;
hosp=0;
do i=1 to 285;
      if    s[i] in: ('DAD') then hosp=1;
      end;
      run;

data t1;
set t1;
array s[285] $ source1-source285;
ed=0;
do i=1 to 285;
      if    s[i] in: ('NACRS') then ed=1;
      end;
      run;

data t1;
set t1;
array s[285] $ source1-source285;
pc=0;
do i=1 to 285;
      if    s[i] in: ('OHIP') then pc=1;
      end;
      run;

data t1;
set t1;
array s[285] $ source1-source285;
sds=0;
do i=1 to 285;
      if    s[i] in: ('SDS') then sds=1;
      end;
      run;

proc freq data=t1;
tables hosp ed pc sds;
run;


/*now merge with episodeperiod_mal to get datediffo and count summaries*/

data epi_tpt (keep=ikn datediffo count);
set episodeperiod_tpt;
run;

proc sort data=epi_tpt;
by ikn;
run;

data t2_summary;
```

```
merge epi_tpt (in=period) t1(in=typ);
by ikn;
if period and typ;
run;

proc means mean median min max data=t2_summary;
var datediffo count;
run;


/*now merge back in with original cohort to describe characteristics of TPs*/

/*create data set from original cohort with 1 obs per patient based on first
health encounter*/
proc sort data=cohort;
by ikn first_admin_code_date;
run;

data uniqueikn;
set cohort;
if first.ikn then count=0;
count + 1;
if last.ikn then output;
by ikn;
run;

data t3 (keep=ikn cohort_entry age_cohort_entry sex cic_immigrant cic_fsrce
admin_code_in_lookback);
set uniqueikn;
run;

data t4;
merge t3 (in=cohort) t2_summary(in=tpt);
by ikn;
if cohort and tpt;
run;


data t4;
set t4;
agegp=.;
if    age_cohort_entry < 18   then agegp=1;
else if age_cohort_entry ge 18 and age_cohort_entry le 64   then agegp=2;
else if age_cohort_entry ge 65 then agegp=3;
run;

proc univariate data=t4;
var age_cohort_entry;
run;

proc freq data=t4;
tables agegp sex cic_immigrant cic_fsrce admin_code_in_lookback;
run;

data t4;
set t4;
array dx10c1[285] $dx10code1_1-dx10code1_285;
```

```
array dx10c2[285] $dx10code2_1-dx10code2_285;
array dx10c3[285] $dx10code3_1-dx10code3_285;
c_A021=0;
do i=1 to 285;
      if dx10c1[i] in: ('A021')          then c_A021=1;
      else if dx10c2[i] in: ('A021')     then c_A021=1;
      else if dx10c3[i] in: ('A021')     then c_A021=1;
      end;
      run;

data t4;
set t4;
array dx10c1[285] $dx10code1_1-dx10code1_285;
array dx10c2[285] $dx10code2_1-dx10code2_285;
array dx10c3[285] $dx10code3_1-dx10code3_285;
c_A029=0;
do i=1 to 285;
      if dx10c1[i] in: ('A029')          then c_A029=1;
      else if dx10c2[i] in: ('A029')     then c_A029=1;
      else if dx10c3[i] in: ('A029')     then c_A029=1;
      end;
      run;

data t4;
set t4;
array dx10c1[285] $dx10code1_1-dx10code1_285;
array dx10c2[285] $dx10code2_1-dx10code2_285;
array dx10c3[285] $dx10code3_1-dx10code3_285;
c_A049=0;
do i=1 to 285;
      if dx10c1[i] in: ('A049')          then c_A049=1;
      else if dx10c2[i] in: ('A049')     then c_A049=1;
      else if dx10c3[i] in: ('A049')     then c_A049=1;
      end;
      run;

data t4;
set t4;
array dx10c1[285] $dx10code1_1-dx10code1_285;
array dx10c2[285] $dx10code2_1-dx10code2_285;
array dx10c3[285] $dx10code3_1-dx10code3_285;
c_A059=0;
do i=1 to 285;
      if dx10c1[i] in: ('A059')          then c_A059=1;
      else if dx10c2[i] in: ('A059')     then c_A059=1;
      else if dx10c3[i] in: ('A059')     then c_A059=1;
      end;
      run;

data t4;
set t4;
array dx10c1[285] $dx10code1_1-dx10code1_285;
array dx10c2[285] $dx10code2_1-dx10code2_285;
array dx10c3[285] $dx10code3_1-dx10code3_285;
c_A09=0;
do i=1 to 285;
      if dx10c1[i] in: ('A09')           then c_A09=1;
```

```
      else if dx10c2[i] in: ('A09')       then c_A09=1;
      else if dx10c3[i] in: ('A09')       then c_A09=1;
      end;
      run;

data t4;
set t4;
array dx10c1[285] $dx10code1_1-dx10code1_285;
array dx10c2[285] $dx10code2_1-dx10code2_285;
array dx10c3[285] $dx10code3_1-dx10code3_285;
c_A499=0;
do i=1 to 285;
      if dx10c1[i] in: ('A499')           then c_A499=1;
      else if dx10c2[i] in: ('A499')      then c_A499=1;
      else if dx10c3[i] in: ('A499')      then c_A499=1;
      end;
      run;

proc freq data=t4;
tables c_A021 c_A01 c_A029 c_A049 c_A059 c_A09 c_A499;
run;

data t4;
set t4;
if c_A049=1 or c_A059=1 then c_A04959=1;
run;

proc freq data=t4;
tables c_A04959;
run;


/*save as permanent dataset*/

data DATASETS.final_tpt_fp_02may2017;
set t4;
run;


/*forgot to run code freq - July 6 2017*/

data t;
set DATASETS.final_tpt_fp_02may2017;
run;

data t (where=(h_sp=1));
set t;
run;

proc freq data=t;
tables c_002 code009 code003 code136 code787 c_A01 codeA02 c_A04959 c_A09
c_A499;
run;



/**HIGH SPEC ANALYSIS**/
```

```
data t6 (where=(h_sp=1));
set t4;
run;

proc freq data=t6;
tables agegp sex cic_immigrant cic_fsrce admin_code_in_lookback;
run;

proc freq data=t6;
tables c_002 c_A01 c_002*c_A01 hosp ed pc sds;
run;

proc means mean median min max data=t6;
var datediffo count age_cohort_entry;
run;


/*describe codes of those hosp*/
data t4_hosp (where=(hosp=1));
set t6;
run;

proc freq data=t4_hosp;
tables c_A021 c_A01 c_A029 c_A049 c_A059 c_A09 c_A499;
run;

proc means mean median min max data=t4_hosp;
var age_cohort_entry;
run;

proc freq data=t4_hosp;
tables agegp sex cic_immigrant cic_fsrce admin_code_in_lookback;
run;



/*look at high spec FP who were hosp*/

data fp_hosp;
merge ha_hosp t4_hosp m4_hosp;
by ikn;
run;

data fp_hosp;
set fp_hosp;
fp_hosp=1;
run;

data fp_hosp (keep=ikn fp_hosp);
set fp_hosp;
run;

data fp_hosp2;
merge fp_hosp (in=fp) cohort (in=cohort);
```

```
by ikn;
if fp and cohort;
run;

data fp_hosp3 (keep=ikn fp_hosp source dxcode dx10code_1 dx10code_2
dx10code_3 date);
set fp_hosp2;
run;

data fp_hosp3;
set fp_hosp3;
hosp2=0;
if source in ('DAD') and ((dx10code_1 in: ('B15' 'B5' 'A01')) or (dx10code_2
in: ('B15' 'B5' 'A01')) or (dx10code_3 in: ('B15' 'B5' 'A01'))) then hosp2=1;
run;

data fp_hosp4;
set fp_hosp3;
where hosp2=1;
run;



/**** TRUE POSITIVES ****/


/*What codes are used for TRUE positives (in both iPHIS and ADMIN)?*/

data cohort_tp (where=(iphis=1 and first_admin_code_date ne .));
set cohort;
run;

/*NOTE: this is not the best way to define (ne . in admin) as several have
health admin data but not related to the disease in question -
that is they do not have one of the specified codes and the date is not close
to their episode date in iPHIS*/

data cohort_tp;
set cohort_tp;
if    iphis_disease in ("HEPATITIS A") then diseasegp=1;
else if iphis_disease in ("MALARIA") then diseasegp=2;
else if iphis_disease in ("PARATYPHOID FEVER" "TYPHOID FEVER") then
diseasegp=3;
run;

proc freq data=cohort_tp;
tables diseasegp;
run;

proc sort data=cohort_tp;
by ikn first_admin_code_date;
run;

data uniqueikn;
set cohort_tp;
if first.ikn then count=0;
count + 1;
```

```
if last.ikn then output;
by ikn;
run;


/*********HEPA**********/


data cohort_tp_hep2;
set cohort_tp;
hep=0;
      if dxcode     in: ('070' '009' '079' '136' '787')     then hep=1;
      else if dx10code_1 in: ('B15' 'B19' 'A09' 'A083' 'A084' 'A085') then
hep=1;
      else if dx10code_2 in: ('B15' 'B19' 'A09' 'A083' 'A084' 'A085') then
hep=1;
      else if dx10code_3 in: ('B15' 'B19' 'A09' 'A083' 'A084' 'A085') then
hep=1;
      run;

proc freq data=cohort_tp_hep2;
tables hep;
run;

data cohort_tp_hep2 (where=(diseasegp=1 and hep=1));
set cohort_tp_hep2;
run;

proc freq data=cohort_tp_hep2;
tables dxcode dx10code:;
run;

/*how many unique individuals?*/

proc sort data=cohort_tp_hep2;
by ikn first_admin_code_date;
run;

data uniqueikn_hep;
set cohort_tp_hep2;
if first.ikn then count=0;
count + 1;
if last.ikn then output;
by ikn;
run;

/*Look at time between first and last date in individuals with >1 episode*/

data cohort_tp_hep2;
set cohort_tp_hep2;
datediffo=date-first_admin_code_date;
run;

proc sort data=cohort_tp_hep2;
by ikn first_admin_code_date date;
run;
```

```
data episodeperiod_hep2;
set cohort_tp_hep2;
if first.ikn then count=0;
count + 1;
if last.ikn then output;
by ikn;
run;

proc means mean median min max data=episodeperiod_hep2;
var datediffo count;
run;


/*Look at time between iPHIS episode date (onset) and health encounter dates
in admin data*/

data cohort_tp_hep2;
set cohort_tp_hep2;
datediffiphis=date - iphis_accurateepisodedate;
run;

proc freq data=cohort_tp_hep2;
tables datediffiphis;
run;

proc univariate data=cohort_tp_hep2;
var datediffiphis;
histogram;
run;

proc means mean min max data=cohort_tp_hep2;
var datediffiphis;
run;

data cohort_tp_hep2;
set cohort_tp_hep2;
if    datediffiphis < -7 then iphisdategp=1;
else if datediffiphis ge -7 and datediffiphis le 30 then iphisdategp=2;
else if datediffiphis > 30 then iphisdategp=3;
run;

proc freq data=cohort_tp_hep2;
tables iphisdategp;
run;


proc sort data=cohort_tp_hep2;
by ikn datediffiphis;
run;


/*what is distribution like for most specific OHIP (070) code?*/

proc freq data=cohort_tp_hep2;
where dxcode in: ('070');
tables datediffiphis;
run;
```

```
proc freq data=cohort_tp_hep2;
where dx10code_1 in: ('B15');
tables datediffiphis;
run;

proc univariate data=cohort_tp_hep2;
where dxcode in: ('070');
var datediffiphis;
histogram;
run;

proc univariate data=cohort_tp_hep2;
where dx10code_1 in: ('B15') or dx10code_2 in: ('B15') or dx10code_3 in:
('B15') ;
var datediffiphis;
histogram;
run;


/*based on looking at FN, -40 to 120 seems an appropriate range*/

data cohort_tp_hep2;
set cohort_tp_hep2;
if    datediffiphis < -40 then iphisdategp2=1;
else if datediffiphis ge -40 and datediffiphis le 120 then iphisdategp2=2;
else if datediffiphis > 120 then iphisdategp2=3;
run;

proc freq data=cohort_tp_hep2;
tables iphisdategp2;
run;

proc sort data=cohort_tp_hep2;
by iphisdategp2;
run;

proc freq data=cohort_tp_hep2;
by iphisdategp2;
tables dxcode;
run;

proc freq data=cohort_tp_hep2;
by iphisdategp2;
tables dx10code_1;
run;



/*Apply rule:  IF codes 787 or 079 or 009 or A099 AND iphisdategp2 = 1 or 3,
exclude*/

data cohort_tp_hep3;
set cohort_tp_hep2;
fn=0;
if    dxcode in: ('787' '079' '009') and (iphisdategp2 ne 2)      then fn=1;
else if dx10code_1 in: ('A099') and (iphisdategp2 ne 2)           then fn=1;
```

```
run;

proc freq data=cohort_tp_hep3;
tables fn;
run; /*35 obs - now 45*/

proc sort data=cohort_tp_hep3;
by fn ikn;
run;

data cohort_tp_hep4 (where=(fn ne 1));
set cohort_tp_hep3;
run; /*now 228 observations down from 263 - now 269 down from 314*/

/*how many unique individuals?*/

proc sort data=cohort_tp_hep4;
by ikn first_admin_code_date;
run;

data uniqueikn_hep;
set cohort_tp_hep4;
if first.ikn then count=0;
count + 1;
if last.ikn then output;
by ikn;
run;



/*re-run descriptive excluding FN*/
proc freq data=cohort_tp_hep4;
tables dxcode dx10code:;
run;

proc sort data=cohort_tp_hep4;
by ikn first_admin_code_date date;
run;

data episodeperiod_hep4;
set cohort_tp_hep4;
if first.ikn then count=0;
count + 1;
if last.ikn then output;
by ikn;
run;

proc sort data=episodeperiod_hep4;
by datediffo;
run;

proc means mean median min max data=episodeperiod_hep4;
var datediffo count;
run;

proc univariate data=episodeperiod_hep4;
var count;
```

```
histogram;
run;


/** BY PATIENT ANALYSIS **/


/*Now collapse to look at constellation of codes + VENN diagram (still need
to exclude some episodes but leave in for now)*/

/*need to flatten only one row per ikn/individual level*/


data cohort_tp_hep4 (rename=(dx10code_1=dx10code1_ dx10code_2=dx10code2_
dx10code_3=dx10code3_));
set cohort_tp_hep4;
run;

%MAKEWIDE(data          = cohort_tp_hep4,
           out          = source_wide,
           id           = ikn,
           var          = source);

data source_wide;
set source_wide;
run;

%MAKEWIDE(data          = cohort_tp_hep4,
           out          = date_wide,
           id           = ikn,
           var          = date);

data date_wide;
set date_wide;
run;

%MAKEWIDE(data          = cohort_tp_hep4,
           out          = dxcode_wide,
           id           = ikn,
           var          = dxcode);

data dxcode_wide;
set dxcode_wide;
run;

%MAKEWIDE(data          = cohort_tp_hep4,
           out          = dx10code1_wide,
           id           = ikn,
           var          = dx10code1_);

data dx10code1_wide;
set dx10code1_wide;
run;

%MAKEWIDE(data          = cohort_tp_hep4,
           out          = dx10code2_wide,
           id           = ikn,
```

```
           var          = dx10code2_);

data dx10code2_wide;
set dx10code2_wide;
run;

%MAKEWIDE(data          = cohort_tp_hep4,
           out          = dx10code3_wide,
           id           = ikn,
           var          = dx10code3_);

data dx10code3_wide;
set dx10code3_wide;
run;


/*now merge together*/
data hepa_wide2;
merge source_wide
date_wide
dxcode_wide
dx10code1_wide
dx10code2_wide
dx10code3_wide;
by ikn;
run;


/*Look at constellation*/

/*high*/
data ha1;
set hepa_wide2;
array dxc[15] $ dxcode1-dxcode15;
array dx10c1[15] $dx10code1_1-dx10code1_15;
array dx10c2[15] $dx10code2_1-dx10code2_15;
array dx10c3[15] $dx10code3_1-dx10code3_15;
h_sp=0;
do i=1 to 15;
      if          dxc[i] in:('070')              then h_sp=1;
      else if dx10c1[i] in: ('B15')        then h_sp=1;
      else if dx10c2[i] in: ('B15')        then h_sp=1;
      else if dx10c3[i] in: ('B15')        then h_sp=1;
      end;
      run;

proc freq data=ha1;
tables h_sp;
run; /*35/43 meet this criteria - now 41/48*/


/*moderate*/
data ha1;
set ha1;
array dxc[15] $ dxcode1-dxcode15;
array dx10c1[15] $dx10code1_1-dx10code1_15;
array dx10c2[15] $dx10code2_1-dx10code2_15;
```

```
array dx10c3[15] $dx10code3_1-dx10code3_15;
m_sp=0;
do i=1 to 15;
      if          dxc[i] in:('009')              then m_sp=1;
      else if dx10c1[i] in: ('B19')        then m_sp=1;
      else if dx10c2[i] in: ('B19')        then m_sp=1;
      else if dx10c3[i] in: ('B19')        then m_sp=1;
      end;
      run;

proc freq data=ha1;
tables m_sp;
run;


/*low*/
data ha1;
set ha1;
array dxc[15] $ dxcode1-dxcode15;
array dx10c1[15] $dx10code1_1-dx10code1_15;
array dx10c2[15] $dx10code2_1-dx10code2_15;
array dx10c3[15] $dx10code3_1-dx10code3_15;
l_sp=0;
do i=1 to 15;
      if          dxc[i]            in: ('079' '136' '787')
      then l_sp=1;
      else if dx10c1[i]       in: ('A09' 'A083' 'A084' 'A085')    then
l_sp=1;
      else if dx10c2[i]       in: ('A09' 'A083' 'A084' 'A085')    then
l_sp=1;
      else if dx10c3[i]       in: ('A09' 'A083' 'A084' 'A085')    then
l_sp=1;
      end;
      run;

proc freq data=ha1;
tables l_sp;
run;

/*h or m*/

data ha1;
set ha1;
hm_sp=0;
if h_sp=1 or m_sp=1 then hm_sp=1;
run;

/*h or m or l*/

data ha1;
set ha1;
hml_sp=0;
if h_sp=1 or m_sp=1 or l_sp=1 then hml_sp=1;
run;

/*only high*/
```

```
data ha1;
set ha1;
onlyh_sp=0;
if h_sp=1 and m_sp=0 and l_sp=0 then onlyh_sp=1;
run;

/*only low*/

data ha1;
set ha1;
onlyl_sp=0;
if h_sp=0 and m_sp=0 and l_sp=1 then onlyl_sp=1;
run;

/*all three*/

data ha1;
set ha1;
all_sp=0;
if h_sp=1 and m_sp=1 and l_sp=1 then all_sp=1;
run;

proc freq data=ha1;
tables hm_sp hml_sp onlyh_sp onlyl_sp all_sp;
run;


/*H and M, no L*/

data ha1;
set ha1;
ab_sp=0;
if h_sp=1 and m_sp=1 and l_sp=0 then ab_sp=1;
run;

/*H and L, no M*/

data ha1;
set ha1;
ac_sp=0;
if h_sp=1 and m_sp=0 and l_sp=1 then ac_sp=1;
run;

/*M and L, no H*/

data ha1;
set ha1;
bc_sp=0;
if h_sp=0 and m_sp=1 and l_sp=1 then bc_sp=1;
run;

proc freq data=ha1;
tables ab_sp ac_sp bc_sp;
run;

/*only M*/
```

```
data ha1;
set ha1;
b_sp=0;
if h_sp=0 and m_sp=1 and l_sp=0 then b_sp=1;
run;

proc freq data=ha1;
tables b_sp;
run;



/*Describe codes and sources*/

data ha4;
set ha1;
array dxc[15] $ dxcode1-dxcode15;
array dx10c1[15] $dx10code1_1-dx10code1_15;
array dx10c2[15] $dx10code2_1-dx10code2_15;
array dx10c3[15] $dx10code3_1-dx10code3_15;
code070=0;
do i=1 to 15;
      if          dxc[i] in:('070')             then code070=1;
      end;
      run;

data ha4;
set ha4;
array dxc[15] $ dxcode1-dxcode15;
array dx10c1[15] $dx10code1_1-dx10code1_15;
array dx10c2[15] $dx10code2_1-dx10code2_15;
array dx10c3[15] $dx10code3_1-dx10code3_15;
codeb15=0;
do i=1 to 15;
      if dx10c1[i] in: ('B15')            then codeb15=1;
      else if dx10c2[i] in: ('B15')       then codeb15=1;
      else if dx10c3[i] in: ('B15')       then codeb15=1;
      end;
      run;

proc freq data=ha4;
tables code070 codeb15;
run;

data ha4;
set ha4;
array dxc[15] $ dxcode1-dxcode15;
array dx10c1[15] $dx10code1_1-dx10code1_15;
array dx10c2[15] $dx10code2_1-dx10code2_15;
array dx10c3[15] $dx10code3_1-dx10code3_15;
code009=0;
do i=1 to 15;
      if          dxc[i] in:('009')             then code009=1;
      end;
      run;

data ha4;
```

```
set ha4;
array dxc[15] $ dxcode1-dxcode15;
array dx10c1[15] $dx10code1_1-dx10code1_15;
array dx10c2[15] $dx10code2_1-dx10code2_15;
array dx10c3[15] $dx10code3_1-dx10code3_15;
code079=0;
do i=1 to 15;
      if          dxc[i] in:('079')             then code079=1;
      end;
      run;

data ha4;
set ha4;
array dxc[15] $ dxcode1-dxcode15;
array dx10c1[15] $dx10code1_1-dx10code1_15;
array dx10c2[15] $dx10code2_1-dx10code2_15;
array dx10c3[15] $dx10code3_1-dx10code3_15;
code787=0;
do i=1 to 15;
      if          dxc[i] in:('787')             then code787=1;
      end;
      run;

data ha4;
set ha4;
array dxc[15] $ dxcode1-dxcode15;
array dx10c1[15] $dx10code1_1-dx10code1_15;
array dx10c2[15] $dx10code2_1-dx10code2_15;
array dx10c3[15] $dx10code3_1-dx10code3_15;
codeb19=0;
do i=1 to 15;
      if dx10c1[i] in: ('B19')            then codeb19=1;
      else if dx10c2[i] in: ('B19')       then codeb19=1;
      else if dx10c3[i] in: ('B19')       then codeb19=1;
      end;
      run;

data ha4;
set ha4;
array dxc[15] $ dxcode1-dxcode15;
array dx10c1[15] $dx10code1_1-dx10code1_15;
array dx10c2[15] $dx10code2_1-dx10code2_15;
array dx10c3[15] $dx10code3_1-dx10code3_15;
codea09=0;
do i=1 to 15;
      if dx10c1[i] in: ('A09')            then codea09=1;
      else if dx10c2[i] in: ('A09')       then codea09=1;
      else if dx10c3[i] in: ('A09')       then codea09=1;
      end;
      run;

data ha4;
set ha4;
array dxc[15] $ dxcode1-dxcode15;
array dx10c1[15] $dx10code1_1-dx10code1_15;
array dx10c2[15] $dx10code2_1-dx10code2_15;
array dx10c3[15] $dx10code3_1-dx10code3_15;
```

```
codea08=0;
do i=1 to 15;
      if dx10c1[i] in: ('A083' 'A084' 'A085')         then codea08=1;
      else if dx10c2[i] in: ('A083' 'A084' 'A085')    then codea08=1;
      else if dx10c3[i] in: ('A083' 'A084' 'A085')    then codea08=1;
      end;
      run;

proc freq data=ha4;
tables code009 code079 code787 codeb19 codea09 codea08;
run;


data ha4;
set ha4;
a=0;
if code070=1 and code787=0 and codeb15=0 then a=1;
run;

data ha4;
set ha4;
b=0;
if code070=0 and code787=1 and codeb15=0 then b=1;
run;

data ha4;
set ha4;
c=0;
if code070=0 and code787=0 and codeb15=1 then c=1;
run;

data ha4;
set ha4;
ab=0;
if code070=1 and code787=1 and codeb15=0 then ab=1;
run;

data ha4;
set ha4;
ac=0;
if code070=1 and code787=0 and codeb15=1 then ac=1;
run;

data ha4;
set ha4;
bc=0;
if code070=0 and code787=1 and codeb15=1 then bc=1;
run;

data ha4;
set ha4;
abc=0;
if code070=1 and code787=1 and codeb15=1 then abc=1;
run;

proc freq data=ha4;
tables a b c ab ac bc abc;
```

```
run;



data ha4;
set ha4;
array s[15] $ source1-source15;
hosp=0;
do i=1 to 15;
	if	s[i] in: ('DAD') then hosp=1;
	end;
	run;

data ha4;
set ha4;
array s[15] $ source1-source15;
ed=0;
do i=1 to 15;
	if	s[i] in: ('NACRS') then ed=1;
	end;
	run;

data ha4;
set ha4;
array s[15] $ source1-source15;
pc=0;
do i=1 to 15;
	if	s[i] in: ('OHIP') then pc=1;
	end;
	run;

data ha4;
set ha4;
array s[15] $ source1-source15;
sds=0;
do i=1 to 15;
	if	s[i] in: ('SDS') then sds=1;
	end;
	run;

proc freq data=ha4;
tables hosp ed pc sds;
run;


/*now merge with datediffo and count to describe TP*/

data epi_hep_tp (keep=ikn datediffo count);
set episodeperiod_hep4;
run;

proc sort data=epi_hep_tp;
by ikn;
run;

data ha4_summary;
```

```
merge ha4(in=hep) epi_hep_tp (in=period);
by ikn;
if hep and period;
run;

proc means mean median min max data=ha4_summary;
var datediffo count;
run;


/*now merge back with original cohort to describe all TP*/

/*create data set from original cohort with 1 obs per patient based on first
health encounter*/
proc sort data=cohort;
by ikn first_admin_code_date;
run;

data uniqueikn;
set cohort;
if first.ikn then count=0;
count + 1;
if last.ikn then output;
by ikn;
run;

data h (keep=ikn cohort_entry age_cohort_entry sex cic_immigrant cic_fsrce
admin_code_in_lookback);
set uniqueikn;
run;

data h2;
merge h (in=cohort) ha4_summary (in=hep);
by ikn;
if cohort and hep;
run;


data h2;
set h2;
agegp=.;
if    age_cohort_entry < 18   then agegp=1;
else if age_cohort_entry ge 18 and age_cohort_entry le 64   then agegp=2;
else if age_cohort_entry ge 65 then agegp=3;
run;

proc univariate data=h2;
var age_cohort_entry;
run;


data h2;
set h2;
length region $20;
if    cic_fsrce in ('Asia and Pacific') then region='Asia and Pacific';
else if cic_fsrce ne (' ') then region='Other';
run;
```

```
proc freq data=h2;
tables region*cic_fsrce;
run;


proc freq data=h2;
tables agegp sex cic_immigrant region admin_code_in_lookback;
run;

proc freq data=h2;
tables code070 codeb15 code009 code079 code787 codeb19 codea09 codea08;
run;

proc means median min max data=h2;
var age_cohort_entry datediffo count;
run;


/*now save as a permanent dataset*/
/*can filter h_sp=1 to do analysis for high spec*/

data DATASETS.final_hepa_tp_02may2017;
set h2;
run;



/******HIGH SPECIFICITY TP ANALYSIS*******/

/*Merge high spec back with cohort to describe TP*/

data h2;
set DATASETS.final_hepa_tp_02may2017;
run;

data ha5 (where=(h_sp=1));
set h2;
run;

proc freq data=ha5;
tables agegp sex cic_immigrant region admin_code_in_lookback;
run;

proc means median min max data=ha5;
var age_cohort_entry;
run;

proc freq data=ha5;
tables code070 code009 code079 code787 codeb15 codeb19 codea09 codea08;
run;

proc freq data=ha5;
tables code070*codeb15;
run;

proc freq data=ha5;
```

```
tables hosp ed pc sds;
run;


/*Go back to describe high spec TPs at the observation level, not patient
level*/

data ha6 (keep=ikn h_sp);
set ha5;
run;

data ha7;
merge ha6 (in=hephsp) cohort_tp_hep4 (in=cohort);
by ikn;
if hephsp and cohort;
run;

/*how many unique individuals?*/

proc sort data=ha7;
by ikn first_admin_code_date;
run;

data uniqueikn_hep_ha7;
set ha7;
if first.ikn then count=0;
count + 1;
if last.ikn then output;
by ikn;
run; /*41 unique IKNs*/

/*describe code distribution*/
proc freq data=ha7;
tables dxcode dx10code1_ dx10code2_ dx10code3_;
run;


/*Look at time between first and last date in individuals with >1 episode*/

proc sort data=ha7;
by ikn first_admin_code_date date;
run;

data episodeperiod_hep2_ha7;
set ha7;
if first.ikn then count=0;
count + 1;
if last.ikn then output;
by ikn;
run;

proc means mean median min max data=episodeperiod_hep2_ha7;
var count datediffo;
run;
```

```
/*Look at time between iPHIS episode date (onset) and health encounter dates
in admin data*/

proc freq data=ha7;
tables datediffiphis;
run;

proc univariate data=ha7;
var datediffiphis;
histogram;
run;

proc means mean min max data=ha7;
var datediffiphis;
run;

proc freq data=ha7;
tables iphisdategp2;
run;


/**********MALARIA**********/


data cohort_tp_mal2;
set cohort_tp;
mal=0;
      if           dxcode     in: ('062' '136' '781' '784' '787')  then mal=1;
      else if dx10code_1 in: ('B5' 'P373' 'P374' 'B64')           then mal=1;
      else if dx10code_2 in: ('B5' 'P373' 'P374' 'B64')           then mal=1;
      else if dx10code_3 in: ('B5' 'P373' 'P374' 'B64')           then mal=1;
      run;

proc freq data=cohort_tp_mal2;
tables mal;
run;

data cohort_tp_mal2 (where=(diseasegp=2 and mal=1));
set cohort_tp_mal2;
run; /*428 observations - now 1142*/

proc freq data=cohort_tp_mal2;
tables dxcode dx10code:;
run;

/*how many unique individuals?*/

proc sort data=cohort_tp_mal2;
by ikn first_admin_code_date;
run;

data uniqueikn_mal;
set cohort_tp_mal2;
if first.ikn then count=0;
count + 1;
if last.ikn then output;
by ikn;
```

```
run;


/*Look at time between first and last date in individuals with >1 episode*/

data cohort_tp_mal2;
set cohort_tp_mal2;
datediffo=date-first_admin_code_date;
run;

proc sort data=cohort_tp_mal2;
by ikn first_admin_code_date date;
run;

data episodeperiod_mal2;
set cohort_tp_mal2;
if first.ikn then count=0;
count + 1;
if last.ikn then output;
by ikn;
run;

proc sort data=episodeperiod_mal2;
by datediffo;
run;

proc means mean min max median data=episodeperiod_mal2;
var datediffo count;
run;



/*Look at time between iPHIS episode date (onset) and health encounter dates
in admin data*/

data cohort_tp_mal2;
set cohort_tp_mal2;
datediffiphis=date - iphis_accurateepisodedate;
run;

proc freq data=cohort_tp_mal2;
tables datediffiphis;
run;

proc univariate data=cohort_tp_mal2;
var datediffiphis;
histogram;
run;

proc means mean min max median data=cohort_tp_mal2;
var datediffiphis;
run;

data cohort_tp_mal2;
set cohort_tp_mal2;
if    datediffiphis < -7 then iphisdategp=1;
else if datediffiphis ge -7 and datediffiphis le 30 then iphisdategp=2;
```

```
else if datediffiphis > 30 then iphisdategp=3;
run;

proc freq data=cohort_tp_mal2;
tables iphisdategp;
run;

proc sort data=cohort_tp_mal2;
by iphisdategp;
run;

proc freq data=cohort_tp_mal2;
by iphisdategp;
tables dxcode;
run;

proc freq data=cohort_tp_mal2;
by iphisdategp;
tables dx10code_1;
run;


/*what is distribution like for most specific codes?*/

proc freq data=cohort_tp_mal2;
where dxcode in: ('136');
tables datediffiphis;
run;

proc freq data=cohort_tp_mal2;
where dx10code_1 in: ('B5');
tables datediffiphis;
run;


data cohort_tp_mal2;
set cohort_tp_mal2;
if          datediffiphis < -30     then iphisdategp2=1;
else if datediffiphis ge -30 and datediffiphis le 120 then iphisdategp2=2;
else if datediffiphis > 120 then iphisdategp2=3;
run;

proc freq data=cohort_tp_mal2;
tables iphisdategp2;
run;

proc sort data=cohort_tp_mal2;
by iphisdategp2;
run;

proc freq data=cohort_tp_mal2;
by iphisdategp2;
tables dxcode;
run;

proc freq data=cohort_tp_mal2;
by iphisdategp2;
```

```
tables dx10code_1;
run;


/*look for False Negatives*/
data cohort_tp_mal2_fn (keep=ikn iphis_accurateepisodedate source date ddate
dxcode dx10code1_ dx10code2_ dx10code3_ datediffiphis iphisdategp2);
set cohort_tp_mal2;
run;

proc sort data=cohort_tp_mal2_fn;
by ikn date;
run;

proc sort data=cohort_tp_mal2_fn;
by dxcode ikn date;
run;


/*Apply rule:  IF codes 781, 787 or 136 AND iphisdategp2 = 1 or 3, exclude*/

data cohort_tp_mal3;
set cohort_tp_mal2;
fn=0;
if dxcode in: ('136' '781' '787') and (iphisdategp2 ne 2)   then fn=1;
run;

proc freq data=cohort_tp_mal3;
tables fn;
run;

proc sort data=cohort_tp_mal3;
by fn ikn;
run;

data cohort_tp_mal4 (where=(fn ne 1));
set cohort_tp_mal3;
run;

/*how many unique individuals?*/

proc sort data=cohort_tp_mal4;
by ikn first_admin_code_date;
run;

data uniqueikn_mal;
set cohort_tp_mal4;
if first.ikn then count=0;
count + 1;
if last.ikn then output;
by ikn;
run; /*95 unique IKNs*/


/*re-run descriptive excluding FN*/
proc freq data=cohort_tp_mal4;
tables dxcode dx10code:;
```

```
run;

proc sort data=cohort_tp_mal4;
by ikn first_admin_code_date date;
run;

data episodeperiod_mal4;
set cohort_tp_mal4;
if first.ikn then count=0;
count + 1;
if last.ikn then output;
by ikn;
run;

proc sort data=episodeperiod_mal4;
by datediffo;
run;

proc means mean median min max data=episodeperiod_mal4;
var datediffo count;
run; /*using this dataset only gives values based on first and last visit*/



/*Now collapse to look at constellation of codes + VENN diagram (still need
to exclude some episodes but leave in for now)*/

/*need to flatten only one row per ikn/individual level*/


data cohort_tp_mal4 (rename=(dx10code_1=dx10code1_ dx10code_2=dx10code2_
dx10code_3=dx10code3_));
set cohort_tp_mal4;
run;

%MAKEWIDE(data          = cohort_tp_mal4,
          out           = source_wide,
          id            = ikn,
          var           = source);

data source_wide;
set source_wide;
run;

%MAKEWIDE(data          = cohort_tp_mal4,
          out           = date_wide,
          id            = ikn,
          var           = date);

data date_wide;
set date_wide;
run;

%MAKEWIDE(data          = cohort_tp_mal4,
          out           = dxcode_wide,
          id            = ikn,
          var           = dxcode);
```

```
data dxcode_wide;
set dxcode_wide;
run;

%MAKEWIDE(data          = cohort_tp_mal4,
           out          = dx10code1_wide,
           id           = ikn,
           var          = dx10code1_);

data dx10code1_wide;
set dx10code1_wide;
run;

%MAKEWIDE(data          = cohort_tp_mal4,
           out          = dx10code2_wide,
           id           = ikn,
           var          = dx10code2_);

data dx10code2_wide;
set dx10code2_wide;
run;

%MAKEWIDE(data          = cohort_tp_mal4,
           out          = dx10code3_wide,
           id           = ikn,
           var          = dx10code3_);

data dx10code3_wide;
set dx10code3_wide;
run;


/*now merge together*/
data mal_wide4;
merge source_wide
date_wide
dxcode_wide
dx10code1_wide
dx10code2_wide
dx10code3_wide;
by ikn;
run;


/*Look at constellation*/

data m1;
set mal_wide4;
array dxc[37] $ dxcode1-dxcode37;
array dx10c1[37] $dx10code1_1-dx10code1_37;
array dx10c2[37] $dx10code2_1-dx10code2_37;
array dx10c3[37] $dx10code3_1-dx10code3_37;
h_sp=0;
do i=1 to 37;
      if          dxc[i] in: ('062')            then h_sp=1;
      else if dx10c1[i] in: ('B5')  then h_sp=1;
```

```
      else if dx10c2[i] in: ('B5')  then h_sp=1;
      else if dx10c3[i] in: ('B5')  then h_sp=1;
      end;
      run;

proc freq data=m1;
tables h_sp;
run; /*55/68 meet this criteria - now 78/95*/


/*2 - moderate specificity (m_sp)*/

data m1;
set m1;
array dxc[37] $ dxcode1-dxcode37;
array dx10c1[37] $dx10code1_1-dx10code1_37;
array dx10c2[37] $dx10code2_1-dx10code2_37;
array dx10c3[37] $dx10code3_1-dx10code3_37;
m_sp=0;
do i=1 to 37;
      if          dxc[i] in:('062')                        then m_sp=1;
      else if dx10c1[i] in: ('P373' 'P374')     then m_sp=1;
      else if dx10c2[i] in: ('P373' 'P374')     then m_sp=1;
      else if dx10c3[i] in: ('P373' 'P374')     then m_sp=1;
      end;
      run;

proc freq data=m1;
tables m_sp;
run; /*0/68 meet this criteria*/


/*3 - low specificity (l_sp)*/

data m1;
set m1;
array dxc[37] $ dxcode1-dxcode37;
array dx10c1[37] $dx10code1_1-dx10code1_37;
array dx10c2[37] $dx10code2_1-dx10code2_37;
array dx10c3[37] $dx10code3_1-dx10code3_37;
l_sp=0;
do i=1 to 37;
      if          dxc[i]      in: ('136' '781' '784' '787') then l_sp=1;
      else if dx10c1[i]       in: ('B64')                   then l_sp=1;
      else if dx10c2[i]       in: ('B64')                   then l_sp=1;
      else if dx10c3[i]       in: ('B64')                   then l_sp=1;
      end;
      run;

proc freq data=m1;
tables l_sp;
run; /*62/68 meet this criteria - now 87*/


/*h or m*/

data m1;
```

```
set m1;
hm_sp=0;
if h_sp=1 or m_sp=1 then hm_sp=1;
run;

/*h or m or l*/

data m1;
set m1;
hml_sp=0;
if h_sp=1 or m_sp=1 or l_sp=1 then hml_sp=1;
run;

/*only high*/

data m1;
set m1;
onlyh_sp=0;
if h_sp=1 and m_sp=0 and l_sp=0 then onlyh_sp=1;
run;

/*only low*/

data m1;
set m1;
onlyl_sp=0;
if h_sp=0 and m_sp=0 and l_sp=1 then onlyl_sp=1;
run;

/*all three*/

data m1;
set m1;
all_sp=0;
if h_sp=1 and m_sp=1 and l_sp=1 then all_sp=1;
run;

proc freq data=m1;
tables hm_sp hml_sp onlyh_sp onlyl_sp all_sp;
run;


/*H and M, no L*/

data m1;
set m1;
ab_sp=0;
if h_sp=1 and m_sp=1 and l_sp=0 then ab_sp=1;
run;

/*H and L, no M*/

data m1;
set m1;
ac_sp=0;
if h_sp=1 and m_sp=0 and l_sp=1 then ac_sp=1;
run;
```

```
/*M and L, no H*/

data m1;
set m1;
bc_sp=0;
if h_sp=0 and m_sp=1 and l_sp=1 then bc_sp=1;
run;

proc freq data=m1;
tables ab_sp ac_sp bc_sp;
run;

/*only M*/

data m1;
set m1;
b_sp=0;
if h_sp=0 and m_sp=1 and l_sp=0 then b_sp=1;
run;

proc freq data=m1;
tables b_sp;
run;


/*look at specific codes and create VENN diagram*/
data m1;
set m1;
array dxc[37] $ dxcode1-dxcode37;
array dx10c1[37] $dx10code1_1-dx10code1_37;
array dx10c2[37] $dx10code2_1-dx10code2_37;
array dx10c3[37] $dx10code3_1-dx10code3_37;
codeB5=0;
do i=1 to 37;
      if dx10c1[i]            in: ('B5')  then codeB5=1;
      else if dx10c2[i]       in: ('B5')  then codeB5=1;
      else if dx10c3[i]       in: ('B5')  then codeB5=1;
      end;
      run;

data m1;
set m1;
array dxc[37] $ dxcode1-dxcode37;
array dx10c1[37] $dx10code1_1-dx10code1_37;
array dx10c2[37] $dx10code2_1-dx10code2_37;
array dx10c3[37] $dx10code3_1-dx10code3_37;
code136=0;
do i=1 to 37;
      if    dxc[i]            in: ('136') then code136=1;
      end;
      run;

data m1;
set m1;
array dxc[37] $ dxcode1-dxcode37;
array dx10c1[37] $dx10code1_1-dx10code1_37;
```

```
array dx10c2[37] $dx10code2_1-dx10code2_37;
array dx10c3[37] $dx10code3_1-dx10code3_37;
code787=0;
do i=1 to 37;
      if    dxc[i]            in: ('787') then code787=1;
      end;
      run;

data m1;
set m1;
array dxc[37] $ dxcode1-dxcode37;
array dx10c1[37] $dx10code1_1-dx10code1_37;
array dx10c2[37] $dx10code2_1-dx10code2_37;
array dx10c3[37] $dx10code3_1-dx10code3_37;
code781=0;
do i=1 to 37;
      if    dxc[i]            in: ('781') then code781=1;
      end;
      run;

proc freq data=m1;
tables codeB5 code136 code787 code781;
run;

data m1;
set m1;
array s[37] $ source1-source37;
hosp=0;
do i=1 to 37;
      if    s[i] in: ('DAD') then hosp=1;
      end;
      run;

data m1;
set m1;
array s[37] $ source1-source37;
pc=0;
do i=1 to 37;
      if    s[i] in: ('OHIP') then pc=1;
      end;
      run;

data m1;
set m1;
array s[37] $ source1-source37;
ed=0;
do i=1 to 37;
      if    s[i] in: ('NACRS') then ed=1;
      end;
      run;

data m1;
set m1;
array s[37] $ source1-source37;
sds=0;
do i=1 to 37;
      if    s[i] in: ('SDS') then sds=1;
```

```
      end;
      run;

proc freq data=m1;
tables hosp ed pc sds;
run;

data m1;
set m1;
a=0;
if codeB5=1 and code136=0 and code787=0 then a=1;
run;

data m1;
set m1;
b=0;
if codeB5=0 and code136=1 and code787=0 then b=1;
run;

data m1;
set m1;
c=0;
if codeB5=0 and code136=0 and code787=1 then c=1;
run;

data m1;
set m1;
ab=0;
if codeB5=1 and code136=1 and code787=0  then ab=1;
run;

data m1;
set m1;
ac=0;
if codeB5=1 and code136=0 and code787=1  then ac=1;
run;

data m1;
set m1;
bc=0;
if codeB5=0 and code136=1 and code787=1 then bc=1;
run;

data m1;
set m1;
abc=0;
if codeB5=1 and code136=1 and code787=1 then abc=1;
run;

proc freq data=m1;
tables a b c ab ac bc abc;
run;

data m1;
set m1;
other=0;
if a=0 and b=0 and c=0 and ab=0 and ac=0 and bc=0 and abc=0 then other=1;
```

```
run;

proc freq data=m1;
tables other;
run; /*2*/


/*Merge back with time between first and last date in individuals with >1
episode*/

data a (keep=ikn count datediffo);
set episodeperiod_mal4; /*recalculated based on excluded FN episodes*/
run;

data m2_summary;
merge m1(in=malaria) a(in=a);
by ikn;
if malaria and a;
run;

proc means mean median min max data=m2_summary;
var count datediffo;
run;


/*now merge back with original cohort to describe all TP*/

/*create data set from original cohort with 1 obs per patient based on first
health encounter*/
proc sort data=cohort;
by ikn first_admin_code_date;
run;

data uniqueikn;
set cohort;
if first.ikn then count=0;
count + 1;
if last.ikn then output;
by ikn;
run;

data m3 (keep=ikn cohort_entry age_cohort_entry sex cic_immigrant cic_fsrce
admin_code_in_lookback);
set uniqueikn;
run;

data m4;
merge m3 (in=cohort) m2_summary (in=mal);
by ikn;
if cohort and mal;
run;

data m4;
set m4;
agegp=.;
if          age_cohort_entry < 18   then agegp=1;
else if age_cohort_entry ge 18 and age_cohort_entry le 64   then agegp=2;
```

```
else if age_cohort_entry ge 65      then agegp=3;
run;

proc univariate data=m4;
var age_cohort_entry;
run;

data m4;
set m4;
length region $20;
if    cic_fsrce in ('Asia and Pacific')   then region='Asia and Pacific';
else if cic_fsrce in ('Africa and the Middle East') then region='Africa and
ME';
else if cic_fsrce ne (' ')                  then region='Other';
run;

proc freq data=m4;
tables region*cic_fsrce;
run;

proc freq data=m4;
tables agegp sex cic_immigrant region admin_code_in_lookback;
run;

proc means mean median min max data=m4;
var datediffo count age_cohort_entry;
run;


/*save as permanent dataset*/
data DATASETS.final_mal_tp_02may2017;
set m4;
run;



/* Now merge back with original cohort to describe malaria TP in depth */

/*merge with refstd*/

data r (keep=ikn);
set mal_lownohosp;
run;

proc sort data=refstd;
by ikn;
run;

data mal_lownohosp;
merge r (in=mal) refstd (in=ref);
by ikn;
if mal and ref;
run;

proc freq data=mal_lownohosp;
tables travelassociated malariachemoprophylaxiscomplet chills fever headache
interventiontype;
```

```
run;



/**** HIGH SPECIFICITY MALARIA TP ANALYSIS ****/

/*Merge high spec back with cohort to describe TP*/


data m4;
set DATASETS.final_mal_tp_02may2017;
run;


data m5 (where=(h_sp=1));
set m4;
run;


proc freq data=m5;
tables agegp sex cic_immigrant region admin_code_in_lookback;
run;

proc means median min max data=m5;
var age_cohort_entry;
run;

proc freq data=m5;
tables codeB5 code136 code787 code781;
run;

proc freq data=m5;
tables hosp ed pc sds;
run;


/*Go back to describe high spec TPs at the observation level, not patient
level*/

data m6 (keep=ikn h_sp);
set m5;
run;

data m7;
merge m6 (in=malhsp) cohort_tp_mal4 (in=cohort);
by ikn;
if malhsp and cohort;
run;

/*how many unique individuals?*/

proc sort data=m7;
by ikn first_admin_code_date;
run;

data uniqueikn_mal_m7;
```

```
set m7;
if first.ikn then count=0;
count + 1;
if last.ikn then output;
by ikn;
run;

/*describe code distribution*/
proc freq data=m7;
tables dxcode dx10code1_ dx10code2_ dx10code3_;
run;


data ha7;
set ha7;
run;

/*Look at time between first and last date in individuals with >1 episode*/

proc sort data=m7;
by ikn first_admin_code_date date;
run;

data episodeperiod_mal_m7;
set m7;
if first.ikn then count=0;
count + 1;
if last.ikn then output;
by ikn;
run;

proc means mean median min max data=episodeperiod_mal_m7;
var count datediffo;
run;


/*Look at time between iPHIS episode date (onset) and health encounter dates
in admin data*/

proc freq data=m7;
tables datediffiphis;
run;

proc means mean min max median data=m7;
var datediffiphis;
run;

proc freq data=m7;
tables iphisdategp2;
run;


/*describe codes*/

proc freq data=m5;
tables h_sp onlyh_sp l_sp all_sp;
run;
```

```
data m5;
set m5;
if codeB5=1 and code136=0 and code787=0 then a=1;
run;

proc freq data=m5;
tables a b c ab ac bc abc other;
run;



/****TYPHOID AND PARATYPHOID****/

data cohort_tp_tpt2;
set cohort_tp;
tpt=0;
      if dxcode in: ('002' '009' '003' '005' '136' '784' '787')   then tpt=1;
      else if dx10code_1 in: ('A01' 'A029' 'A021' 'A049' 'A059' 'A09' 'A499')
then tpt=1;
      else if dx10code_2 in: ('A01' 'A029' 'A021' 'A049' 'A059' 'A09' 'A499')
then tpt=1;
      else if dx10code_3 in: ('A01' 'A029' 'A021' 'A049' 'A059' 'A09' 'A499')
then tpt=1;
      run;

proc freq data=cohort_tp_tpt2;
tables tpt;
run;

data cohort_tp_tpt2 (where=(diseasegp=3 and tpt=1));
set cohort_tp_tpt2;
run;

/*need to correct for two cases with two distinct disease episodes*/

data duplicates;
set cohort_tp_tpt2;
dup=0;
if ikn=(removed for privacy) or ikn=(removed for privacy) then dup=1;
run;

data duplicates (where=(dup=1));
set duplicates;
run;

data duplicates (drop=iphis_accurateepisodedate iphis_disease
iphis_countryofbirth iphis_travelassociated iphis_traveldestination1);
set duplicates;
run;

data duplicates;
set duplicates;
length rec $20;
rec='rec2';
run;
```

```
data duplicates;
set duplicates;
length ikn2 $20;
ikn2=catx("_", of ikn rec);
run;

data duplicates (drop=ikn dup _a rec);
set duplicates;
run;

data duplicates;
rename ikn2=ikn iphis_accurateepisodedate_rec2=iphis_accurateepisodedate
iphis_disease_rec2=iphis_disease
iphis_countryofbirth_rec2=iphis_countryofbirth
iphis_travelassociated_rec2=iphis_travelassociated
iphis_traveldestination1_rec2=iphis_traveldestination1;
set duplicates;
run;

data duplicates;
set duplicates;
run;

/*now append to cohort of TP*/

data cohort_tp_tpt3 (drop=iphis_accurateepisodedate_rec2 iphis_disease_rec2
iphis_countryofbirth_rec2 iphis_travelassociated_rec2
iphis_traveldestination1_rec2);
set cohort_tp_tpt2;
run;

data cohort_tp_tpt3;
set cohort_tp_tpt3;
length ikn2 $20;
ikn2=ikn;
run;

data cohort_tp_tpt3 (drop=ikn);
set cohort_tp_tpt3;
run;

data cohort_tp_tpt3 (rename=ikn2=ikn);
set cohort_tp_tpt3;
run;

proc append base=cohort_tp_tpt3 data=duplicates;
run;

data cohort_tp_tpt_append;
set cohort_tp_tpt3;
run;


data cohort_tp_tpt_append;
retain ikn iphis iphis_accurateepisodedate iphis_disease iphis_countryofbirth
iphis_travelassociated iphis_traveldestination1
```

```
first_admin_code_date cohort_entry age_cohort_entry sex cic_immigrant
cic_fsrce   admin_code_in_lookback elig_at_iphis elig_1yr
elig_2yr elig_3yr source date ddate dxcode dx10code_1 dxtype_1 dxpref_1
      dx10code_2 dxtype_2     dxpref_2 dx10code_3     dxtype_3 dxpref_3
peel_region age   diseasegp tpt;
set cohort_tp_tpt3;
run;

data cohort_tp_tpt_append;
set cohort_tp_tpt_append;
run;


/*how many unique individuals?*/

proc sort data=cohort_tp_tpt_append;
by ikn first_admin_code_date;
run;

data uniqueikn_tpt;
set cohort_tp_tpt_append;
if first.ikn then count=0;
count + 1;
if last.ikn then output;
by ikn;
run;


proc freq data=cohort_tp_tpt_append;
tables dxcode dx10code:;
run;

/*Look at time between first and last date in individuals with >1 episode*/

data cohort_tp_tpt_append;
set cohort_tp_tpt_append;
datediffo=date-first_admin_code_date;
run;

proc sort data=cohort_tp_tpt_append;
by ikn first_admin_code_date date;
run;

data episodeperiod_tpt2;
set cohort_tp_tpt_append;
if first.ikn then count=0;
count + 1;
if last.ikn then output;
by ikn;
run;

proc sort data=episodeperiod_tpt2;
by datediffo;
run;

proc means mean min max median data=episodeperiod_tpt2;
var datediffo count;
```

```
run;


/*Look at time between iPHIS episode date (onset) and health encounter dates
in admin data*/

data cohort_tp_tpt2;
set cohort_tp_tpt_append;
datediffiphis=date - iphis_accurateepisodedate;
run;


proc freq data=cohort_tp_tpt2;
tables datediffiphis;
run;

proc univariate data=cohort_tp_tpt2;
var datediffiphis;
histogram;
run;

proc means mean min max median data=cohort_tp_tpt2;
var datediffiphis;
run;

data cohort_tp_tpt2;
set cohort_tp_tpt2;
if          datediffiphis < -7 then iphisdategp=1;
else if datediffiphis ge -7 and datediffiphis le 30 then iphisdategp=2;
else if datediffiphis > 30     then iphisdategp=3;
run;

proc freq data=cohort_tp_tpt2;
tables iphisdategp;
run;

proc sort data=cohort_tp_tpt2;
by iphisdategp;
run;

proc freq data=cohort_tp_tpt2;
by iphisdategp;
tables dxcode;
run;

proc freq data=cohort_tp_tpt2;
by iphisdategp;
tables dx10code_1;
run;


/*what is distribution like for most specific codes?*/

proc freq data=cohort_tp_tpt2;
where dxcode in: ('002');
tables datediffiphis;
```

```
run;

proc freq data=cohort_tp_tpt2;
where dxcode in: ('009');
tables datediffiphis;
run;

proc freq data=cohort_tp_tpt2;
where dx10code_1 in: ('A01');
tables datediffiphis;
run;

proc freq data=cohort_tp_tpt2;
where dxcode in: ('787');
tables datediffiphis;
run;

proc univariate data=cohort_tp_tpt2;
where dxcode in: ('002');
var datediffiphis;
histogram;
run;

proc univariate data=cohort_tp_tpt2;
where dxcode in: ('009');
var datediffiphis;
histogram;
run;

proc univariate data=cohort_tp_tpt2;
where dx10code_1 in: ('A01') or dx10code_2 in: ('A01') or dx10code_3 in:
('A01') ;
var datediffiphis;
histogram;
run;

proc univariate data=cohort_tp_tpt2;
where dxcode in: ('787');
var datediffiphis;
histogram;
run;


proc freq data=cohort_tp_tpt2;
tables datediffiphis*dxcode /nopercent nocol norow;
run;


data cohort_tp_tpt2;
set cohort_tp_tpt2;
if          datediffiphis < -30 then iphisdategp2=1;
else if datediffiphis ge -30 and datediffiphis le 120 then iphisdategp2=2;
else if datediffiphis > 120   then iphisdategp2=3;
run;

proc freq data=cohort_tp_tpt2;
tables iphisdategp2;
```

```
run;

proc sort data=cohort_tp_tpt2;
by iphisdategp2;
run;

proc freq data=cohort_tp_tpt2;
by iphisdategp2;
tables dxcode;
run;

proc freq data=cohort_tp_tpt2;
by iphisdategp2;
tables dx10code_1;
run;


/*look for False Negatives*/
data cohort_tp_tpt2_fn (keep=ikn iphis_accurateepisodedate source date ddate
dxcode dx10code_1 dx10code_2 dx10code_3 datediffiphis iphisdategp2);
set cohort_tp_tpt2;
run;

proc sort data=cohort_tp_tpt2_fn;
by ikn date;
run;

/*11 total*/

/*based on looking at cases, upper cutoff of 200 seems reasonable with codes
787, 009*/


data cohort_tp_tpt2;
set cohort_tp_tpt2;
if           datediffiphis < -30 then iphisdategp3=1;
else if datediffiphis ge -30 and datediffiphis le 200 then iphisdategp3=2;
else if datediffiphis > 200   then iphisdategp3=3;
run;


/*Apply rule:  IF codes 787 or 009 AND iphisdategp3 = 1 or 3, exclude*/

data cohort_tp_tpt3;
set cohort_tp_tpt2;
fn=0;
if    dxcode in: ('009' '787' '136') and (iphisdategp3 ne 2)      then fn=1;
else if dx10code_1 in: ('A099') and (iphisdategp3 ne 2)           then fn=1;
run;

proc freq data=cohort_tp_tpt3;
tables fn;
run;

proc sort data=cohort_tp_tpt3;
by fn ikn;
run;
```

```
data cohort_tp_tpt4 (where=(fn ne 1));
set cohort_tp_tpt3;
run;


/*how many unique individuals?*/

proc sort data=cohort_tp_tpt4;
by ikn first_admin_code_date;
run;

data uniqueikn_tpt;
set cohort_tp_tpt4;
if first.ikn then count=0;
count + 1;
if last.ikn then output;
by ikn;
run; /*121 unique IKNs*/


/*re-run descriptive excluding FN*/
proc freq data=cohort_tp_tpt4;
tables dxcode dx10code:;
run;

proc sort data=cohort_tp_tpt4;
by ikn first_admin_code_date date;
run;

data episodeperiod_tpt4;
set cohort_tp_tpt4;
if first.ikn then count=0;
count + 1;
if last.ikn then output;
by ikn;
run;

proc means mean median min max data=episodeperiod_tpt4;
var datediffo count;
run;


/*Now collapse to look at constellation of codes + VENN diagram (still need
to exclude some episodes but leave in for now)*/

/*need to flatten only one row per ikn/individual level*/


data cohort_tp_tpt4 (rename=(dx10code_1=dx10code1_ dx10code_2=dx10code2_
dx10code_3=dx10code3_));
set cohort_tp_tpt4;
run;

%MAKEWIDE(data          = cohort_tp_tpt4,
           out          = source_wide,
           id           = ikn,
```

```
            var          = source);

data source_wide;
set source_wide;
run;

%MAKEWIDE(data           = cohort_tp_tpt4,
            out          = date_wide,
            id           = ikn,
            var          = date);

data date_wide;
set date_wide;
run;

%MAKEWIDE(data           = cohort_tp_tpt4,
            out          = dxcode_wide,
            id           = ikn,
            var          = dxcode);

data dxcode_wide;
set dxcode_wide;
run;

%MAKEWIDE(data           = cohort_tp_tpt4,
            out          = dx10code1_wide,
            id           = ikn,
            var          = dx10code1_);

data dx10code1_wide;
set dx10code1_wide;
run;

%MAKEWIDE(data           = cohort_tp_tpt4,
            out          = dx10code2_wide,
            id           = ikn,
            var          = dx10code2_);

data dx10code2_wide;
set dx10code2_wide;
run;

%MAKEWIDE(data           = cohort_tp_tpt4,
            out          = dx10code3_wide,
            id           = ikn,
            var          = dx10code3_);
data dx10code3_wide;
set dx10code3_wide;
run;

/*now merge together*/
data tpt_wide2;
merge source_wide
date_wide
dxcode_wide
dx10code1_wide
dx10code2_wide
```

```
dx10code3_wide;
by ikn;
run;


/*Look at constellation*/

data t1;
set tpt_wide2;
array dxc[25] $ dxcode1-dxcode25;
array dx10c1[25] $dx10code1_1-dx10code1_25;
array dx10c2[25] $dx10code2_1-dx10code2_25;
array dx10c3[25] $dx10code3_1-dx10code3_25;
h_sp=0;
do i=1 to 25;
      if           dxc[i] in: ('002')      then h_sp=1;
      else if dx10c1[i] in: ('A01')        then h_sp=1;
      else if dx10c2[i] in: ('A01')        then h_sp=1;
      else if dx10c3[i] in: ('A01')        then h_sp=1;
      end;
      run;

proc freq data=t1;
tables h_sp;
run; /*86/97 meet this criteria - now 109/121*/


/*2 - moderate specificity (m_sp)*/

data t1;
set t1;
array dxc[25] $ dxcode1-dxcode25;
array dx10c1[25] $dx10code1_1-dx10code1_25;
array dx10c2[25] $dx10code2_1-dx10code2_25;
array dx10c3[25] $dx10code3_1-dx10code3_25;
m_sp=0;
do i=1 to 25;
      if           dxc[i] in:('009')       then m_sp=1;
      else if dx10c1[i] in: ('A029')       then m_sp=1;
      else if dx10c2[i] in: ('A029')       then m_sp=1;
      else if dx10c3[i] in: ('A029')       then m_sp=1;
      end;
      run;

proc freq data=t1;
tables m_sp;
run; /*37 meet this criteria*/


/*3 - low specificity (l_sp)*/

data t1;
set t1;
array dxc[25] $ dxcode1-dxcode25;
array dx10c1[25] $dx10code1_1-dx10code1_25;
array dx10c2[25] $dx10code2_1-dx10code2_25;
array dx10c3[25] $dx10code3_1-dx10code3_25;
```

```
l_sp=0;
do i=1 to 25;
      if          dxc[i] in: ('003' '005' '136' '784' '787') then l_sp=1;
      else if dx10c1[i] in: ('A021' 'A049' 'A059' 'A09' 'A499') then l_sp=1;
      else if dx10c2[i] in: ('A021' 'A049' 'A059' 'A09' 'A499') then l_sp=1;
      else if dx10c3[i] in: ('A021' 'A049' 'A059' 'A09' 'A499') then l_sp=1;
      end;
      run;

proc freq data=t1;
tables l_sp;
run;


/*h or m*/

data t1;
set t1;
hm_sp=0;
if h_sp=1 or m_sp=1 then hm_sp=1;
run;

/*h or m or l*/

data t1;
set t1;
hml_sp=0;
if h_sp=1 or m_sp=1 or l_sp=1 then hml_sp=1;
run;

/*only high*/

data t1;
set t1;
onlyh_sp=0;
if h_sp=1 and m_sp=0 and l_sp=0 then onlyh_sp=1;
run;

/*only low*/

data t1;
set t1;
onlyl_sp=0;
if h_sp=0 and m_sp=0 and l_sp=1 then onlyl_sp=1;
run;

/*all three*/

data t1;
set t1;
all_sp=0;
if h_sp=1 and m_sp=1 and l_sp=1 then all_sp=1;
run;

proc freq data=t1;
tables hm_sp hml_sp onlyh_sp onlyl_sp all_sp;
run;
```

```
/*H and M, no L*/

data t1;
set t1;
ab_sp=0;
if h_sp=1 and m_sp=1 and l_sp=0 then ab_sp=1;
run;

/*H and L, no M*/

data t1;
set t1;
ac_sp=0;
if h_sp=1 and m_sp=0 and l_sp=1 then ac_sp=1;
run;

/*M and L, no H*/

data t1;
set t1;
bc_sp=0;
if h_sp=0 and m_sp=1 and l_sp=1 then bc_sp=1;
run;

proc freq data=t1;
tables ab_sp ac_sp bc_sp;
run;

/*only M*/

data t1;
set t1;
b_sp=0;
if h_sp=0 and m_sp=1 and l_sp=0 then b_sp=1;
run;

proc freq data=t1;
tables b_sp;
run;


/*look at specific codes and create VENN diagram*/
data t1;
set t1;
array dxc[25] $ dxcode1-dxcode25;
array dx10c1[25] $dx10code1_1-dx10code1_25;
array dx10c2[25] $dx10code2_1-dx10code2_25;
array dx10c3[25] $dx10code3_1-dx10code3_25;
codeA01=0;
do i=1 to 25;
      if dx10c1[i]            in: ('A01') then codeA01=1;
      else if dx10c2[i]       in: ('A01') then codeA01=1;
      else if dx10c3[i]       in: ('A01') then codeA01=1;
      end;
      run;
```

```
data t1;
set t1;
array dxc[25] $ dxcode1-dxcode25;
array dx10c1[25] $dx10code1_1-dx10code1_25;
array dx10c2[25] $dx10code2_1-dx10code2_25;
array dx10c3[25] $dx10code3_1-dx10code3_25;
code002=0;
do i=1 to 25;
      if    dxc[i]             in: ('002') then code002=1;
      end;
      run;

data t1;
set t1;
array dxc[25] $ dxcode1-dxcode25;
array dx10c1[25] $dx10code1_1-dx10code1_25;
array dx10c2[25] $dx10code2_1-dx10code2_25;
array dx10c3[25] $dx10code3_1-dx10code3_25;
code787=0;
do i=1 to 25;
      if    dxc[i]             in: ('787') then code787=1;
      end;
      run;

proc freq data=t1;
tables codeA01 code002 code787;
run;

data t1;
set t1;
a=0;
if codeA01=1 and code002=0 and code787=0 then a=1;
run;

data t1;
set t1;
b=0;
if codeA01=0 and code002=1 and code787=0 then b=1;
run;

data t1;
set t1;
c=0;
if codeA01=0 and code002=0 and code787=1 then c=1;
run;

data t1;
set t1;
ab=0;
if codeA01=1 and code002=1 and code787=0  then ab=1;
run;

data t1;
set t1;
ac=0;
if codeA01=1 and code002=0 and code787=1  then ac=1;
```

```
run;

data t1;
set t1;
bc=0;
if codeA01=0 and code002=1 and code787=1 then bc=1;
run;

data t1;
set t1;
abc=0;
if codeA01=1 and code002=1 and code787=1 then abc=1;
run;

proc freq data=t1;
tables a b c ab ac bc abc;
run;

data t1;
set t1;
other=0;
if a=0 and b=0 and c=0 and ab=0 and ac=0 and bc=0 and abc=0 then other=1;
run;

proc freq data=t1;
tables other;
run; /*2*/


/*describe prevalence of other codes*/
data t1;
set t1;
array dxc[25] $ dxcode1-dxcode25;
array dx10c1[25] $dx10code1_1-dx10code1_25;
array dx10c2[25] $dx10code2_1-dx10code2_25;
array dx10c3[25] $dx10code3_1-dx10code3_25;
codeA02=0;
do i=1 to 25;
      if dx10c1[i]            in: ('A021' 'A029')     then codeA02=1;
      else if dx10c2[i]       in: ('A021' 'A029')     then codeA02=1;
      else if dx10c3[i]       in: ('A021' 'A029')     then codeA02=1;
      end;
      run;

data t1;
set t1;
array dxc[25] $ dxcode1-dxcode25;
array dx10c1[25] $dx10code1_1-dx10code1_25;
array dx10c2[25] $dx10code2_1-dx10code2_25;
array dx10c3[25] $dx10code3_1-dx10code3_25;
code009=0;
do i=1 to 25;
      if    dxc[i]            in: ('009') then code009=1;
      end;
      run;

data t1;
```

```
set t1;
array dxc[25] $ dxcode1-dxcode25;
array dx10c1[25] $dx10code1_1-dx10code1_25;
array dx10c2[25] $dx10code2_1-dx10code2_25;
array dx10c3[25] $dx10code3_1-dx10code3_25;
code003=0;
do i=1 to 25;
      if    dxc[i] in: ('003')      then code003=1;
      end;
      run;

data t1;
set t1;
array dxc[25] $ dxcode1-dxcode25;
array dx10c1[25] $dx10code1_1-dx10code1_25;
array dx10c2[25] $dx10code2_1-dx10code2_25;
array dx10c3[25] $dx10code3_1-dx10code3_25;
code136=0;
do i=1 to 25;
      if    dxc[i] in: ('136')      then code136=1;
      end;
      run;

proc freq data=t1;
tables codeA02 code009 code003 code136;
run;


data t1;
set t1;
array s[25] $ source1-source25;
hosp=0;
do i=1 to 25;
      if    s[i] in: ('DAD') then hosp=1;
      end;
      run;

data t1;
set t1;
array s[25] $ source1-source25;
ed=0;
do i=1 to 25;
      if    s[i] in: ('NACRS') then ed=1;
      end;
      run;

data t1;
set t1;
array s[25] $ source1-source25;
pc=0;
do i=1 to 25;
      if    s[i] in: ('OHIP') then pc=1;
      end;
      run;

data t1;
set t1;
```

```
array s[25] $ source1-source25;
sds=0;
do i=1 to 25;
      if    s[i] in: ('SDS') then sds=1;
      end;
      run;

proc freq data=t1;
tables hosp ed pc sds;
run;


/*now merge with episodeperiod_mal to get datediffo and count summaries*/

data epi_tpt (keep=ikn datediffo count);
set episodeperiod_tpt4;
run;

proc sort data=epi_tpt;
by ikn;
run;

data t2_summary;
merge epi_tpt (in=period) t1(in=typ);
by ikn;
if period and typ;
run;

proc means mean median min max data=t2_summary;
var datediffo count;
run;


/*now merge back in with original cohort to describe characteristics of TPs*/

/*create data set from original cohort with 1 obs per patient based on first
health encounter*/
proc sort data=cohort;
by ikn first_admin_code_date;
run;

data uniqueikn;
set cohort;
if first.ikn then count=0;
count + 1;
if last.ikn then output;
by ikn;
run;

data t3 (keep=ikn cohort_entry age_cohort_entry sex cic_immigrant cic_fsrce
admin_code_in_lookback);
set uniqueikn;
run;

data t4;
merge t3 (in=cohort) t2_summary(in=tpt);
by ikn;
```

```
if cohort and tpt;
run;


data t4;
set t4;
agegp=.;
if    age_cohort_entry < 18                                    then agegp=1;
else if age_cohort_entry ge 18 and age_cohort_entry le 64   then agegp=2;
else if age_cohort_entry ge 65                                 then agegp=3;
run;

proc univariate data=t4;
var age_cohort_entry;
run;


data t4;
set t4;
length region $20;
if          cic_fsrce in ('Asia and Pacific')then region='Asia and Pacific';
else if cic_fsrce ne (' ')                          then region='Other';
run;

proc freq data=t4;
tables region*cic_fsrce;
run;


proc freq data=t4;
tables agegp sex cic_immigrant region admin_code_in_lookback;
run;


data t4;
set t4;
array dx10c1[25] $dx10code1_1-dx10code1_25;
array dx10c2[25] $dx10code2_1-dx10code2_25;
array dx10c3[25] $dx10code3_1-dx10code3_25;
c_A021=0;
do i=1 to 25;
      if dx10c1[i] in: ('A021')           then c_A021=1;
      else if dx10c2[i] in: ('A021')      then c_A021=1;
      else if dx10c3[i] in: ('A021')      then c_A021=1;
      end;
      run;

data t4;
set t4;
array dx10c1[25] $dx10code1_1-dx10code1_25;
array dx10c2[25] $dx10code2_1-dx10code2_25;
array dx10c3[25] $dx10code3_1-dx10code3_25;
c_A029=0;
do i=1 to 25;
      if dx10c1[i] in: ('A029')           then c_A029=1;
      else if dx10c2[i] in: ('A029')      then c_A029=1;
      else if dx10c3[i] in: ('A029')      then c_A029=1;
```

```
      end;
      run;

data t4;
set t4;
array dx10c1[25] $dx10code1_1-dx10code1_25;
array dx10c2[25] $dx10code2_1-dx10code2_25;
array dx10c3[25] $dx10code3_1-dx10code3_25;
c_A049=0;
do i=1 to 25;
      if dx10c1[i] in: ('A049')           then c_A049=1;
      else if dx10c2[i] in: ('A049')      then c_A049=1;
      else if dx10c3[i] in: ('A049')      then c_A049=1;
      end;
      run;

data t4;
set t4;
array dx10c1[25] $dx10code1_1-dx10code1_25;
array dx10c2[25] $dx10code2_1-dx10code2_25;
array dx10c3[25] $dx10code3_1-dx10code3_25;
c_A059=0;
do i=1 to 25;
      if dx10c1[i] in: ('A059')           then c_A059=1;
      else if dx10c2[i] in: ('A059')      then c_A059=1;
      else if dx10c3[i] in: ('A059')      then c_A059=1;
      end;
      run;

data t4;
set t4;
array dx10c1[25] $dx10code1_1-dx10code1_25;
array dx10c2[25] $dx10code2_1-dx10code2_25;
array dx10c3[25] $dx10code3_1-dx10code3_25;
c_A09=0;
do i=1 to 25;
      if dx10c1[i] in: ('A09')            then c_A09=1;
      else if dx10c2[i] in: ('A09')       then c_A09=1;
      else if dx10c3[i] in: ('A09')       then c_A09=1;
      end;
      run;

data t4;
set t4;
array dx10c1[25] $dx10code1_1-dx10code1_25;
array dx10c2[25] $dx10code2_1-dx10code2_25;
array dx10c3[25] $dx10code3_1-dx10code3_25;
c_A499=0;
do i=1 to 25;
      if dx10c1[i] in: ('A499')           then c_A499=1;
      else if dx10c2[i] in: ('A499')      then c_A499=1;
      else if dx10c3[i] in: ('A499')      then c_A499=1;
      end;
      run;

proc freq data=t4;
tables c_A021 c_A029 c_A049 c_A059 c_A09 c_A499;
```

```
run;

data t4;
set t4;
if c_A049=1 or c_A059=1 then c_A04959=1;
run;

proc freq data=t4;
tables c_A04959;
run;


/*save*/
data DATASETS.final_tpt_tp_02may2017;
set t4;
run; /*final T/PT TP dataset - can restrict to h_sp=1 to look at only high
sp*/


data t4;
set t4;
run;



/******HIGH SPECIFICITY TP ANALYSIS*******/

/*Merge high spec back with cohort to describe TP*/

data t5 (where=(h_sp=1));
set t4;
run;


proc univariate data=t5;
var age_cohort_entry;
histogram;
run;


data t5;
set t5;
agegp=.;
if     age_cohort_entry < 18   then agegp=1;
else if age_cohort_entry ge 18 and age_cohort_entry le 64   then agegp=2;
else if age_cohort_entry ge 65 then agegp=3;
run;

data t5;
set t5;
length region $20;
if     cic_fsrce in ('Asia and Pacific')   then region='Asia and Pacific';
else if cic_fsrce ne (' ')                 then region='Other';
run;


proc freq data=t5;
```

```
tables agegp sex cic_immigrant region admin_code_in_lookback;
run;

proc freq data=t5;
tables codeA01 codeA02 code002 code009 code003 code136 code787;
run;

proc freq data=t5;
tables c_A021 c_A029 c_A049 c_A059 c_A09 c_A499 c_A049*c_A059;
run;

proc freq data=t5;
tables hosp ed pc sds;
run;


/*Go back to describe high spec TPs at the observation level, not patient
level*/

data t6 (keep=ikn h_sp);
set t5;
run;

/*now need to indicate duplicate ikns so counted twice*/

data t6;
set t6;
if first.ikn then id_count=1;
by ikn;
run;

data tduplicates;
set t6;
length rec $20;
if id_count=. then rec='rec2';
run;

data tduplicates;
set tduplicates;
length ikn2 $20;
ikn2=catx("_", of ikn rec);
run;

data tduplicates (drop=ikn id_count rec);
set tduplicates;
run;

data tduplicates (rename=(ikn2=ikn));
set tduplicates;
run;

/*now can merge with original cohort and both records should stay*/

data t7;
merge tduplicates (in=tpthsp) cohort_tp_tpt4 (in=cohort);
by ikn;
if tpthsp and cohort;
```

```
run;

data t7;
set t7;
run;


/*how many unique individuals?*/

proc sort data=t7;
by ikn first_admin_code_date;
run;

data uniqueikn_tpt_t7;
set t7;
if first.ikn then count=0;
count + 1;
if last.ikn then output;
by ikn;
run; /*109 unique IKNs*/


/*describe code distribution*/
proc freq data=t7;
tables dxcode dx10code:;
run;



/*Look at time between first and last date in individuals with >1 episode*/

proc sort data=t7;
by ikn first_admin_code_date date;
run;

data episodeperiod_tpt_t7;
set t7;
if first.ikn then count=0;
count + 1;
if last.ikn then output;
by ikn;
run;

proc means mean median min max data=episodeperiod_tpt_t7;
var count datediffo;
run;


/*Look at time between iPHIS episode date (onset) and health encounter dates
in admin data*/

proc freq data=t7;
tables datediffiphis;
run;

proc means mean min max median data=t7;
var datediffiphis;
```

```
run;

proc freq data=t7;
tables iphisdategp2;
run;


/*describe codes*/

proc freq data=t5;
tables h_sp onlyh_sp l_sp all_sp;
run;

proc freq data=t5;
tables a b c ab ac bc abc other;
run;

proc freq data=t5;
tables codeA01*code002;
run;



/*Bivariate macro to compare TP and FP (Table 3)*/

/*1. start by combining for hep A*/

data hep_tp;
set DATASETS.final_hepa_tp_02may2017;
run;

data hep_tp (keep=ikn sex age_cohort_entry cic_immigrant
admin_code_in_lookback hosp ed pc sds code070 codeb15 datediffo count h_sp);
set hep_tp;
run;

data hep_tp (where=(h_sp=1));
set hep_tp;
run;

data hep_tp;
set hep_tp;
outcome=1;
run;

data hep_fp;
set DATASETS.final_hepa_fp_02may2017;
run;

data hep_fp (keep=ikn sex age_cohort_entry cic_immigrant
admin_code_in_lookback hosp ed pc sds c_070 c_B15 datediffo count h_sp);
set hep_fp;
run;

data hep_fp (where=(h_sp=1));
set hep_fp;
run;
```

```
data hep_fp (rename=(c_070=code070 c_B15=codeb15));
set hep_fp;
run;

data hep_fp;
set hep_fp;
outcome=0;
run;

proc append base=hep_tp data=hep_fp;
run;

data allhep;
set hep_tp;
run;

data allhep;
set allhep;
run;

data allhep;
set allhep;
if hosp=1 or ed=1 then hosped=1;
else if hosped=. then hosped=0;
run;

data allhep;
set allhep;
if sex='F' then sexm=1;
else if sex='M' then sexm=0;
run;


/*bivariate comparisons*/
%bivariate (
data              = allhep,
byvar             = outcome,
median            = age_cohort_entry datediffo count,
categorical       = sexm cic_immigrant admin_code_in_lookback hosped hosp ed
pc,
title             = "Comparing TP and FP, Hepatitis A",
file              = tabfile/hep.txt,
print             = F,
pvalue            = T,
label             = T
           );



/*2. Malaria*/

data mal_tp;
set DATASETS.final_mal_tp_02may2017;
run;
```

```
data mal_tp (keep=ikn sex age_cohort_entry cic_immigrant
admin_code_in_lookback hosp ed pc sds codeb5 datediffo count h_sp);
set mal_tp;
run;

data mal_tp (where=(h_sp=1));
set mal_tp;
run;

data mal_tp;
set mal_tp;
outcome=1;
run;

data mal_fp;
set DATASETS.final_mal_fp_02may2017;
run;

data mal_fp (keep=ikn sex age_cohort_entry cic_immigrant
admin_code_in_lookback hosp ed pc sds c_B5 datediffo count h_sp);
set mal_fp;
run;

data mal_fp (where=(h_sp=1));
set mal_fp;
run;

data mal_fp (rename=(c_B5=codeB5));
set mal_fp;
run;

data mal_fp;
set mal_fp;
outcome=0;
run;

proc append base=mal_tp data=mal_fp;
run;

data allmal;
set mal_tp;
run;

data allmal;
set allmal;
if hosp=1 or ed=1 then hosped=1;
else if hosped=. then hosped=0;
run;

data allmal;
set allmal;
if sex='F' then sexm=1;
else if sex='M' then sexm=0;
run;


/*bivariate comparisons*/
```

```
%bivariate (
data              = allmal,
byvar             = outcome,
median            = age_cohort_entry datediffo count,
categorical       = sexm cic_immigrant admin_code_in_lookback hosp ed pc
hosped,
title             = "Comparing TP and FP, Malaria",
file              = tabfile/mal.txt,
print             = F,
pvalue            = T,
label             = T
           );  /*sex, and immigrant status not sign at P<0.2 level*/



/*3. T/PT*/

data tpt_tp;
set DATASETS.final_tpt_tp_02may2017;
run;

data tpt_tp (keep=ikn sex age_cohort_entry cic_immigrant
admin_code_in_lookback hosp ed pc sds datediffo count h_sp);
set tpt_tp;
run;

data tpt_tp (where=(h_sp=1));
set tpt_tp;
run;

data tpt_tp;
set tpt_tp;
outcome=1;
run;

data tpt_fp;
set DATASETS.final_tpt_fp_02may2017;
run;

data tpt_fp (keep=ikn sex age_cohort_entry cic_immigrant
admin_code_in_lookback hosp ed pc sds datediffo count h_sp);
set tpt_fp;
run;

data tpt_fp (where=(h_sp=1));
set tpt_fp;
run;

data tpt_fp;
set tpt_fp;
outcome=0;
run;

proc append base=tpt_tp data=tpt_fp;
run;

data alltpt;
```

```
set tpt_tp;
run;

data alltpt;
set alltpt;
if hosp=1 or ed=1 then hosped=1;
else if hosped=. then hosped=0;
run;

data alltpt;
set alltpt;
if sex='F' then sexm=1;
else if sex='M' then sexm=0;
run;


/*bivariate comparisons*/
%bivariate (
data            = alltpt,
byvar           = outcome,
median          = age_cohort_entry datediffo count,
categorical     = sexm cic_immigrant admin_code_in_lookback hosp ed pc
hosped,
title           = "Comparing TP and FP, TPT",
file            = tabfile/tpt.txt,
print           = F,
pvalue          = T,
label           = T
           );  /*sex, and immigrant status not sign at P<0.2 level*/



/*** FALSE NEGATIVES FOR HIGH SPEC ANALYSIS ***/


data cohort_tp (where=(iphis=1 and first_admin_code_date ne .));
set cohort;
run; /*1601 observations*/

data cohort_tp;
set cohort_tp;
if    iphis_disease in ("HEPATITIS A")    then diseasegp=1;
else if iphis_disease in ("MALARIA")      then diseasegp=2;
else if iphis_disease in ("PARATYPHOID FEVER" "TYPHOID FEVER") then
diseasegp=3;
run;


/**********HEPA**********/

data cohort_tp_hep (where=(diseasegp=1));
set cohort_tp;
run;

data episodecount;
set cohort_tp_hep;
```

```
if first.ikn then count=0;
count + 1;
if last.ikn then output;
by ikn;
run;

proc means min max data=episodecount;
var count;
run; /*18*/


/*flatten*/
data hep (rename=(dx10code_1=dx10code1_ dx10code_2=dx10code2_
dx10code_3=dx10code3_));
set cohort_tp_hep;
run;

%MAKEWIDE(data          = hep,
           out          = source_wide,
           id           = ikn,
           var          = source);

data source_wide;
set source_wide;
run;

%MAKEWIDE(data          = hep,
           out          = date_wide,
           id           = ikn,
           var          = date);

data date_wide;
set date_wide;
run;

%MAKEWIDE(data          = hep,
           out          = dxcode_wide,
           id           = ikn,
           var          = dxcode);

data dxcode_wide;
set dxcode_wide;
run;

%MAKEWIDE(data          = hep,
           out          = dx10code1_wide,
           id           = ikn,
           var          = dx10code1_);

data dx10code1_wide;
set dx10code1_wide;
run;

%MAKEWIDE(data          = hep,
           out          = dx10code2_wide,
           id           = ikn,
           var          = dx10code2_);
```

```
data dx10code2_wide;
set dx10code2_wide;
run;

%MAKEWIDE(data          = hep,
          out           = dx10code3_wide,
          id            = ikn,
          var           = dx10code3_);

data dx10code3_wide;
set dx10code3_wide;
run;


/*now merge together*/
data hep_wide;
merge source_wide
date_wide
dxcode_wide
dx10code1_wide
dx10code2_wide
dx10code3_wide;
by ikn;
run;

data hep_wide;
set hep_wide;
run;

data hep_wide;
set hep_wide;
array dxc[18] $ dxcode1-dxcode18;
array dx10c1[18] $dx10code1_1-dx10code1_18;
array dx10c2[18] $dx10code2_1-dx10code2_18;
array dx10c3[18] $dx10code3_1-dx10code3_18;
h_sp=0;
do i=1 to 18;
      if           dxc[i] in:('070')       then h_sp=1;
      else if dx10c1[i] in: ('B15')        then h_sp=1;
      else if dx10c2[i] in: ('B15')        then h_sp=1;
      else if dx10c3[i] in: ('B15')        then h_sp=1;
      end;
      run;

proc freq data=hep_wide;
tables h_sp;
run;

data hep_fn (where=(h_sp ne 1));
set hep_wide;
run;

data hep_fn;
set hep_fn;
hep_fn=1;
run;
```

```
data hep_fn (keep=ikn hep_fn);
set hep_fn;
run;


/***MALARIA***/

data cohort_tp_mal (where=(diseasegp=2));
set cohort_tp;
run;

data episodecount;
set cohort_tp_mal;
if first.ikn then count=0;
count + 1;
if last.ikn then output;
by ikn;
run;

proc means min max data=episodecount;
var count;
run; /*37*/


/*flatten*/
data mal (rename=(dx10code_1=dx10code1_ dx10code_2=dx10code2_
dx10code_3=dx10code3_));
set cohort_tp_mal;
run;

%MAKEWIDE(data          = mal,
          out           = source_wide,
          id            = ikn,
          var           = source);

data source_wide;
set source_wide;
run;

%MAKEWIDE(data          = mal,
          out           = date_wide,
          id            = ikn,
          var           = date);

data date_wide;
set date_wide;
run;

%MAKEWIDE(data          = mal,
          out           = dxcode_wide,
          id            = ikn,
          var           = dxcode);

data dxcode_wide;
set dxcode_wide;
run;
```

```
%MAKEWIDE(data          = mal,
          out           = dx10code1_wide,
          id            = ikn,
          var           = dx10code1_);

data dx10code1_wide;
set dx10code1_wide;
run;

%MAKEWIDE(data          = mal,
          out           = dx10code2_wide,
          id            = ikn,
          var           = dx10code2_);

data dx10code2_wide;
set dx10code2_wide;
run;

%MAKEWIDE(data          = mal,
          out           = dx10code3_wide,
          id            = ikn,
          var           = dx10code3_);

data dx10code3_wide;
set dx10code3_wide;
run;


/*now merge together*/
data mal_wide;
merge source_wide
date_wide
dxcode_wide
dx10code1_wide
dx10code2_wide
dx10code3_wide;
by ikn;
run;

data mal_wide;
set mal_wide;
run;


data mal_wide;
set mal_wide;
array dxc[37] $ dxcode1-dxcode37;
array dx10c1[37] $dx10code1_1-dx10code1_37;
array dx10c2[37] $dx10code2_1-dx10code2_37;
array dx10c3[37] $dx10code3_1-dx10code3_37;
h_sp=0;
do i=1 to 37;
      if           dxc[i] in:('062')             then h_sp=1;
      else if dx10c1[i] in: ('B5')  then h_sp=1;
      else if dx10c2[i] in: ('B5')  then h_sp=1;
      else if dx10c3[i] in: ('B5')  then h_sp=1;
```

```
      end;
      run;

proc freq data=mal_wide;
tables h_sp;
run;


data mal_fn (where=(h_sp ne 1));
set mal_wide;
run;

data mal_fn;
set mal_fn;
mal_fn=1;
run;

data mal_fn (keep=ikn mal_fn);
set mal_fn;
run;


/***TYPHOID AND PARATYPHOID***/

data cohort_tp_tpt (where=(diseasegp=3));
set cohort_tp;
run;

data episodecount;
set cohort_tp_tpt;
if first.ikn then count=0;
count + 1;
if last.ikn then output;
by ikn;
run;

proc means min max data=episodecount;
var count;
run; /*27*/


/*flatten*/
data tpt (rename=(dx10code_1=dx10code1_ dx10code_2=dx10code2_
dx10code_3=dx10code3_));
set cohort_tp_tpt;
run;

%MAKEWIDE(data          = tpt,
           out          = source_wide,
           id           = ikn,
           var          = source);

data source_wide;
set source_wide;
run;

%MAKEWIDE(data          = tpt,
```

```
           out          = date_wide,
           id           = ikn,
           var          = date);

data date_wide;
set date_wide;
run;

%MAKEWIDE(data          = tpt,
           out          = dxcode_wide,
           id           = ikn,
           var          = dxcode);

data dxcode_wide;
set dxcode_wide;
run;

%MAKEWIDE(data          = tpt,
           out          = dx10code1_wide,
           id           = ikn,
           var          = dx10code1_);

data dx10code1_wide;
set dx10code1_wide;
run;

%MAKEWIDE(data          = tpt,
           out          = dx10code2_wide,
           id           = ikn,
           var          = dx10code2_);

data dx10code2_wide;
set dx10code2_wide;
run;
%MAKEWIDE(data          = tpt,
           out          = dx10code3_wide,
           id           = ikn,
           var          = dx10code3_);

data dx10code3_wide;
set dx10code3_wide;
run;


/*now merge together*/
data tpt_wide;
merge source_wide
date_wide
dxcode_wide
dx10code1_wide
dx10code2_wide
dx10code3_wide;
by ikn;
run;

data tpt_wide;
set tpt_wide;
```

```
run;


data tpt_wide;
set tpt_wide;
array dxc[27] $ dxcode1-dxcode27;
array dx10c1[27] $dx10code1_1-dx10code1_27;
array dx10c2[27] $dx10code2_1-dx10code2_27;
array dx10c3[27] $dx10code3_1-dx10code3_27;
h_sp=0;
do i=1 to 27;
      if          dxc[i] in: ('002')      then h_sp=1;
      else if dx10c1[i] in: ('A01')       then h_sp=1;
      else if dx10c2[i] in: ('A01')       then h_sp=1;
      else if dx10c3[i] in: ('A01')       then h_sp=1;
      end;
      run;

proc freq data=tpt_wide;
tables h_sp;
run;


data tpt_fn (where=(h_sp ne 1));
set tpt_wide;
run;

data tpt_fn;
set tpt_fn;
tpt_fn=1;
run;

data tpt_fn (keep=ikn tpt_fn);
set tpt_fn;
run;

data tpt_wide;
set tpt_wide;
run;


/*3 FN datasets:  hep_fn, mal_fn and tpt_fn*
/*merge in with refstd to describe cases*/

data cohort_fn (where=(iphis=1 and first_admin_code_date = .));
set cohort;
run;

data cohort_fn;
set cohort_fn;
notinadmin=1;
run;

data cohort_fn (keep=ikn notinadmin);
set cohort_fn;
run;
```

```
data cohort_fn_all;
merge cohort_fn
hep_fn
mal_fn
tpt_fn;
by ikn;
run;


data cohort_fn_final;
merge cohort_fn_all(in=cohort) refstd (in=ref);
by ikn;
if cohort and ref; /*so that only keeps data for those in cohort*/
run;

proc freq data=cohort_fn_final;
tables notinadmin;
run; /*n=18 are ONLY in iPHIS*/

data cohort_fn_final;
set cohort_fn_final;
run;

proc freq data=cohort_fn_final;
tables disease;
run;

/*add back admin vars for all by merging with original cohort (first
record)*/

data cohort_fn_final (rename=sex=sexiphis);
set cohort_fn_final;
run;

proc sort data=cohort;
by ikn first_admin_code_date;
run;

data uniqueikn;
set cohort;
if first.ikn then count=0;
count + 1;
if last.ikn then output;
by ikn;
run;

data cohort2 (keep=ikn iphis sex age_cohort_entry cic_immigrant cic_fsrce
admin_code_in_lookback elig_at_iphis elig_1yr elig_2yr elig_3yr peel_region);
set uniqueikn;
run;

data cohort_fn_final2;
merge cohort2 (in=cohort2) cohort_fn_final(in=cohort);
by ikn;
if cohort2 and cohort;
run;
```

```
data cohort_fn_final2;
set cohort_fn_final2;
agegp=.;
if age_cohort_entry < 18 then agegp=1;
else if age_cohort_entry ge 18 and age_cohort_entry le 64 then agegp=2;
else if age_cohort_entry > 64 then agegp=3;
run;

proc univariate data=cohort_fn_final2;
var age_cohort_entry;
histogram;
run;

data cohort_fn_final2;
set cohort_fn_final2;
run;

proc freq data=cohort_fn_final2;
tables city sex agegp disease countryofbirth travelassociated;
run;

proc freq data=cohort_fn_final2;
tables interventiontype interventionlocation;
run;

proc freq data=cohort_fn_final2;
tables cic_immigrant cic_fsrce elig_at_iphis elig_1yr elig_2yr elig_3yr
peel_region admin_code_in_lookback;
run;

proc freq data=cohort_fn_final2;
tables peel_region*notinadmin;
run;

proc freq data=cohort_fn_final2;
tables sexiphis*sex;
run;

/*are the hosp for those with health encounters?*/
proc freq data=cohort_fn_final2;
tables interventiontype*notinadmin;
run;

data cohort_fn_final2;
set cohort_fn_final2;
if notinadmin=1 then na=1;
else if notinadmin=. then na=0;
run;

proc freq data=cohort_fn_final2;
where interventiontype='HOSPITALIZATION';
tables na;
run;


proc freq data=cohort_fn_final2;
tables elig_at_iphis*na;
```

```
run;


/*create perm dataset*/
data DATASETS.final_hsp_fn_02may2017;
set cohort_fn_final2;
run;


data fn;
set DATASETS.final_hsp_fn_02may2017;
run;

proc univariate data=fn;
var age_cohort_entry;
run;

*/

data cohort_fn_final2;
set DATASETS.final_hsp_fn_02may2017;
run;


/*what diagnostic codes did the FN who were in admin data have?*/

data fn_codes (where=(notinadmin ne 1));
set cohort_fn_final2;
run;

data fn_codes (keep=ikn notinadmin interventiontype hep_fn mal_fn tpt_fn);
set fn_codes;
run;

data fn_codes;
set fn_codes;
run;

data fn_codesa;
merge fn_codes (in=fn) cohort(in=cohort);
by ikn;
if fn and cohort;
run;

data uniqueikn;
set fn_codesa;
if last.ikn then output;
by ikn;
run;

proc freq data=fn_codesa;
tables dxcode dx10code_1 dx10code_2 dx10code_3;
run;

/*now make wide to describe at person level*/

data fn_codesb (rename=(dx10code_1=dx10code1_));
```

```
set fn_codesa;
run;

%MAKEWIDE(data          = fn_codesb,
          out           = dxcode_wide,
          id            = ikn,
          var           = dxcode);

data dxcode_wide;
set dxcode_wide;
run;

%MAKEWIDE(data          = fn_codesb,
          out           = dx10code1_wide,
          id            = ikn,
          var           = dx10code1_);

data dx10code1_wide;
set dx10code1_wide;
run;

%MAKEWIDE(data          = fn_codesb,
          out           = source_wide,
          id            = ikn,
          var           = source);

data source_wide;
set source_wide;
run;

data fn_codesc;
merge fn_codes
dxcode_wide
dx10code1_wide
source_wide;
by ikn;
run;

data fn_codesc;
set fn_codesc;
array dxc[20] $ dxcode1-dxcode20;
c009=0;
do i=1 to 20;
      if          dxc[i] in: ('009')            then c009=1;
      end;
      run;

data fn_codesc;
set fn_codesc;
array dxc[20] $ dxcode1-dxcode20;
c003=0;
do i=1 to 20;
      if          dxc[i] in: ('003')            then c003=1;
      end;
      run;

data fn_codesc;
```

```
set fn_codesc;
array dxc[20] $ dxcode1-dxcode20;
c070=0;
do i=1 to 20;
      if          dxc[i] in: ('070')             then c070=1;
      end;
      run;

data fn_codesc;
set fn_codesc;
array dxc[20] $ dxcode1-dxcode20;
c079=0;
do i=1 to 20;
      if          dxc[i] in: ('079')             then c079=1;
      end;
      run;

data fn_codesc;
set fn_codesc;
array dxc[20] $ dxcode1-dxcode20;
c136=0;
do i=1 to 20;
      if          dxc[i] in: ('136')             then c136=1;
      end;
      run;

data fn_codesc;
set fn_codesc;
array dxc[20] $ dxcode1-dxcode20;
c781=0;
do i=1 to 20;
      if          dxc[i] in: ('781')             then c781=1;
      end;
      run;

data fn_codesc;
set fn_codesc;
array dxc[20] $ dxcode1-dxcode20;
c787=0;
do i=1 to 20;
      if          dxc[i] in: ('787')             then c787=1;
      end;
      run;



proc freq data=fn_codesc;
tables c009 c003 c070 c079 c136 c781 c787;
run;


data fn_codesc;
set fn_codesc;
array dxc[20] $ dxcode1-dxcode20;
array dx10c1[20] $dx10code1_1-dx10code1_20;
array dx10c2[20] $dx10code2_1-dx10code2_20;
array dx10c3[20] $dx10code3_1-dx10code3_20;
```

```
other=0;
do i=1 to 20;
      if          dxc[i] in: ('002' '003' '070')      then other=1;
      else if dx10c1[i] in: ('A021' 'A09' 'A499')     then other=1;
      else if dx10c2[i] in: ('A021' 'A09' 'A499')     then other=1;
      else if dx10c3[i] in: ('A021' 'A09' 'A499')     then other=1;
      end;
      run;

proc freq data=fn_codesc;
tables other;
run;


data fn_codesc;
set fn_codesc;
array s[20] $ source1-source20;
hosp=0;
do i=1 to 20;
      if    s[i] in: ('DAD') then hosp=1;
      end;
      run;

data fn_codesc;
set fn_codesc;
array s[20] $ source1-source20;
ed=0;
do i=1 to 20;
      if    s[i] in: ('NACRS') then ed=1;
      end;
      run;

data fn_codesc;
set fn_codesc;
array s[20] $ source1-source20;
pc=0;
do i=1 to 20;
      if    s[i] in: ('OHIP') then pc=1;
      end;
      run;

proc freq data=fn_codesc;
tables hosp ed pc;
run;

proc freq data=fn_codesc;
where interventiontype='HOSPITALIZATION';
tables hosp ed pc notinadmin;
run;

proc freq data=fn_codesc;
where interventiontype='HOSPITALIZATION';
tables c009 c079 c136 c781 c787 other;
run;


proc freq data=fn_codesc;
```

```
where interventiontype='HOSPITALIZATION' and hosp=1;
tables dx10code:;
run;


proc freq data=fn_codesc;
where interventiontype='HOSPITALIZATION';
tables c009 c070 c079 c136 c781 c787 other;
run;

data fn_codesc;
set fn_codesc;
run;


/***********FALSE NEG - LOW SPEC************/


data cohort_wide (rename=(dx10code_1=dx10code1_ dx10code_2=dx10code2_
dx10code_3=dx10code3_));
set cohort;
run;

%MAKEWIDE(data          = cohort_wide,
           out          = dxcode_wide,
           id           = ikn,
           var          = dxcode);

data dxcode_wide;
set dxcode_wide;
run;

%MAKEWIDE(data          = cohort_wide,
           out          = dx10code1_wide,
           id           = ikn,
           var          = dx10code1_);

data dx10code1_wide;
set dx10code1_wide;
run;

%MAKEWIDE(data          = cohort_wide,
           out          = dx10code2_wide,
           id           = ikn,
           var          = dx10code2_);

data dx10code2_wide;
set dx10code2_wide;
run;

%MAKEWIDE(data          = cohort_wide,
           out          = dx10code3_wide,
           id           = ikn,
           var          = dx10code3_);

data dx10code3_wide;
set dx10code3_wide;
```

```
run;


/*now merge together*/
data whole_cohort_wide;
merge
dxcode_wide
dx10code1_wide
dx10code2_wide
dx10code3_wide;
by ikn;
run;


/*now ID those with designated codes*/

/*hep*/

data w1;
set whole_cohort_wide;
array dxc[298] $ dxcode1-dxcode298;
array dx10c1[298] $dx10code1_1-dx10code1_298;
array dx10c2[298] $dx10code2_1-dx10code2_298;
array dx10c3[298] $dx10code3_1-dx10code3_298;
hep=0;
do i=1 to 298;
	if 		dxc[i] in:('070' '009' '079' '136' '787')		then hep=1;
	else if dx10c1[i] in: ('B15' 'b19' 'A09' 'A083' 'A084' 'A085')	then
hep=1;
	else if dx10c2[i] in: ('B15' 'b19' 'A09' 'A083' 'A084' 'A085')	then
hep=1;
	else if dx10c3[i] in: ('B15' 'b19' 'A09' 'A083' 'A084' 'A085')	then
hep=1;
	end;
	run;

proc freq data=w1;
tables hep;
run;


data w1;
set w1;
array dxc[298] $ dxcode1-dxcode298;
array dx10c1[298] $dx10code1_1-dx10code1_298;
array dx10c2[298] $dx10code2_1-dx10code2_298;
array dx10c3[298] $dx10code3_1-dx10code3_298;
mal=0;
do i=1 to 298;
	if 		dxc[i] in:('062' '136' '781' '784' '787') then mal=1;
	else if dx10c1[i] in: ('B5' 'P373' 'P374' 'B64')	then mal=1;
	else if dx10c2[i] in: ('B5' 'P373' 'P374' 'B64')	then mal=1;
	else if dx10c3[i] in: ('B5' 'P373' 'P374' 'B64')	then mal=1;
	end;
	run;

proc freq data=w1;
```

```
tables mal;
run;


data w1;
set w1;
array dxc[298] $ dxcode1-dxcode298;
array dx10c1[298] $dx10code1_1-dx10code1_298;
array dx10c2[298] $dx10code2_1-dx10code2_298;
array dx10c3[298] $dx10code3_1-dx10code3_298;
tpt=0;
do i=1 to 298;
      if    dxc[i] in:('002' '009' '003' '005' '136' '784' '787')
      then tpt=1;
      else if dx10c1[i] in: ('A01' 'A029' 'A021' 'A029' 'A049' 'A059' 'A09'
'A499')     then tpt=1;
      else if dx10c2[i] in: ('A01' 'A029' 'A021' 'A029' 'A049' 'A059' 'A09'
'A499')     then tpt=1;
      else if dx10c3[i] in: ('A01' 'A029' 'A021' 'A029' 'A049' 'A059' 'A09'
'A499')     then tpt=1;
      end;
      run;

proc freq data=w1;
tables tpt;
run;


data w2 (keep=ikn hep mal tpt);
set w1;
run;


/*now merge back with original cohort*/

data cohort_codes;
merge
cohort w2;
by ikn;
run;



/* see which ones are in iPHIS but not in admin */

data cohort_codes;
set cohort_codes;
if          iphis_disease in ("HEPATITIS A") then diseasegp=1;
else if iphis_disease in ("MALARIA") then diseasegp=2;
else if iphis_disease in ("PARATYPHOID FEVER" "TYPHOID FEVER") then
diseasegp=3;
run;


/*hep*/

data hep_fn_codes (where=(diseasegp=1 and hep=0));
```

```
set cohort_codes;
run;

data uniqueikn_hep_fn_codes;
set hep_fn_codes ;
if first.ikn then output;
by ikn;
run;

data uniqueikn_hep_fn_codes;
set uniqueikn_hep_fn_codes;
hep_fn=1;
run;

data h_fn (keep=ikn hep_fn);
set uniqueikn_hep_fn_codes;
run;


/*mal*/

data mal_fn_codes (where=(diseasegp=2 and mal=0));
set cohort_codes;
run;

data uniqueikn_mal_fn_codes;
set mal_fn_codes;
if first.ikn then output;
by ikn;
run;

data uniqueikn_mal_fn_codes;
set uniqueikn_mal_fn_codes;
mal_fn=1;
run;

data m_fn (keep=ikn mal_fn);
set uniqueikn_mal_fn_codes;
run;


/*tpt*/

data tpt_fn_codes (where=(diseasegp=3 and tpt=0));
set cohort_codes;
run;

data uniqueikn_tpt_fn_codes;
set tpt_fn_codes;
if first.ikn then output;
by ikn;
run;

data uniqueikn_tpt_fn_codes;
set uniqueikn_tpt_fn_codes;
tpt_fn=1;
run;
```

```
data t_fn (keep=ikn tpt_fn);
set uniqueikn_tpt_fn_codes;
run;


/*merge all 3 together*/


data fn_lowsp;
merge
h_fn m_fn t_fn;
by ikn;
run;
data fn_mal2 (where=(ikn='X' or ikn='X' or ikn='X'));
set cohort;
run;

data fn_mal2;
set fn_mal2;
if first.ikn then output;
by ikn;
run;

data fn_mal2;
set fn_mal2;
mal_fn=1;
run;

data fn_mal2 (keep=ikn mal_fn);
set fn_mal2;
run;


data fn_tpt2 (where=(ikn='X'));
set cohort;
run;

data fn_tpt2;
set fn_tpt2;
if first.ikn then output;
by ikn;
run;

data fn_tpt2;
set fn_tpt2;
tpt_fn=1;
run;

data fn_tpt2 (keep=ikn tpt_fn);
set fn_tpt2;
run;


data fn_lowsp_complete;
merge
fn_lowsp fn_mal2 fn_tpt2;
```

```
by ikn;
run;

proc freq data=fn_lowsp_complete;
tables hep_fn mal_fn tpt_fn;
run;

data fn_lowsp_complete;
set fn_lowsp_complete;
fn_lowsp=1;
run;

data fn_lowsp_complete (keep=ikn hep_fn mal_fn tpt_fn fn_lowsp);
set fn_lowsp_complete;
run;

proc freq data=fn_lowsp_complete;
tables hep_fn mal_fn tpt_fn;
run;


/*now merge back with refstd and cohort to describe*/

data fn_lowsp;
merge fn_lowsp_complete(in=cohort) refstd (in=ref);
by ikn;
if cohort and ref; /*so that only keeps data for those in cohort*/
run;


/*add back admin vars for all by merging with original cohort (first
record)*/

data fn_lowsp (rename=sex=sexiphis);
set fn_lowsp;
run;

proc sort data=cohort;
by ikn first_admin_code_date;
run;

data uniqueikn;
set cohort;
if first.ikn then count=0;
count + 1;
if last.ikn then output;
by ikn;
run;

data cohort2 (keep=ikn iphis sex age_cohort_entry cic_immigrant cic_fsrce
admin_code_in_lookback elig_at_iphis elig_1yr elig_2yr elig_3yr peel_region);
set uniqueikn;
run;

data cohort_fn_lowsp_final;
merge cohort2 (in=cohort2) fn_lowsp(in=cohort);
by ikn;
```

```
if cohort2 and cohort;
run;


data cohort_fn_lowsp_final;
set cohort_fn_lowsp_final;
agegp=.;
if ageattimeofillness < 18 then agegp=1;
else if ageattimeofillness ge 18 and ageattimeofillness le 64 then agegp=2;
else if ageattimeofillness > 64 then agegp=3;
run;


proc means median min max mean data=cohort_fn_lowsp_final;
var ageattimeofillness;
run;

proc freq data=cohort_fn_lowsp_final;
tables city sexiphis agegp disease countryofbirth travelassociated
interventiontype interventionlocation;
run;

proc freq data=cohort_fn_lowsp_final;
tables cic_immigrant cic_fsrce elig_at_iphis elig_1yr elig_2yr elig_3yr
peel_region admin_code_in_lookback;
run;

/*create perm dataset*/

data DATASETS.final_lsp_fn_02may2017;
set cohort_fn_lowsp_final;
run;

data cohort_fn_lowsp_final;
set DATASETS.final_lsp_fn_02may2017;
run;


/*July 6: Estimate SENS and PPV by immigrant status*/

data hep_fp;
set DATASETS.final_hepa_fp_02may2017;
run;

data hep_fp (where=(h_sp=1));
set hep_fp;
run;

proc freq data=hep_fp;
tables cic_immigrant;
run;

data hep_tp;
set DATASETS.final_hepa_tp_02may2017;
run;

data hep_tp (where=(h_sp=1));
```

```
set hep_tp;
run;

proc freq data=hep_tp;
tables cic_immigrant;
run;



data mal_fp;
set DATASETS.final_mal_fp_02may2017;
run;

data mal_fp (where=(h_sp=1));
set mal_fp;
run;

proc freq data=mal_fp;
tables cic_immigrant;
run;

data mal_tp;
set DATASETS.final_mal_tp_02may2017;
run;

data mal_tp (where=(h_sp=1));
set mal_tp;
run;

proc freq data=mal_tp;
tables cic_immigrant;
run;


data tpt_fp;
set DATASETS.final_tpt_fp_02may2017;
run;

data tpt_fp (where=(h_sp=1));
set tpt_fp;
run;

proc freq data=tpt_fp;
tables cic_immigrant;
run;

data tpt_tp;
set DATASETS.final_tpt_tp_02may2017;
run;

data tpt_tp (where=(h_sp=1));
set tpt_tp;
run;

proc freq data=tpt_tp;
tables cic_immigrant;
run;
```

```
data fn;
set DATASETS.final_hsp_fn_02may2017;
run;

proc freq data=fn;
tables hep_fn mal_fn tpt_fn;
run;

data fn;
set fn;
if hep_fn=1 or disease='HEPATITIS A' then hepa=1;
else if hepa=. then hepa=0;
run;

data fn;
set fn;
if mal_fn=1 or disease='MALARIA' then mal=1;
else if mal=. then mal=0;
run;

data fn;
set fn;
if tpt_fn=1 or disease='TYPHOID FEVER' or disease='PARATYPHOID FEVER' then
tpt=1;
else if tpt=. then tpt=0;
run;

proc freq data=fn;
tables hepa mal tpt;
run;

proc freq data=fn;
tables cic_immigrant*hepa cic_immigrant*mal cic_immigrant*tpt;
run;




/* APRIL 19 - INVESTIGATE FP */


/*1. Sensitivity analysis:  exclude dxpref_1-3=Q (suspect diagnosis)*/


proc freq data=cohort;
tables dxpref:;
run;

data cohort_fp (where=(iphis=0 and first_admin_code_date ne .));
set cohort;
run;
```

```
/*** HEPATITIS A - low sp - FP ***/

data cohort_fp;
set cohort_fp;
hep=0;
      if    dxcode     in: ('070' '009' '079' '136' '787')  then hep=1;
      else if dx10code_1 in: ('B15' 'B19' 'A09' 'A083' 'A084' 'A085') then
hep=1;
      else if dx10code_2 in: ('B15' 'B19' 'A09' 'A083' 'A084' 'A085') then
hep=1;
      else if dx10code_3 in: ('B15' 'B19' 'A09' 'A083' 'A084' 'A085') then
hep=1;
      run;

data cohort_fp_hep (where=(hep=1));
set cohort_fp;
run;

proc freq data=cohort_fp_hep;
tables dxpref:;
run;

proc freq data=cohort_fp_hep;
where dxpref_1='Q';
tables dx10code_1;
run;

proc freq data=cohort_fp_hep;
where dxpref_2='Q';
tables dx10code_2;
run;

proc freq data=cohort_fp_hep;
where dxpref_3='Q';
tables dx10code_3;
run;

/*remove instances where dxpref=Q*/

data cohort_fp_hep;
set cohort_fp_hep;
suspect=.;
if dxpref_1='Q' or dxpref_2='Q' or dxpref_1='Q' then suspect=1;
run;

proc freq data=cohort_fp_hep;
tables suspect;
run; /*3566 obs*/

data cohort_fp_hep_nosus (where=(suspect ne 1));
set cohort_fp_hep;
run;


/*now see how many individuals that removed*/
proc sort data=cohort_fp_hep_nosus;
by ikn first_admin_code_date;
```

```
run;

data uniqueikn_hep_nosus;
set cohort_fp_hep_nosus ;
if first.ikn then count=0;
count + 1;
if last.ikn then output;
by ikn;
run;


/*for high sp*/

data cohort_fp_hep_nosus (rename=(dx10code_1=dx10code1_ dx10code_2=dx10code2_
dx10code_3=dx10code3_));
set cohort_fp_hep_nosus;
run;

%MAKEWIDE(data          = cohort_fp_hep_nosus,
          out           = source_wide,
          id            = ikn,
          var           = source);

data source_wide;
set source_wide;
run;

%MAKEWIDE(data          = cohort_fp_hep_nosus,
          out           = date_wide,
          id            = ikn,
          var           = date);

data date_wide;
set date_wide;
run;

%MAKEWIDE(data          = cohort_fp_hep_nosus,
          out           = dxcode_wide,
          id            = ikn,
          var           = dxcode);

data dxcode_wide;
set dxcode_wide;
run;

%MAKEWIDE(data          = cohort_fp_hep_nosus,
          out           = dx10code1_wide,
          id            = ikn,
          var           = dx10code1_);

data dx10code1_wide;
set dx10code1_wide;
run;

%MAKEWIDE(data          = cohort_fp_hep_nosus,
          out           = dx10code2_wide,
          id            = ikn,
```

```
          var          = dx10code2_);

data dx10code2_wide;
set dx10code2_wide;
run;

%MAKEWIDE(data         = cohort_fp_hep_nosus,
          out          = dx10code3_wide,
          id           = ikn,
          var          = dx10code3_);

data dx10code3_wide;
set dx10code3_wide;
run;


/*now merge together*/
data hepa_wide_fp;
merge source_wide
date_wide
dxcode_wide
dx10code1_wide
dx10code2_wide
dx10code3_wide;
by ikn;
run;


/*1 - highly specificity (h_sp)*/

data ha1;
set hepa_wide_fp;
array dxc[285] $ dxcode1-dxcode285;
array dx10c1[285] $dx10code1_1-dx10code1_285;
array dx10c2[285] $dx10code2_1-dx10code2_285;
array dx10c3[285] $dx10code3_1-dx10code3_285;
h_sp=0;
do i=1 to 285;
      if          dxc[i] in:('070')              then h_sp=1;
      else if dx10c1[i] in: ('B15')       then h_sp=1;
      else if dx10c2[i] in: ('B15')       then h_sp=1;
      else if dx10c3[i] in: ('B15')       then h_sp=1;
      end;
      run;

proc freq data=ha1;
tables h_sp;
run;



/*** HEPATITIS A - low sp - TP ***/

data cohort_tp_hep2;
set cohort_tp;
hep=0;
      if    dxcode  in: ('070' '009' '079' '136' '787') then hep=1;
```

```
      else if dx10code_1 in: ('B15' 'B19' 'A09' 'A083' 'A084' 'A085') then
hep=1;
      else if dx10code_2 in: ('B15' 'B19' 'A09' 'A083' 'A084' 'A085') then
hep=1;
      else if dx10code_3 in: ('B15' 'B19' 'A09' 'A083' 'A084' 'A085') then
hep=1;
      run;

data cohort_tp_hep2 (where=(diseasegp=1 and hep=1));
set cohort_tp_hep2;
run;


/*how many unique individuals?*/

proc sort data=cohort_tp_hep2;
by ikn first_admin_code_date;
run;

data uniqueikn_hep;
set cohort_tp_hep2;
if first.ikn then count=0;
count + 1;
if last.ikn then output;
by ikn;
run;


/*Look at time between iPHIS episode date (onset) and health encounter dates
in admin data*/

data cohort_tp_hep2;
set cohort_tp_hep2;
datediffiphis=date - iphis_accurateepisodedate;
run;

data cohort_tp_hep2;
set cohort_tp_hep2;
if    datediffiphis < -40 then iphisdategp2=1;
else if datediffiphis ge -40 and datediffiphis le 120 then iphisdategp2=2;
else if datediffiphis > 120   then iphisdategp2=3;
run;


/*Apply rule:  IF codes 787 or 079 or 009 or A099 AND iphisdategp2 = 1 or 3,
exclude*/

data cohort_tp_hep3;
set cohort_tp_hep2;
fn=0;
if    dxcode in: ('787' '079' '009') and (iphisdategp2 ne 2)      then fn=1;
else if dx10code_1 in: ('A099') and (iphisdategp2 ne 2)           then fn=1;
run;

proc freq data=cohort_tp_hep3;
tables fn;
run;
```

```
data cohort_tp_hep4 (where=(fn ne 1));
set cohort_tp_hep3;
run;

proc freq data=cohort_tp_hep4;
where dxpref_1='Q';
tables dx10code_1;
run;

proc freq data=cohort_tp_hep4;
where dxpref_2='Q';
tables dx10code_2;
run;

proc freq data=cohort_tp_hep4;
where dxpref_3='Q';
tables dx10code_3;
run;

/*remove instances where dxpref=Q*/

data cohort_tp_hep4;
set cohort_tp_hep4;
suspect=.;
if dxpref_1='Q' or dxpref_2='Q' or dxpref_1='Q' then suspect=1;
run;

proc freq data=cohort_tp_hep4;
tables suspect;
run;

data cohort_tp_hep_nosus (where=(suspect ne 1));
set cohort_tp_hep4;
run;


/*how many unique individuals?*/

proc sort data=cohort_tp_hep_nosus;
by ikn first_admin_code_date;
run;

data uniqueikn_hep;
set cohort_tp_hep_nosus;
if first.ikn then count=0;
count + 1;
if last.ikn then output;
by ikn;
run;


/*FOR HIGH SP*/

data cohort_tp_hep_nosus (rename=(dx10code_1=dx10code1_ dx10code_2=dx10code2_
dx10code_3=dx10code3_));
set cohort_tp_hep_nosus;
```

```
run;

%MAKEWIDE(data          = cohort_tp_hep_nosus,
          out           = source_wide,
          id            = ikn,
          var           = source);

data source_wide;
set source_wide;
run;

%MAKEWIDE(data          = cohort_tp_hep_nosus,
          out           = date_wide,
          id            = ikn,
          var           = date);

data date_wide;
set date_wide;
run;

%MAKEWIDE(data          = cohort_tp_hep_nosus,
          out           = dxcode_wide,
          id            = ikn,
          var           = dxcode);

data dxcode_wide;
set dxcode_wide;
run;

%MAKEWIDE(data          = cohort_tp_hep_nosus,
          out           = dx10code1_wide,
          id            = ikn,
          var           = dx10code1_);

data dx10code1_wide;
set dx10code1_wide;
run;

%MAKEWIDE(data          = cohort_tp_hep_nosus,
          out           = dx10code2_wide,
          id            = ikn,
          var           = dx10code2_);

data dx10code2_wide;
set dx10code2_wide;
run;

%MAKEWIDE(data          = cohort_tp_hep_nosus,
          out           = dx10code3_wide,
          id            = ikn,
          var           = dx10code3_);

data dx10code3_wide;
set dx10code3_wide;
run;
```

```
/*now merge together*/
data hepa_wide2;
merge source_wide
date_wide
dxcode_wide
dx10code1_wide
dx10code2_wide
dx10code3_wide;
by ikn;
run;


/*Look at constellation*/

/*high*/
data ha1;
set hepa_wide2;
array dxc[15] $ dxcode1-dxcode15;
array dx10c1[15] $dx10code1_1-dx10code1_15;
array dx10c2[15] $dx10code2_1-dx10code2_15;
array dx10c3[15] $dx10code3_1-dx10code3_15;
h_sp=0;
do i=1 to 15;
      if          dxc[i] in:('070')       then h_sp=1;
      else if dx10c1[i] in: ('B15')       then h_sp=1;
      else if dx10c2[i] in: ('B15')       then h_sp=1;
      else if dx10c3[i] in: ('B15')       then h_sp=1;
      end;
      run;

proc freq data=ha1;
tables h_sp;
run;


/***** MALARIA - low sp - FP *****/

data cohort_fp;
set cohort_fp;
mal=0;
      if          dxcode     in: ('062' '136' '781' '784' '787')  then mal=1;
      else if dx10code_1 in: ('B5' 'P373' 'P374' 'B64')          then mal=1;
      else if dx10code_2 in: ('B5' 'P373' 'P374' 'B64')          then mal=1;
      else if dx10code_3 in: ('B5' 'P373' 'P374' 'B64')          then mal=1;
      run;

data cohort_fp_mal (where=(mal=1));
set cohort_fp;
run;

proc freq data=cohort_fp_mal;
where dxpref_1='Q';
tables dx10code_1;
run;

proc freq data=cohort_fp_mal;
where dxpref_2='Q';
```

```
tables dx10code_2;
run;

proc freq data=cohort_fp_mal;
where dxpref_3='Q';
tables dx10code_3;
run;

/*remove instances where dxpref=Q*/

data cohort_fp_mal;
set cohort_fp_mal;
suspect=.;
if dxpref_1='Q' or dxpref_2='Q' or dxpref_1='Q' then suspect=1;
run;

proc freq data=cohort_fp_mal;
tables suspect;
run; /*31 obs*/

data cohort_fp_mal_nosus (where=(suspect ne 1));
set cohort_fp_mal;
run;


/*how many unique individuals?*/

proc sort data=cohort_fp_mal_nosus;
by ikn first_admin_code_date;
run;

data uniqueikn_mal;
set cohort_fp_mal_nosus;
if first.ikn then count=0;
count + 1;
if last.ikn then output;
by ikn;
run;


/* for high sp */

data cohort_fp_mal_nosus (rename=(dx10code_1=dx10code1_ dx10code_2=dx10code2_
dx10code_3=dx10code3_));
set cohort_fp_mal_nosus;
run;

%MAKEWIDE(data          = cohort_fp_mal_nosus,
           out          = source_wide,
           id           = ikn,
           var          = source);

data source_wide;
set source_wide;
run;

%MAKEWIDE(data          = cohort_fp_mal_nosus,
```

```
           out          = date_wide,
           id           = ikn,
           var          = date);

data date_wide;
set date_wide;
run;

%MAKEWIDE(data          = cohort_fp_mal_nosus,
           out          = dxcode_wide,
           id           = ikn,
           var          = dxcode);

data dxcode_wide;
set dxcode_wide;
run;

%MAKEWIDE(data          = cohort_fp_mal_nosus,
           out          = dx10code1_wide,
           id           = ikn,
           var          = dx10code1_);

data dx10code1_wide;
set dx10code1_wide;
run;

%MAKEWIDE(data          = cohort_fp_mal_nosus,
           out          = dx10code2_wide,
           id           = ikn,
           var          = dx10code2_);

data dx10code2_wide;
set dx10code2_wide;
run;

%MAKEWIDE(data          = cohort_fp_mal_nosus,
           out          = dx10code3_wide,
           id           = ikn,
           var          = dx10code3_);

data dx10code3_wide;
set dx10code3_wide;
run;


/*now merge together*/
data mal_wide_fp;
merge source_wide
date_wide
dxcode_wide
dx10code1_wide
dx10code2_wide
dx10code3_wide;
by ikn;
run;
```

```
/*1 - highly specificity (h_sp)*/

data m1;
set mal_wide_fp;
array dxc[298] $ dxcode1-dxcode298;
array dx10c1[298] $dx10code1_1-dx10code1_298;
array dx10c2[298] $dx10code2_1-dx10code2_298;
array dx10c3[298] $dx10code3_1-dx10code3_298;
h_sp=0;
do i=1 to 298;
      if          dxc[i] in:('062')             then h_sp=1;
      else if dx10c1[i] in: ('B5')  then h_sp=1;
      else if dx10c2[i] in: ('B5')  then h_sp=1;
      else if dx10c3[i] in: ('B5')  then h_sp=1;
      end;
      run;

proc freq data=m1;
tables h_sp;
run;

/***** MALARIA - low sp - TP *****/

data cohort_tp_mal2;
set cohort_tp;
mal=0;
      if          dxcode     in: ('062' '136' '781' '784' '787')  then mal=1;
      else if dx10code_1 in: ('B5' 'P373' 'P374' 'B64')           then mal=1;
      else if dx10code_2 in: ('B5' 'P373' 'P374' 'B64')           then mal=1;
      else if dx10code_3 in: ('B5' 'P373' 'P374' 'B64')           then mal=1;
      run;

data cohort_tp_mal2 (where=(diseasegp=2 and mal=1));
set cohort_tp_mal2;
run;


/*Look at time between iPHIS episode date (onset) and health encounter dates
in admin data*/

data cohort_tp_mal2;
set cohort_tp_mal2;
datediffiphis=date - iphis_accurateepisodedate;
run;

data cohort_tp_mal2;
set cohort_tp_mal2;
if    datediffiphis < -30     then iphisdategp2=1;
else if datediffiphis ge -30 and datediffiphis le 120 then iphisdategp2=2;
else if datediffiphis > 120   then iphisdategp2=3;
run;

/*Apply rule:  IF codes 781, 787 or 136 AND iphisdategp2 = 1 or 3, exclude*/

data cohort_tp_mal3;
set cohort_tp_mal2;
fn=0;
```

```
if dxcode in: ('136' '781' '787') and (iphisdategp2 ne 2)   then fn=1;
run;

data cohort_tp_mal4 (where=(fn ne 1));
set cohort_tp_mal3;
run;

proc freq data=cohort_tp_mal4;
where dxpref_1='Q';
tables dx10code_1;
run;

proc freq data=cohort_tp_mal4;
where dxpref_2='Q';
tables dx10code_2;
run;

proc freq data=cohort_tp_mal4;
where dxpref_3='Q';
tables dx10code_3;
run;

/*remove instances where dxpref=Q*/

data cohort_tp_mal4;
set cohort_tp_mal4;
suspect=.;
if dxpref_1='Q' or dxpref_2='Q' or dxpref_1='Q' then suspect=1;
run;

proc freq data=cohort_tp_mal4;
tables suspect;
run;

data cohort_tp_mal_nosus (where=(suspect ne 1));
set cohort_tp_mal4;
run;

/*how many unique individuals?*/

proc sort data=cohort_tp_mal_nosus;
by ikn first_admin_code_date;
run;

data uniqueikn_mal;
set cohort_tp_mal_nosus;
if first.ikn then count=0;
count + 1;
if last.ikn then output;
by ikn;
run;

/*high sp*/

data cohort_tp_mal4 (rename=(dx10code_1=dx10code1_ dx10code_2=dx10code2_
dx10code_3=dx10code3_));
set cohort_tp_mal_nosus;
```

```
run;

%MAKEWIDE(data          = cohort_tp_mal4,
          out           = source_wide,
          id            = ikn,
          var           = source);

data source_wide;
set source_wide;
run;

%MAKEWIDE(data          = cohort_tp_mal4,
          out           = date_wide,
          id            = ikn,
          var           = date);

data date_wide;
set date_wide;
run;

%MAKEWIDE(data          = cohort_tp_mal4,
          out           = dxcode_wide,
          id            = ikn,
          var           = dxcode);

data dxcode_wide;
set dxcode_wide;
run;

%MAKEWIDE(data          = cohort_tp_mal4,
          out           = dx10code1_wide,
          id            = ikn,
          var           = dx10code1_);

data dx10code1_wide;
set dx10code1_wide;
run;

%MAKEWIDE(data          = cohort_tp_mal4,
          out           = dx10code2_wide,
          id            = ikn,
          var           = dx10code2_);

data dx10code2_wide;
set dx10code2_wide;
run;

%MAKEWIDE(data          = cohort_tp_mal4,
          out           = dx10code3_wide,
          id            = ikn,
          var           = dx10code3_);

data dx10code3_wide;
set dx10code3_wide;
run;
```

```
/*now merge together*/
data mal_wide4;
merge source_wide
date_wide
dxcode_wide
dx10code1_wide
dx10code2_wide
dx10code3_wide;
by ikn;
run;


/*Look at constellation*/

data m1;
set mal_wide4;
array dxc[37] $ dxcode1-dxcode37;
array dx10c1[37] $dx10code1_1-dx10code1_37;
array dx10c2[37] $dx10code2_1-dx10code2_37;
array dx10c3[37] $dx10code3_1-dx10code3_37;
h_sp=0;
do i=1 to 37;
      if          dxc[i] in: ('062')            then h_sp=1;
      else if dx10c1[i] in: ('B5')  then h_sp=1;
      else if dx10c2[i] in: ('B5')  then h_sp=1;
      else if dx10c3[i] in: ('B5')  then h_sp=1;
      end;
      run;

proc freq data=m1;
tables h_sp;
run;


/***** T/PT - low sp - FP *****/

data cohort_fp;
set cohort_fp;
tpt=0;
      if dxcode in: ('002' '009' '003' '005' '136' '784' '787') then tpt=1;
      else if dx10code_1 in: ('A01' 'A029' 'A021' 'A049' 'A059' 'A09' 'A499')
then tpt=1;
      else if dx10code_2 in: ('A01' 'A029' 'A021' 'A049' 'A059' 'A09' 'A499')
then tpt=1;
      else if dx10code_3 in: ('A01' 'A029' 'A021' 'A049' 'A059' 'A09' 'A499')
then tpt=1;
      run;

data cohort_fp_tpt (where=(tpt=1));
set cohort_fp;
run;

proc freq data=cohort_fp_tpt;
where dxpref_1='Q';
tables dx10code_1;
run;
```

```
proc freq data=cohort_fp_tpt;
where dxpref_2='Q';
tables dx10code_2;
run;

proc freq data=cohort_fp_tpt;
where dxpref_3='Q';
tables dx10code_3;
run;

/*remove instances where dxpref=Q*/

data cohort_fp_tpt;
set cohort_fp_tpt;
suspect=.;
if dxpref_1='Q' or dxpref_2='Q' or dxpref_1='Q' then suspect=1;
run;

proc freq data=cohort_fp_tpt;
tables suspect;
run;

data cohort_fp_tpt_nosus (where=(suspect ne 1));
set cohort_fp_tpt;
run;


/*how many unique individuals?*/

proc sort data=cohort_fp_tpt_nosus;
by ikn first_admin_code_date;
run;

data uniqueikn_tpt;
set cohort_fp_tpt_nosus;
if first.ikn then count=0;
count + 1;
if last.ikn then output;
by ikn;
run;

/*high sp*/

data cohort_fp_tpt (rename=(dx10code_1=dx10code1_ dx10code_2=dx10code2_
dx10code_3=dx10code3_));
set cohort_fp_tpt_nosus;
run;


/*need to flatten only one row per ikn/individual level*/

%MAKEWIDE(data          = cohort_fp_tpt,
           out          = source_wide,
           id           = ikn,
           var          = source);

data source_wide;
```

```
set source_wide;
run;

%MAKEWIDE(data          = cohort_fp_tpt,
          out           = date_wide,
          id            = ikn,
          var           = date);

data date_wide;
set date_wide;
run;

%MAKEWIDE(data          = cohort_fp_tpt,
          out           = dxcode_wide,
          id            = ikn,
          var           = dxcode);

data dxcode_wide;
set dxcode_wide;
run;

%MAKEWIDE(data          = cohort_fp_tpt,
          out           = dx10code1_wide,
          id            = ikn,
          var           = dx10code1_);

data dx10code1_wide;
set dx10code1_wide;
run;

%MAKEWIDE(data          = cohort_fp_tpt,
          out           = dx10code2_wide,
          id            = ikn,
          var           = dx10code2_);

data dx10code2_wide;
set dx10code2_wide;
run;

%MAKEWIDE(data          = cohort_fp_tpt,
          out           = dx10code3_wide,
          id            = ikn,
          var           = dx10code3_);

data dx10code3_wide;
set dx10code3_wide;
run;


/*now merge together*/
data tpt_wide_fp;
merge source_wide
date_wide
dxcode_wide
dx10code1_wide
dx10code2_wide
dx10code3_wide;
```

```
by ikn;
run;

/*1 - high_sp*/

data t1;
set tpt_wide_fp;
array dxc[285] $ dxcode1-dxcode285;
array dx10c1[285] $dx10code1_1-dx10code1_285;
array dx10c2[285] $dx10code2_1-dx10code2_285;
array dx10c3[285] $dx10code3_1-dx10code3_285;
h_sp=0;
do i=1 to 285;
      if          dxc[i] in: ('002')      then h_sp=1;
      else if dx10c1[i] in: ('A01')       then h_sp=1;
      else if dx10c2[i] in: ('A01')       then h_sp=1;
      else if dx10c3[i] in: ('A01')       then h_sp=1;
      end;
      run;

proc freq data=t1;
tables h_sp;
run;

/***** T/PT - low sp - TP *****/

data cohort_tp_tpt2;
set cohort_tp;
tpt=0;
      if          dxcode     in: ('002' '009' '003' '005' '136' '784' '787')
      then tpt=1;
      else if dx10code_1 in: ('A01' 'A029' 'A021' 'A049' 'A059' 'A09' 'A499')
then tpt=1;
      else if dx10code_2 in: ('A01' 'A029' 'A021' 'A049' 'A059' 'A09' 'A499')
then tpt=1;
      else if dx10code_3 in: ('A01' 'A029' 'A021' 'A049' 'A059' 'A09' 'A499')
then tpt=1;
      run;

data cohort_tp_tpt2 (where=(diseasegp=3 and tpt=1));
set cohort_tp_tpt2;
run;


/*need to correct for two cases with two distinct disease episodes */

data duplicates;
set cohort_tp_tpt2;
dup=0;
if ikn=X or ikn=X then dup=1;
run;

data duplicates (where=(dup=1));
set duplicates;
run;
```

```
data duplicates (drop=iphis_accurateepisodedate iphis_disease
iphis_countryofbirth iphis_travelassociated iphis_traveldestination1);
set duplicates;
run;

data duplicates;
set duplicates;
length rec $20;
rec='rec2';
run;

data duplicates;
set duplicates;
length ikn2 $20;
ikn2=catx("_", of ikn rec);
run;

data duplicates (drop=ikn dup _a rec);
set duplicates;
run;

data duplicates;
rename ikn2=ikn iphis_accurateepisodedate_rec2=iphis_accurateepisodedate
iphis_disease_rec2=iphis_disease
iphis_countryofbirth_rec2=iphis_countryofbirth
iphis_travelassociated_rec2=iphis_travelassociated
iphis_traveldestination1_rec2=iphis_traveldestination1;
set duplicates;
run;

data duplicates;
set duplicates;
run;

/*now append to cohort of TP*/

data cohort_tp_tpt3 (drop=iphis_accurateepisodedate_rec2 iphis_disease_rec2
iphis_countryofbirth_rec2 iphis_travelassociated_rec2
iphis_traveldestination1_rec2);
set cohort_tp_tpt2;
run;

data cohort_tp_tpt3;
set cohort_tp_tpt3;
length ikn2 $20;
ikn2=ikn;
run;

data cohort_tp_tpt3 (drop=ikn);
set cohort_tp_tpt3;
run;

data cohort_tp_tpt3 (rename=ikn2=ikn);
set cohort_tp_tpt3;
run;

proc append base=cohort_tp_tpt3 data=duplicates;
```

```
run;

data cohort_tp_tpt_append;
set cohort_tp_tpt3;
run;


data cohort_tp_tpt_append;
retain ikn iphis iphis_accurateepisodedate iphis_disease iphis_countryofbirth
iphis_travelassociated iphis_traveldestination1
first_admin_code_date cohort_entry age_cohort_entry sex cic_immigrant
cic_fsrce   admin_code_in_lookback elig_at_iphis elig_1yr
elig_2yr elig_3yr source date ddate dxcode dx10code_1 dxtype_1 dxpref_1
      dx10code_2 dxtype_2     dxpref_2 dx10code_3     dxtype_3 dxpref_3
peel_region age   diseasegp tpt;
set cohort_tp_tpt3;
run;

data cohort_tp_tpt_append;
set cohort_tp_tpt_append;
run;


/*how many unique individuals?*/

proc sort data=cohort_tp_tpt_append;
by ikn first_admin_code_date;
run;

data uniqueikn_tpt;
set cohort_tp_tpt_append;
if first.ikn then count=0;
count + 1;
if last.ikn then output;
by ikn;
run;


/*Look at time between iPHIS episode date (onset) and health encounter dates
in admin data*/

data cohort_tp_tpt2;
set cohort_tp_tpt_append;
datediffiphis=date - iphis_accurateepisodedate;
run;

data cohort_tp_tpt2;
set cohort_tp_tpt2;
if          datediffiphis < -30     then iphisdategp3=1;
else if datediffiphis ge -30 and datediffiphis le 200 then iphisdategp3=2;
else if datediffiphis > 200         then iphisdategp3=3;
run;

/*Apply rule:  IF codes 787 or 009 or 136 or A099 AND iphisdategp3 = 1 or 3,
exclude*/

data cohort_tp_tpt3;
```

```
set cohort_tp_tpt2;
fn=0;
if dxcode in: ('009' '787' '136') and (iphisdategp3 ne 2)   then fn=1;
else if dx10code_1 in: ('A099') and (iphisdategp3 ne 2)     then fn=1;
run;

proc freq data=cohort_tp_tpt3;
tables fn;
run;

proc sort data=cohort_tp_tpt3;
by fn ikn;
run;

data cohort_tp_tpt4 (where=(fn ne 1));
set cohort_tp_tpt3;
run;

proc freq data=cohort_tp_tpt4;
where dxpref_1='Q';
tables dx10code_1;
run;

proc freq data=cohort_tp_tpt4;
where dxpref_2='Q';
tables dx10code_2;
run;

proc freq data=cohort_tp_tpt4;
where dxpref_3='Q';
tables dx10code_3;
run;

/*remove instances where dxpref=Q*/

data cohort_tp_tpt4;
set cohort_tp_tpt4;
suspect=.;
if dxpref_1='Q' or dxpref_2='Q' or dxpref_1='Q' then suspect=1;
run;

proc freq data=cohort_tp_tpt4;
tables suspect;
run;

data cohort_tp_tpt_nosus (where=(suspect ne 1));
set cohort_tp_tpt4;
run;


/*how many unique individuals?*/

proc sort data=cohort_tp_tpt_nosus;
by ikn first_admin_code_date;
run;

data uniqueikn_tpt;
```

```
set cohort_tp_tpt_nosus;
if first.ikn then count=0;
count + 1;
if last.ikn then output;
by ikn;
run;

/*high sp*/
data cohort_tp_tpt4 (rename=(dx10code_1=dx10code1_ dx10code_2=dx10code2_
dx10code_3=dx10code3_));
set cohort_tp_tpt_nosus;
run;

%MAKEWIDE(data          = cohort_tp_tpt4,
           out          = source_wide,
           id           = ikn,
           var          = source);

data source_wide;
set source_wide;
run;

%MAKEWIDE(data          = cohort_tp_tpt4,
           out          = date_wide,
           id           = ikn,
           var          = date);

data date_wide;
set date_wide;
run;

%MAKEWIDE(data          = cohort_tp_tpt4,
           out          = dxcode_wide,
           id           = ikn,
           var          = dxcode);

data dxcode_wide;
set dxcode_wide;
run;

%MAKEWIDE(data          = cohort_tp_tpt4,
           out          = dx10code1_wide,
           id           = ikn,
           var          = dx10code1_);

data dx10code1_wide;
set dx10code1_wide;
run;

%MAKEWIDE(data          = cohort_tp_tpt4,
           out          = dx10code2_wide,
           id           = ikn,
           var          = dx10code2_);

data dx10code2_wide;
set dx10code2_wide;
run;
```

```
%MAKEWIDE(data          = cohort_tp_tpt4,
           out           = dx10code3_wide,
           id            = ikn,
           var           = dx10code3_);

data dx10code3_wide;
set dx10code3_wide;
run;

/*now merge together*/
data tpt_wide2;
merge source_wide
date_wide
dxcode_wide
dx10code1_wide
dx10code2_wide
dx10code3_wide;
by ikn;
run;


/*Look at constellation*/

data t1;
set tpt_wide2;
array dxc[25] $ dxcode1-dxcode25;
array dx10c1[25] $dx10code1_1-dx10code1_25;
array dx10c2[25] $dx10code2_1-dx10code2_25;
array dx10c3[25] $dx10code3_1-dx10code3_25;
h_sp=0;
do i=1 to 25;
      if          dxc[i] in: ('002')            then h_sp=1;
      else if dx10c1[i] in: ('A01')       then h_sp=1;
      else if dx10c2[i] in: ('A01')       then h_sp=1;
      else if dx10c3[i] in: ('A01')       then h_sp=1;
      end;
      run;

proc freq data=t1;
tables h_sp;
run;

/**no material impact on sens or PPV**/



/*2. Sensitivity analysis:  flag and exclude patients with a G583 or G589
feecode 0-60 days prior to diagnostic code*/

data immunize;
set MYDATA.g53_feecodes_02may2017;
run;

proc freq data=immunize;
tables feecode dxcode;
run;
```

```
/*how many unique individuals?*/
proc sort data=immunize;
by ikn servdate;
run;

data uniqueikn;
set immunize;
if first.ikn then count=0;
count + 1;
if last.ikn then output;
by ikn;
run;

proc means min max data=uniqueikn;
var count;
run;

data immunize (rename=(dxcode=dxcodei));
set immunize;
run;

proc freq data=immunize;
tables feecode dxcodei;
run;

/*create flag if dxcodei=002 or 070 or 136*/
data flagcode;
set immunize;
flagdxcode=0;
if dxcodei in: ('002' '070' '136') then flagdxcode=1;
run;

proc freq data=flagcode;
tables flagdxcode;
run;

data flagcode (where=(flagdxcode=1));
set flagcode;
run;

data flagcode (keep=ikn flagdxcode);
set flagcode;
run;

proc sort data=flagcode
out=flagcode nodupkey;
by ikn flagdxcode;
run;

/*Check for duplicate IKNs*/

proc freq data=flagcode;
tables ikn/noprint out=keylist;
run;
proc print;
where count ge 2;
```

```
run;


/*merge with cohort - need to do a Cartesian product between matching vars*/

proc sql;
create table both as
      select cohort.ikn
      ,cohort.dxcode
      ,cohort.date
      ,immunize.dxcodei
      ,immunize.servdate
from cohort
      ,immunize
where cohort.ikn=immunize.ikn;
quit;

data both;
set both;
run;

/*flag if servdate 0-60 days prior to date*/

data g538;
set both;
datediff=servdate-date;
run;

data g538;
set g538;
flag60=0;
if datediff le 0 and datediff ge -60 then flag60=1;
run;

data g538;
set g538;
flagsameday=0;
if datediff = 0 then flagsameday=1;
run;

proc freq data=g538;
tables flag60 flagsameday;
run;

data g538;
set g538;
run;


/*create two smaller data sets to merge with original cohort*/

data flag60 (where=(flag60=1));
set g538;
run;

data flag60 (keep=ikn flag60);
set flag60;
```

```
run;

proc sort data=flag60
out=flag60 nodupkey;
by ikn flag60;
run;

/*Check for duplicate IKNs*/

proc freq data=flag60;
tables ikn/noprint out=keylist;
run;
proc print;
where count ge 2;
run;


data flag0 (where=(flagsameday=1));
set g538;
run;

data flag0 (keep=ikn flagsameday);
set flag0;
run;

proc sort data=flag0
out=flag0 nodupkey;
by ikn flagsameday;
run;

/*Check for duplicate IKNs*/

proc freq data=flag0;
tables ikn/noprint out=keylist;
run;
proc print;
where count ge 2;
run;

/*now merge with original cohort and re-do analysis to exclude TP and FP with
a flag*/

proc sort data=cohort;
by ikn first_admin_code_date date;
run;

data cohort_flag;
merge cohort (in=cohort) flag60 (in=flag) flag0(in=flag2) flagcode(in=flag3);
by ikn;
if cohort; /*keeps all in cohort*/
run;

proc freq data=cohort_flag;
tables flag60 flagsameday flagdxcode;
run;

/*down to patient-level to check #s*/
```

```
proc sort data=cohort_flag;
by ikn servdate;
run;

data uniqueikn_flag;
set cohort_flag;
if first.ikn then count=0;
count + 1;
if last.ikn then output;
by ikn;
run;

proc freq data=uniqueikn_flag;
tables flag60 flagsameday flagdxcode;
run; /*checks*/

data cohort_flag;
set cohort_flag;
run;


data cohort_flag;
set cohort_flag;
flag02=0;
if flagsameday=1 or flagdxcode=1 then flag02=1;
run;

proc freq data=cohort_flag;
tables flag02 flagsameday*flagdxcode;
run;

data cohort_flag;
set cohort_flag;
run;

proc sort data=cohort_flag;
by flag02;
run;


/*re-run analysis for sens/PPV calcs - LOW SP first by disease, and exclude
flags*/


data cohort_fp (where=(iphis=0 and first_admin_code_date ne .));
set cohort_flag;
run;

data cohort_tp (where=(iphis=1 and first_admin_code_date ne .));
set cohort_flag;
run;


data cohort_tp;
set cohort_tp;
if    iphis_disease in ("HEPATITIS A")    then diseasegp=1;
else if iphis_disease in ("MALARIA")      then diseasegp=2;
```

```
else if iphis_disease in ("PARATYPHOID FEVER" "TYPHOID FEVER") then
diseasegp=3;
run;


/*** HEPATITIS A - low sp - FP ***/

data cohort_fp;
set cohort_fp;
hep=0;
      if    dxcode in: ('070' '009' '079' '136' '787')            then hep=1;
      else if dx10code_1 in: ('B15' 'B19' 'A09' 'A083' 'A084' 'A085') then
hep=1;
      else if dx10code_2 in: ('B15' 'B19' 'A09' 'A083' 'A084' 'A085') then
hep=1;
      else if dx10code_3 in: ('B15' 'B19' 'A09' 'A083' 'A084' 'A085') then
hep=1;
      run;

data cohort_fp_hep (where=(hep=1));
set cohort_fp;
run;

proc sort data=cohort_fp_hep;
by ikn first_admin_code_date;
run;

data uniqueikn_hep;
set cohort_fp_hep;
if first.ikn then count=0;
count + 1;
if last.ikn then output;
by ikn;
run;

/*remove instances where flag60=1*/

data cohort_fp_hep_flag60 (where=(flag60 ne 1));
set cohort_fp_hep;
run;


/*now see how many individuals that removed*/
proc sort data=cohort_fp_hep_flag60;
by ikn first_admin_code_date;
run;

data uniqueikn_hep_flag60;
set cohort_fp_hep_flag60;
if first.ikn then count=0;
count + 1;
if last.ikn then output;
by ikn;
run;


/*high sp*/
```

```
data cohort_fp_hep_flag60 (rename=(dx10code_1=dx10code1_
dx10code_2=dx10code2_ dx10code_3=dx10code3_));
set cohort_fp_hep_flag60;
run;

%MAKEWIDE(data          = cohort_fp_hep_flag60,
          out           = source_wide,
          id            = ikn,
          var           = source);

data source_wide;
set source_wide;
run;

%MAKEWIDE(data          = cohort_fp_hep_flag60,
          out           = date_wide,
          id            = ikn,
          var           = date);

data date_wide;
set date_wide;
run;

%MAKEWIDE(data          = cohort_fp_hep_flag60,
          out           = dxcode_wide,
          id            = ikn,
          var           = dxcode);

data dxcode_wide;
set dxcode_wide;
run;

%MAKEWIDE(data          = cohort_fp_hep_flag60,
          out           = dx10code1_wide,
          id            = ikn,
          var           = dx10code1_);

data dx10code1_wide;
set dx10code1_wide;
run;

%MAKEWIDE(data          = cohort_fp_hep_flag60,
          out           = dx10code2_wide,
          id            = ikn,
          var           = dx10code2_);

data dx10code2_wide;
set dx10code2_wide;
run;

%MAKEWIDE(data          = cohort_fp_hep_flag60,
          out           = dx10code3_wide,
          id            = ikn,
          var           = dx10code3_);

data dx10code3_wide;
```

```
set dx10code3_wide;
run;


/*now merge together*/
data hepa_wide_fp_flag60;
merge source_wide
date_wide
dxcode_wide
dx10code1_wide
dx10code2_wide
dx10code3_wide;
by ikn;
run;


/*1 - highly specificity (h_sp)*/

data ha1;
set hepa_wide_fp_flag60;
array dxc[285] $ dxcode1-dxcode285;
array dx10c1[285] $dx10code1_1-dx10code1_285;
array dx10c2[285] $dx10code2_1-dx10code2_285;
array dx10c3[285] $dx10code3_1-dx10code3_285;
h_sp=0;
do i=1 to 285;
      if           dxc[i] in:('070')       then h_sp=1;
      else if dx10c1[i] in: ('B15')       then h_sp=1;
      else if dx10c2[i] in: ('B15')       then h_sp=1;
      else if dx10c3[i] in: ('B15')       then h_sp=1;
      end;
      run;

proc freq data=ha1;
tables h_sp;
run;



/*remove instances where flagsameday=1 or flagcode=1*/

data cohort_fp_hep_flag0 (where=(flag02 ne 1));
set cohort_fp_hep;
run;


/*now see how many individuals that removed*/
proc sort data=cohort_fp_hep_flag0;
by ikn first_admin_code_date;
run;

data uniqueikn_hep_flag0;
set cohort_fp_hep_flag0;
if first.ikn then count=0;
count + 1;
if last.ikn then output;
by ikn;
```

```
run;

/*high sp*/

data cohort_fp_hep_flag0 (rename=(dx10code_1=dx10code1_ dx10code_2=dx10code2_
dx10code_3=dx10code3_));
set cohort_fp_hep_flag0;
run;

%MAKEWIDE(data          = cohort_fp_hep_flag0,
          out           = source_wide,
          id            = ikn,
          var           = source);

data source_wide;
set source_wide;
run;

%MAKEWIDE(data          = cohort_fp_hep_flag0,
          out           = date_wide,
          id            = ikn,
          var           = date);

data date_wide;
set date_wide;
run;

%MAKEWIDE(data          = cohort_fp_hep_flag0,
          out           = dxcode_wide,
          id            = ikn,
          var           = dxcode);

data dxcode_wide;
set dxcode_wide;
run;

%MAKEWIDE(data          = cohort_fp_hep_flag0,
          out           = dx10code1_wide,
          id            = ikn,
          var           = dx10code1_);

data dx10code1_wide;
set dx10code1_wide;
run;

%MAKEWIDE(data          = cohort_fp_hep_flag0,
          out           = dx10code2_wide,
          id            = ikn,
          var           = dx10code2_);

data dx10code2_wide;
set dx10code2_wide;
run;

%MAKEWIDE(data          = cohort_fp_hep_flag0,
          out           = dx10code3_wide,
```

```
            id          = ikn,
            var         = dx10code3_);

data dx10code3_wide;
set dx10code3_wide;
run;


/*now merge together*/
data hepa_wide_fp_flag0;
merge source_wide
date_wide
dxcode_wide
dx10code1_wide
dx10code2_wide
dx10code3_wide;
by ikn;
run;


/*1 - highly specificity (h_sp)*/

data ha1;
set hepa_wide_fp_flag0;
array dxc[285] $ dxcode1-dxcode285;
array dx10c1[285] $dx10code1_1-dx10code1_285;
array dx10c2[285] $dx10code2_1-dx10code2_285;
array dx10c3[285] $dx10code3_1-dx10code3_285;
h_sp=0;
do i=1 to 285;
      if          dxc[i] in:('070')            then h_sp=1;
      else if dx10c1[i] in: ('B15')       then h_sp=1;
      else if dx10c2[i] in: ('B15')       then h_sp=1;
      else if dx10c3[i] in: ('B15')       then h_sp=1;
      end;
      run;

proc freq data=ha1;
tables h_sp;
run;


/*** HEPATITIS A - low sp - TP ***/

data cohort_tp_hep2;
set cohort_tp;
hep=0;
      if    dxcode     in: ('070' '009' '079' '136' '787')  then hep=1;
      else if dx10code_1 in: ('B15' 'B19' 'A09' 'A083' 'A084' 'A085') then
hep=1;
      else if dx10code_2 in: ('B15' 'B19' 'A09' 'A083' 'A084' 'A085') then
hep=1;
      else if dx10code_3 in: ('B15' 'B19' 'A09' 'A083' 'A084' 'A085') then
hep=1;
      run;

data cohort_tp_hep2 (where=(diseasegp=1 and hep=1));
```

```
set cohort_tp_hep2;
run;


/*how many unique individuals?*/

proc sort data=cohort_tp_hep2;
by ikn first_admin_code_date;
run;

data uniqueikn_hep;
set cohort_tp_hep2;
if first.ikn then count=0;
count + 1;
if last.ikn then output;
by ikn;
run; /*48 unique IKNs*/


/*Look at time between iPHIS episode date (onset) and health encounter dates
in admin data*/

data cohort_tp_hep2;
set cohort_tp_hep2;
datediffiphis=date - iphis_accurateepisodedate;
run;

data cohort_tp_hep2;
set cohort_tp_hep2;
if          datediffiphis < -40     then iphisdategp2=1;
else if datediffiphis ge -40 and datediffiphis le 120 then iphisdategp2=2;
else if datediffiphis > 120         then iphisdategp2=3;
run;


/*Apply rule:  IF codes 787 or 079 or 009 or A099 AND iphisdategp2 = 1 or 3,
exclude*/

data cohort_tp_hep3;
set cohort_tp_hep2;
fn=0;
if    dxcode in: ('787' '079' '009') and (iphisdategp2 ne 2)      then fn=1;
else if dx10code_1 in: ('A099') and (iphisdategp2 ne 2)           then fn=1;
run;

proc freq data=cohort_tp_hep3;
tables fn;
run;

data cohort_tp_hep4 (where=(fn ne 1));
set cohort_tp_hep3;
run;


/*remove instances where flag60=1*/

data cohort_tp_hep_flag60 (where=(flag60 ne 1));
```

```
set cohort_tp_hep4;
run;


/*how many unique individuals?*/

proc sort data=cohort_tp_hep_flag60;
by ikn first_admin_code_date;
run;

data uniqueikn_hep;
set cohort_tp_hep_flag60;
if first.ikn then count=0;
count + 1;
if last.ikn then output;
by ikn;
run;


/*high sp*/

data cohort_tp_hep (rename=(dx10code_1=dx10code1_ dx10code_2=dx10code2_
dx10code_3=dx10code3_));
set cohort_tp_hep_flag60;
run;

%MAKEWIDE(data          = cohort_tp_hep,
          out           = source_wide,
          id            = ikn,
          var           = source);

data source_wide;
set source_wide;
run;

%MAKEWIDE(data          = cohort_tp_hep,
          out           = date_wide,
          id            = ikn,
          var           = date);

data date_wide;
set date_wide;
run;

%MAKEWIDE(data          = cohort_tp_hep,
          out           = dxcode_wide,
          id            = ikn,
          var           = dxcode);

data dxcode_wide;
set dxcode_wide;
run;

%MAKEWIDE(data          = cohort_tp_hep,
          out           = dx10code1_wide,
          id            = ikn,
          var           = dx10code1_);
```

```
data dx10code1_wide;
set dx10code1_wide;
run;

%MAKEWIDE(data          = cohort_tp_hep,
          out           = dx10code2_wide,
          id            = ikn,
          var           = dx10code2_);

data dx10code2_wide;
set dx10code2_wide;
run;

%MAKEWIDE(data          = cohort_tp_hep,
          out           = dx10code3_wide,
          id            = ikn,
          var           = dx10code3_);

data dx10code3_wide;
set dx10code3_wide;
run;


/*now merge together*/
data hepa_wide2;
merge source_wide
date_wide
dxcode_wide
dx10code1_wide
dx10code2_wide
dx10code3_wide;
by ikn;
run;


/*Look at constellation*/

/*high*/
data ha1;
set hepa_wide2;
array dxc[15] $ dxcode1-dxcode15;
array dx10c1[15] $dx10code1_1-dx10code1_15;
array dx10c2[15] $dx10code2_1-dx10code2_15;
array dx10c3[15] $dx10code3_1-dx10code3_15;
h_sp=0;
do i=1 to 15;
      if           dxc[i] in:('070')              then h_sp=1;
      else if dx10c1[i] in: ('B15')        then h_sp=1;
      else if dx10c2[i] in: ('B15')        then h_sp=1;
      else if dx10c3[i] in: ('B15')        then h_sp=1;
      end;
      run;

proc freq data=ha1;
tables h_sp;
run;
```

```
/*remove instances where flagsameday=1*/

data cohort_tp_hep_flag0 (where=(flag02 ne 1));
set cohort_tp_hep4;
run;


/*how many unique individuals?*/

proc sort data=cohort_tp_hep_flag0;
by ikn first_admin_code_date;
run;

data uniqueikn_hep;
set cohort_tp_hep_flag0;
if first.ikn then count=0;
count + 1;
if last.ikn then output;
by ikn;
run;


/*high sp*/

data cohort_tp_hep (rename=(dx10code_1=dx10code1_ dx10code_2=dx10code2_
dx10code_3=dx10code3_));
set cohort_tp_hep_flag0;
run;

%MAKEWIDE(data          = cohort_tp_hep,
           out          = source_wide,
           id           = ikn,
           var          = source);

data source_wide;
set source_wide;
run;

%MAKEWIDE(data          = cohort_tp_hep,
           out          = date_wide,
           id           = ikn,
           var          = date);

data date_wide;
set date_wide;
run;

%MAKEWIDE(data          = cohort_tp_hep,
           out          = dxcode_wide,
           id           = ikn,
           var          = dxcode);

data dxcode_wide;
set dxcode_wide;
run;
```

```
%MAKEWIDE(data          = cohort_tp_hep,
           out           = dx10code1_wide,
           id            = ikn,
           var           = dx10code1_);

data dx10code1_wide;
set dx10code1_wide;
run;

%MAKEWIDE(data          = cohort_tp_hep,
           out           = dx10code2_wide,
           id            = ikn,
           var           = dx10code2_);

data dx10code2_wide;
set dx10code2_wide;
run;

%MAKEWIDE(data          = cohort_tp_hep,
           out           = dx10code3_wide,
           id            = ikn,
           var           = dx10code3_);

data dx10code3_wide;
set dx10code3_wide;
run;


/*now merge together*/
data hepa_wide2;
merge source_wide
date_wide
dxcode_wide
dx10code1_wide
dx10code2_wide
dx10code3_wide;
by ikn;
run;


/*Look at constellation*/

/*high*/
data ha1;
set hepa_wide2;
array dxc[15] $ dxcode1-dxcode15;
array dx10c1[15] $dx10code1_1-dx10code1_15;
array dx10c2[15] $dx10code2_1-dx10code2_15;
array dx10c3[15] $dx10code3_1-dx10code3_15;
h_sp=0;
do i=1 to 15;
      if          dxc[i] in:('070')             then h_sp=1;
      else if dx10c1[i] in: ('B15')       then h_sp=1;
      else if dx10c2[i] in: ('B15')       then h_sp=1;
      else if dx10c3[i] in: ('B15')       then h_sp=1;
      end;
```

```
	run;

proc freq data=ha1;
tables h_sp;
run;



/***** MALARIA - low sp - FP *****/

data cohort_fp;
set cohort_fp;
mal=0;
	if          dxcode     in: ('062' '136' '781' '784' '787')  then mal=1;
	else if dx10code_1 in: ('B5' 'P373' 'P374' 'B64')           then mal=1;
	else if dx10code_2 in: ('B5' 'P373' 'P374' 'B64')           then mal=1;
	else if dx10code_3 in: ('B5' 'P373' 'P374' 'B64')           then mal=1;
	run;

data cohort_fp_mal (where=(mal=1));
set cohort_fp;
run;

proc sort data=cohort_fp_mal;
by ikn first_admin_code_date;
run;

data uniqueikn_mal;
set cohort_fp_mal;
if first.ikn then count=0;
count + 1;
if last.ikn then output;
by ikn;
run;


/*remove instances where flag60=1*/

data cohort_fp_mal_flag60 (where=(flag60 ne 1));
set cohort_fp_mal;
run;
/*how many unique individuals?*/

proc sort data=cohort_fp_mal_flag60;
by ikn first_admin_code_date;
run;

data uniqueikn_mal;
set cohort_fp_mal_flag60;
if first.ikn then count=0;
count + 1;
if last.ikn then output;
by ikn;
run;


/*high sp*/
```

```
data cohort_fp_mal_flag60 (rename=(dx10code_1=dx10code1_
dx10code_2=dx10code2_ dx10code_3=dx10code3_));
set cohort_fp_mal_flag60;
run;

%MAKEWIDE(data          = cohort_fp_mal_flag60,
          out           = source_wide,
          id            = ikn,
          var           = source);

data source_wide;
set source_wide;
run;

%MAKEWIDE(data          = cohort_fp_mal_flag60,
          out           = date_wide,
          id            = ikn,
          var           = date);

data date_wide;
set date_wide;
run;

%MAKEWIDE(data          = cohort_fp_mal_flag60,
          out           = dxcode_wide,
          id            = ikn,
          var           = dxcode);

data dxcode_wide;
set dxcode_wide;
run;

%MAKEWIDE(data          = cohort_fp_mal_flag60,
          out           = dx10code1_wide,
          id            = ikn,
          var           = dx10code1_);

data dx10code1_wide;
set dx10code1_wide;
run;

%MAKEWIDE(data          = cohort_fp_mal_flag60,
          out           = dx10code2_wide,
          id            = ikn,
          var           = dx10code2_);

data dx10code2_wide;
set dx10code2_wide;
run;
%MAKEWIDE(data          = cohort_fp_mal_flag60,
          out           = dx10code3_wide,
          id            = ikn,
          var           = dx10code3_);

data dx10code3_wide;
set dx10code3_wide;
```

```
run;


/*now merge together*/
data mal_wide_fp_flag60;
merge source_wide
date_wide
dxcode_wide
dx10code1_wide
dx10code2_wide
dx10code3_wide;
by ikn;
run;


/*1 - highly specificity (h_sp)*/

data m1;
set mal_wide_fp_flag60;
array dxc[298] $ dxcode1-dxcode298;
array dx10c1[298] $dx10code1_1-dx10code1_298;
array dx10c2[298] $dx10code2_1-dx10code2_298;
array dx10c3[298] $dx10code3_1-dx10code3_298;
h_sp=0;
do i=1 to 298;
      if          dxc[i] in:('062') then h_sp=1;
      else if dx10c1[i] in: ('B5')  then h_sp=1;
      else if dx10c2[i] in: ('B5')  then h_sp=1;
      else if dx10c3[i] in: ('B5')  then h_sp=1;
      end;
      run;

proc freq data=m1;
tables h_sp;
run;


/*remove instances where flag02=1*/

data cohort_fp_mal_flag0 (where=(flag02 ne 1));
set cohort_fp_mal;
run;

/*how many unique individuals?*/

proc sort data=cohort_fp_mal_flag0;
by ikn first_admin_code_date;
run;

data uniqueikn_mal;
set cohort_fp_mal_flag0;
if first.ikn then count=0;
count + 1;
if last.ikn then output;
by ikn;
run;
```

```
/*high sp*/

data cohort_fp_mal_flag0 (rename=(dx10code_1=dx10code1_ dx10code_2=dx10code2_
dx10code_3=dx10code3_));
set cohort_fp_mal_flag0;
run;

%MAKEWIDE(data          = cohort_fp_mal_flag0,
          out           = source_wide,
          id            = ikn,
          var           = source);

data source_wide;
set source_wide;
run;

%MAKEWIDE(data          = cohort_fp_mal_flag0,
          out           = date_wide,
          id            = ikn,
          var           = date);

data date_wide;
set date_wide;
run;

%MAKEWIDE(data          = cohort_fp_mal_flag0,
          out           = dxcode_wide,
          id            = ikn,
          var           = dxcode);

data dxcode_wide;
set dxcode_wide;
run;

%MAKEWIDE(data          = cohort_fp_mal_flag0,
          out           = dx10code1_wide,
          id            = ikn,
          var           = dx10code1_);

data dx10code1_wide;
set dx10code1_wide;
run;

%MAKEWIDE(data          = cohort_fp_mal_flag0,
          out           = dx10code2_wide,
          id            = ikn,
          var           = dx10code2_);

data dx10code2_wide;
set dx10code2_wide;
run;

%MAKEWIDE(data          = cohort_fp_mal_flag0,
          out           = dx10code3_wide,
          id            = ikn,
          var           = dx10code3_);
```

```
data dx10code3_wide;
set dx10code3_wide;
run;


/*now merge together*/
data mal_wide_fp_flag0;
merge source_wide
date_wide
dxcode_wide
dx10code1_wide
dx10code2_wide
dx10code3_wide;
by ikn;
run;


/*1 - highly specificity (h_sp)*/

data m1;
set mal_wide_fp_flag0;
array dxc[298] $ dxcode1-dxcode298;
array dx10c1[298] $dx10code1_1-dx10code1_298;
array dx10c2[298] $dx10code2_1-dx10code2_298;
array dx10c3[298] $dx10code3_1-dx10code3_298;
h_sp=0;
do i=1 to 298;
      if          dxc[i] in:('062')             then h_sp=1;
      else if dx10c1[i] in: ('B5')  then h_sp=1;
      else if dx10c2[i] in: ('B5')  then h_sp=1;
      else if dx10c3[i] in: ('B5')  then h_sp=1;
      end;
      run;

proc freq data=m1;
tables h_sp;
run;



/***** MALARIA - TP *****/

data cohort_tp_mal2;
set cohort_tp;
mal=0;
      if          dxcode     in: ('062' '136' '781' '784' '787')  then mal=1;
      else if dx10code_1 in: ('B5' 'P373' 'P374' 'B64')           then mal=1;
      else if dx10code_2 in: ('B5' 'P373' 'P374' 'B64')           then mal=1;
      else if dx10code_3 in: ('B5' 'P373' 'P374' 'B64')           then mal=1;
      run;

data cohort_tp_mal2 (where=(diseasegp=2 and mal=1));
set cohort_tp_mal2;
run;
```

```
/*Look at time between iPHIS episode date (onset) and health encounter dates
in admin data*/

data cohort_tp_mal2;
set cohort_tp_mal2;
datediffiphis=date - iphis_accurateepisodedate;
run;

data cohort_tp_mal2;
set cohort_tp_mal2;
if           datediffiphis < -30     then iphisdategp2=1;
else if datediffiphis ge -30 and datediffiphis le 120 then iphisdategp2=2;
else if datediffiphis > 120         then iphisdategp2=3;
run;

/*Apply rule:  IF codes 781, 787 or 136 AND iphisdategp2 = 1 or 3, exclude*/

data cohort_tp_mal3;
set cohort_tp_mal2;
fn=0;
if dxcode in: ('136' '781' '787') and (iphisdategp2 ne 2)   then fn=1;
run;

data cohort_tp_mal4 (where=(fn ne 1));
set cohort_tp_mal3;
run;

/*remove instances where flag60=1*/

data cohort_tp_mal_flag60 (where=(flag60 ne 1));
set cohort_tp_mal4;
run;

/*how many unique individuals?*/

proc sort data=cohort_tp_mal_flag60;
by ikn first_admin_code_date;
run;

data uniqueikn_mal;
set cohort_tp_mal_flag60;
if first.ikn then count=0;
count + 1;
if last.ikn then output;
by ikn;
run;


/*h_sp*/

data cohort_tp_mal_flag60 (rename=(dx10code_1=dx10code1_
dx10code_2=dx10code2_ dx10code_3=dx10code3_));
set cohort_tp_mal_flag60;
run;

%MAKEWIDE(data          = cohort_tp_mal_flag60,
           out          = source_wide,
```

```
            id          = ikn,
            var         = source);

data source_wide;
set source_wide;
run;

%MAKEWIDE(data          = cohort_tp_mal_flag60,
            out         = date_wide,
            id          = ikn,
            var         = date);

data date_wide;
set date_wide;
run;

%MAKEWIDE(data          = cohort_tp_mal_flag60,
            out         = dxcode_wide,
            id          = ikn,
            var         = dxcode);

data dxcode_wide;
set dxcode_wide;
run;

%MAKEWIDE(data          = cohort_tp_mal_flag60,
            out         = dx10code1_wide,
            id          = ikn,
            var         = dx10code1_);

data dx10code1_wide;
set dx10code1_wide;
run;

%MAKEWIDE(data          = cohort_tp_mal_flag60,
            out         = dx10code2_wide,
            id          = ikn,
            var         = dx10code2_);

data dx10code2_wide;
set dx10code2_wide;
run;

%MAKEWIDE(data          = cohort_tp_mal_flag60,
            out         = dx10code3_wide,
            id          = ikn,
            var         = dx10code3_);

data dx10code3_wide;
set dx10code3_wide;
run;


/*now merge together*/
data mal_wide_tp_flag60;
merge source_wide
date_wide
```

```
dxcode_wide
dx10code1_wide
dx10code2_wide
dx10code3_wide;
by ikn;
run;

data m1;
set mal_wide_tp_flag60;
array dxc[37] $ dxcode1-dxcode37;
array dx10c1[37] $dx10code1_1-dx10code1_37;
array dx10c2[37] $dx10code2_1-dx10code2_37;
array dx10c3[37] $dx10code3_1-dx10code3_37;
h_sp=0;
do i=1 to 37;
      if    dxc[i] in: ('062')      then h_sp=1;
      else if dx10c1[i] in: ('B5')  then h_sp=1;
      else if dx10c2[i] in: ('B5')  then h_sp=1;
      else if dx10c3[i] in: ('B5')  then h_sp=1;
      end;
      run;

proc freq data=m1;
tables h_sp;
run;



/*remove instances where flag02=1*/

data cohort_tp_mal_flag0 (where=(flag02 ne 1));
set cohort_tp_mal4;
run;

/*how many unique individuals?*/

proc sort data=cohort_tp_mal_flag0;
by ikn first_admin_code_date;
run;

data uniqueikn_mal;
set cohort_tp_mal_flag0;
if first.ikn then count=0;
count + 1;
if last.ikn then output;
by ikn;
run;


/*high sp*/
data cohort_tp_mal_flag0 (rename=(dx10code_1=dx10code1_ dx10code_2=dx10code2_
dx10code_3=dx10code3_));
set cohort_tp_mal_flag0;
run;

%MAKEWIDE(data          = cohort_tp_mal_flag0,
          out           = source_wide,
```

```
           id           = ikn,
           var          = source);

data source_wide;
set source_wide;
run;

%MAKEWIDE(data          = cohort_tp_mal_flag0,
           out          = date_wide,
           id           = ikn,
           var          = date);

data date_wide;
set date_wide;
run;

%MAKEWIDE(data          = cohort_tp_mal_flag0,
           out          = dxcode_wide,
           id           = ikn,
           var          = dxcode);

data dxcode_wide;
set dxcode_wide;
run;

%MAKEWIDE(data          = cohort_tp_mal_flag0,
           out          = dx10code1_wide,
           id           = ikn,
           var          = dx10code1_);

data dx10code1_wide;
set dx10code1_wide;
run;

%MAKEWIDE(data          = cohort_tp_mal_flag0,
           out          = dx10code2_wide,
           id           = ikn,
           var          = dx10code2_);

data dx10code2_wide;
set dx10code2_wide;
run;

%MAKEWIDE(data          = cohort_tp_mal_flag0,
           out          = dx10code3_wide,
           id           = ikn,
           var          = dx10code3_);

data dx10code3_wide;
set dx10code3_wide;
run;


/*now merge together*/
data mal_wide_tp_flag0;
merge source_wide
date_wide
```

```
dxcode_wide
dx10code1_wide
dx10code2_wide
dx10code3_wide;
by ikn;
run;

data m1;
set mal_wide_tp_flag0;
array dxc[37] $ dxcode1-dxcode37;
array dx10c1[37] $dx10code1_1-dx10code1_37;
array dx10c2[37] $dx10code2_1-dx10code2_37;
array dx10c3[37] $dx10code3_1-dx10code3_37;
h_sp=0;
do i=1 to 37;
      if    dxc[i] in: ('062')      then h_sp=1;
      else if dx10c1[i] in: ('B5')  then h_sp=1;
      else if dx10c2[i] in: ('B5')  then h_sp=1;
      else if dx10c3[i] in: ('B5')  then h_sp=1;
      end;
      run;

proc freq data=m1;
tables h_sp;
run;




/***** T/PT - low sp - FP *****/

data cohort_fp;
set cohort_fp;
tpt=0;
      if dxcode  in: ('002' '009' '003' '005' '136' '784' '787') then tpt=1;
      else if dx10code_1 in: ('A01' 'A029' 'A021' 'A049' 'A059' 'A09' 'A499')
then tpt=1;
      else if dx10code_2 in: ('A01' 'A029' 'A021' 'A049' 'A059' 'A09' 'A499')
then tpt=1;
      else if dx10code_3 in: ('A01' 'A029' 'A021' 'A049' 'A059' 'A09' 'A499')
then tpt=1;
      run;

data cohort_fp_tpt (where=(tpt=1));
set cohort_fp;
run;


/*remove instances where flag60=1*/

data cohort_fp_tpt_flag60 (where=(flag60 ne 1));
set cohort_fp_tpt;
run;

/*how many unique individuals?*/

proc sort data=cohort_fp_tpt_flag60;
```

```
by ikn first_admin_code_date;
run;

data uniqueikn_tpt;
set cohort_fp_tpt_flag60;
if first.ikn then count=0;
count + 1;
if last.ikn then output;
by ikn;
run;


/*high sp*/

data cohort_fp_tpt_flag60 (rename=(dx10code_1=dx10code1_
dx10code_2=dx10code2_ dx10code_3=dx10code3_));
set cohort_fp_tpt_flag60;
run;

%MAKEWIDE(data          = cohort_fp_tpt_flag60,
          out           = source_wide,
          id            = ikn,
          var           = source);

data source_wide;
set source_wide;
run;

%MAKEWIDE(data          = cohort_fp_tpt_flag60,
          out           = date_wide,
          id            = ikn,
          var           = date);

data date_wide;
set date_wide;
run;

%MAKEWIDE(data          = cohort_fp_tpt_flag60,
          out           = dxcode_wide,
          id            = ikn,
          var           = dxcode);

data dxcode_wide;
set dxcode_wide;
run;

%MAKEWIDE(data          = cohort_fp_tpt_flag60,
          out           = dx10code1_wide,
          id            = ikn,
          var           = dx10code1_);

data dx10code1_wide;
set dx10code1_wide;
run;

%MAKEWIDE(data          = cohort_fp_tpt_flag60,
          out           = dx10code2_wide,
```

```
            id          = ikn,
            var         = dx10code2_);

data dx10code2_wide;
set dx10code2_wide;
run;

%MAKEWIDE(data          = cohort_fp_tpt_flag60,
            out         = dx10code3_wide,
            id          = ikn,
            var         = dx10code3_);

data dx10code3_wide;
set dx10code3_wide;
run;


/*now merge together*/
data tpt_wide_fp_flag60;
merge source_wide
date_wide
dxcode_wide
dx10code1_wide
dx10code2_wide
dx10code3_wide;
by ikn;
run;


data t1;
set tpt_wide_fp_flag60;
array dxc[285] $ dxcode1-dxcode285;
array dx10c1[285] $dx10code1_1-dx10code1_285;
array dx10c2[285] $dx10code2_1-dx10code2_285;
array dx10c3[285] $dx10code3_1-dx10code3_285;
h_sp=0;
do i=1 to 285;
      if          dxc[i] in: ('002')            then h_sp=1;
      else if dx10c1[i] in: ('A01')       then h_sp=1;
      else if dx10c2[i] in: ('A01')       then h_sp=1;
      else if dx10c3[i] in: ('A01')       then h_sp=1;
      end;
      run;

proc freq data=t1;
tables h_sp;
run;


/*remove instances where flag02=1*/

data cohort_fp_tpt_flag0 (where=(flag02 ne 1));
set cohort_fp_tpt;
run;

/*how many unique individuals?*/
```

```
proc sort data=cohort_fp_tpt_flag0;
by ikn first_admin_code_date;
run;

data uniqueikn_tpt;
set cohort_fp_tpt_flag0;
if first.ikn then count=0;
count + 1;
if last.ikn then output;
by ikn;
run;


/*high sp*/

data cohort_fp_tpt_flag0 (rename=(dx10code_1=dx10code1_ dx10code_2=dx10code2_
dx10code_3=dx10code3_));
set cohort_fp_tpt_flag0;
run;

%MAKEWIDE(data          = cohort_fp_tpt_flag0,
          out           = source_wide,
          id            = ikn,
          var           = source);

data source_wide;
set source_wide;
run;

%MAKEWIDE(data          = cohort_fp_tpt_flag0,
          out           = date_wide,
          id            = ikn,
          var           = date);

data date_wide;
set date_wide;
run;

%MAKEWIDE(data          = cohort_fp_tpt_flag0,
          out           = dxcode_wide,
          id            = ikn,
          var           = dxcode);

data dxcode_wide;
set dxcode_wide;
run;

%MAKEWIDE(data          = cohort_fp_tpt_flag0,
          out           = dx10code1_wide,
          id            = ikn,
          var           = dx10code1_);

data dx10code1_wide;
set dx10code1_wide;
run;

%MAKEWIDE(data          = cohort_fp_tpt_flag0,
```

```
            out          = dx10code2_wide,
            id           = ikn,
            var          = dx10code2_);

data dx10code2_wide;
set dx10code2_wide;
run;

%MAKEWIDE(data         = cohort_fp_tpt_flag0,
            out          = dx10code3_wide,
            id           = ikn,
            var          = dx10code3_);

data dx10code3_wide;
set dx10code3_wide;
run;


/*now merge together*/
data tpt_wide_fp_flag0;
merge source_wide
date_wide
dxcode_wide
dx10code1_wide
dx10code2_wide
dx10code3_wide;
by ikn;
run;


data t1;
set tpt_wide_fp_flag0;
array dxc[285] $ dxcode1-dxcode285;
array dx10c1[285] $dx10code1_1-dx10code1_285;
array dx10c2[285] $dx10code2_1-dx10code2_285;
array dx10c3[285] $dx10code3_1-dx10code3_285;
h_sp=0;
do i=1 to 285;
      if          dxc[i] in: ('002')      then h_sp=1;
      else if dx10c1[i] in: ('A01')       then h_sp=1;
      else if dx10c2[i] in: ('A01')       then h_sp=1;
      else if dx10c3[i] in: ('A01')       then h_sp=1;
      end;
      run;

proc freq data=t1;
tables h_sp;
run;




/***** T/PT - low sp - TP *****/

data cohort_tp_tpt2;
set cohort_tp;
tpt=0;
```

```
      if dxcode in: ('002' '009' '003' '005' '136' '784' '787')   then tpt=1;
      else if dx10code_1 in: ('A01' 'A029' 'A021' 'A049' 'A059' 'A09' 'A499')
then tpt=1;
      else if dx10code_2 in: ('A01' 'A029' 'A021' 'A049' 'A059' 'A09' 'A499')
then tpt=1;
      else if dx10code_3 in: ('A01' 'A029' 'A021' 'A049' 'A059' 'A09' 'A499')
then tpt=1;
      run;

data cohort_tp_tpt2 (where=(diseasegp=3 and tpt=1));
set cohort_tp_tpt2;
run;


/*need to correct for two cases with two distinct disease episodes*/

data duplicates;
set cohort_tp_tpt2;
dup=0;
if ikn=X or ikn=X then dup=1;
run;

data duplicates (where=(dup=1));
set duplicates;
run;

data duplicates (drop=iphis_accurateepisodedate iphis_disease
iphis_countryofbirth iphis_travelassociated iphis_traveldestination1);
set duplicates;
run;

data duplicates;
set duplicates;
length rec $20;
rec='rec2';
run;

data duplicates;
set duplicates;
length ikn2 $20;
ikn2=catx("_", of ikn rec);
run;

data duplicates (drop=ikn dup _a rec);
set duplicates;
run;

data duplicates;
rename ikn2=ikn iphis_accurateepisodedate_rec2=iphis_accurateepisodedate
iphis_disease_rec2=iphis_disease
iphis_countryofbirth_rec2=iphis_countryofbirth
iphis_travelassociated_rec2=iphis_travelassociated
iphis_traveldestination1_rec2=iphis_traveldestination1;
set duplicates;
run;

data duplicates;
```

```
set duplicates;
run;

/*now append to cohort of TP*/

data cohort_tp_tpt3 (drop=iphis_accurateepisodedate_rec2 iphis_disease_rec2
iphis_countryofbirth_rec2 iphis_travelassociated_rec2
iphis_traveldestination1_rec2);
set cohort_tp_tpt2;
run;

data cohort_tp_tpt3;
set cohort_tp_tpt3;
length ikn2 $20;
ikn2=ikn;
run;

data cohort_tp_tpt3 (drop=ikn);
set cohort_tp_tpt3;
run;

data cohort_tp_tpt3 (rename=ikn2=ikn);
set cohort_tp_tpt3;
run;

proc append base=cohort_tp_tpt3 data=duplicates;
run;

data cohort_tp_tpt_append;
set cohort_tp_tpt3;
run;

data cohort_tp_tpt_append;
retain ikn iphis iphis_accurateepisodedate iphis_disease iphis_countryofbirth
iphis_travelassociated iphis_traveldestination1
first_admin_code_date cohort_entry age_cohort_entry sex cic_immigrant
cic_fsrce   admin_code_in_lookback elig_at_iphis elig_1yr
elig_2yr elig_3yr source date ddate dxcode dx10code_1 dxtype_1 dxpref_1
      dx10code_2 dxtype_2     dxpref_2 dx10code_3     dxtype_3 dxpref_3
peel_region age   diseasegp tpt;
set cohort_tp_tpt3;
run;

data cohort_tp_tpt_append;
set cohort_tp_tpt_append;
run;


/*how many unique individuals?*/

proc sort data=cohort_tp_tpt_append;
by ikn first_admin_code_date;
run;

data uniqueikn_tpt;
set cohort_tp_tpt_append;
if first.ikn then count=0;
```

```
count + 1;
if last.ikn then output;
by ikn;
run;


/*Look at time between iPHIS episode date (onset) and health encounter dates
in admin data*/

data cohort_tp_tpt2;
set cohort_tp_tpt_append;
datediffiphis=date - iphis_accurateepisodedate;
run;

data cohort_tp_tpt2;
set cohort_tp_tpt2;
if          datediffiphis < -30           then iphisdategp3=1;
else if datediffiphis ge -30 and datediffiphis le 200 then iphisdategp3=2;
else if datediffiphis > 200               then iphisdategp3=3;
run;

/*Apply rule:  IF codes 787 or 009 or 136 or A099 AND iphisdategp3 = 1 or 3,
exclude*/

data cohort_tp_tpt3;
set cohort_tp_tpt2;
fn=0;
if    dxcode in: ('009' '787' '136') and (iphisdategp3 ne 2)      then fn=1;
else if dx10code_1 in: ('A099') and (iphisdategp3 ne 2)           then fn=1;
run;


proc freq data=cohort_tp_tpt3;
tables fn;
run;

proc sort data=cohort_tp_tpt3;
by fn ikn;
run;

data cohort_tp_tpt4 (where=(fn ne 1));
set cohort_tp_tpt3;
run;


/*remove instances where flag60=1*/

data cohort_tp_tpt_flag60 (where=(flag60 ne 1));
set cohort_tp_tpt4;
run;

/*how many unique individuals?*/

proc sort data=cohort_tp_tpt_flag60;
by ikn first_admin_code_date;
run;
```

```
data uniqueikn_tpt;
set cohort_tp_tpt_flag60;
if first.ikn then count=0;
count + 1;
if last.ikn then output;
by ikn;
run;

data cohort_tp_tpt_flag60 (rename=(dx10code_1=dx10code1_
dx10code_2=dx10code2_ dx10code_3=dx10code3_));
set cohort_tp_tpt_flag60;
run;

%MAKEWIDE(data          = cohort_tp_tpt_flag60,
          out           = source_wide,
          id            = ikn,
          var           = source);

data source_wide;
set source_wide;
run;

%MAKEWIDE(data          = cohort_tp_tpt_flag60,
          out           = date_wide,
          id            = ikn,
          var           = date);

data date_wide;
set date_wide;
run;

%MAKEWIDE(data          = cohort_tp_tpt_flag60,
          out           = dxcode_wide,
          id            = ikn,
          var           = dxcode);

data dxcode_wide;
set dxcode_wide;
run;

%MAKEWIDE(data          = cohort_tp_tpt_flag60,
          out           = dx10code1_wide,
          id            = ikn,
          var           = dx10code1_);

data dx10code1_wide;
set dx10code1_wide;
run;

%MAKEWIDE(data          = cohort_tp_tpt_flag60,
          out           = dx10code2_wide,
          id            = ikn,
          var           = dx10code2_);

data dx10code2_wide;
set dx10code2_wide;
run;
```

```
%MAKEWIDE(data          = cohort_tp_tpt_flag60,
           out          = dx10code3_wide,
           id           = ikn,
           var          = dx10code3_);
data dx10code3_wide;
set dx10code3_wide;
run;

/*now merge together*/
data tpt_tp_wide_flag60;
merge source_wide
date_wide
dxcode_wide
dx10code1_wide
dx10code2_wide
dx10code3_wide;
by ikn;
run;


/*Look at constellation*/

data t1;
set tpt_tp_wide_flag60;
array dxc[25] $ dxcode1-dxcode25;
array dx10c1[25] $dx10code1_1-dx10code1_25;
array dx10c2[25] $dx10code2_1-dx10code2_25;
array dx10c3[25] $dx10code3_1-dx10code3_25;
h_sp=0;
do i=1 to 25;
      if          dxc[i] in: ('002')            then h_sp=1;
      else if dx10c1[i] in: ('A01')       then h_sp=1;
      else if dx10c2[i] in: ('A01')       then h_sp=1;
      else if dx10c3[i] in: ('A01')       then h_sp=1;
      end;
      run;

proc freq data=t1;
tables h_sp;
run;


/*remove instances where flag02=1*/

data cohort_tp_tpt_flag0 (where=(flag02 ne 1));
set cohort_tp_tpt4;
run;

/*how many unique individuals?*/

proc sort data=cohort_tp_tpt_flag0;
by ikn first_admin_code_date;
run;

data uniqueikn_tpt;
set cohort_tp_tpt_flag0;
```

```
if first.ikn then count=0;
count + 1;
if last.ikn then output;
by ikn;
run;


/*high sp*/

data cohort_tp_tpt_flag0 (rename=(dx10code_1=dx10code1_ dx10code_2=dx10code2_
dx10code_3=dx10code3_));
set cohort_tp_tpt_flag0;
run;

%MAKEWIDE(data          = cohort_tp_tpt_flag0,
           out          = source_wide,
           id           = ikn,
           var          = source);

data source_wide;
set source_wide;
run;

%MAKEWIDE(data          = cohort_tp_tpt_flag0,
           out          = date_wide,
           id           = ikn,
           var          = date);

data date_wide;
set date_wide;
run;

%MAKEWIDE(data          = cohort_tp_tpt_flag0,
           out          = dxcode_wide,
           id           = ikn,
           var          = dxcode);

data dxcode_wide;
set dxcode_wide;
run;

%MAKEWIDE(data          = cohort_tp_tpt_flag0,
           out          = dx10code1_wide,
           id           = ikn,
           var          = dx10code1_);

data dx10code1_wide;
set dx10code1_wide;
run;

%MAKEWIDE(data          = cohort_tp_tpt_flag0,
           out          = dx10code2_wide,
           id           = ikn,
           var          = dx10code2_);

data dx10code2_wide;
set dx10code2_wide;
```

```
run;

%MAKEWIDE(data          = cohort_tp_tpt_flag0,
          out           = dx10code3_wide,
          id            = ikn,
          var           = dx10code3_);
data dx10code3_wide;
set dx10code3_wide;
run;

/*now merge together*/
data tpt_tp_wide_flag0;
merge source_wide
date_wide
dxcode_wide
dx10code1_wide
dx10code2_wide
dx10code3_wide;
by ikn;
run;


/*Look at constellation*/

data t1;
set tpt_tp_wide_flag0;
array dxc[25] $ dxcode1-dxcode25;
array dx10c1[25] $dx10code1_1-dx10code1_25;
array dx10c2[25] $dx10code2_1-dx10code2_25;
array dx10c3[25] $dx10code3_1-dx10code3_25;
h_sp=0;
do i=1 to 25;
      if           dxc[i] in: ('002')       then h_sp=1;
      else if dx10c1[i] in: ('A01')         then h_sp=1;
      else if dx10c2[i] in: ('A01')         then h_sp=1;
      else if dx10c3[i] in: ('A01')         then h_sp=1;
      end;
      run;

proc freq data=t1;
tables h_sp;
run;


/**no material impact on sens or PPV - see G538 Excel file for results**/

data immunize;
set immunize;
run;

proc sort data=immunize;
by servdate;
run;
```

```
/*3. Sensitivity Analysis - Adjust definition for admin in lookback (from any
to same) to see if more discriminatory perhaps to be used to further exclude
FP patients*/


data admin;
set MYDATA.admin_codes_lookback_02may2017;
run;

proc sort data=admin;
by date;
run;

proc freq data=admin;
tables dxcode dx10code:;
run;

data admin (drop=ddate dx10code_3 dxtype_3 dxpref_3);
set admin;
run;

data admin;
set admin;
datediff=date-first_admin_code_date;
run;

proc means min max data=admin;
var datediff;
run;

/*flag if within first three months b/c may wish to exclude (-89 and -1
day)*/
data admin;
set admin;
firstthree=0;
if datediff ge -89 and datediff le -1 then firstthree=1;
run;

proc freq data=admin;
tables firstthree;
run; /*6.2%*/

data admin (rename=(source=sourcea date=datea dxcode=dxcodea
dx10code_1=dx10code_1a dx10code_2=dx10code_2a datediff=admindatediff));
set admin;
run;

data admin (keep=ikn sourcea datea dxcodea dx10code_1a dx10code_2a
admindatediff firstthree);
set admin;
run;


/*now merge with original cohort to create a flag if code is the same*/

/*need to do a Cartesian product between matching vars*/
```

```
proc sql;
create table botha as
      select cohort.ikn
      ,cohort.first_admin_code_date
      ,cohort.source
      ,cohort.dxcode
      ,cohort.dx10code_1
      ,cohort.dx10code_2
      ,cohort.date
      ,cohort.admin_code_in_lookback
      ,admin.sourcea
      ,admin.datea
      ,admin.dxcodea
      ,admin.dx10code_1a
      ,admin.dx10code_2a
      ,admin.admindatediff
      ,admin.firstthree
from cohort
      ,admin
where cohort.ikn=admin.ikn;
quit;

data botha;
set botha;
run;

proc sort data=botha;
by admin_code_in_lookback;
run;

/*check*/
proc freq datadata=botha;
tables admin_code_in_lookback admin_code_in_lookback*firstthree;
run;

/*create var if diagnostic code is the same code as in lookback*/
data botha;
set botha;
adminsame=0;
if    dxcode=dxcodea                                 then adminsame=1;
else if (dx10code_1=dx10code_1a or dx10code_1=dx10code_2a) and dx10code_1 ne
' '   then adminsame=1;
else if (dx10code_2=dx10code_1a or dx10code_2=dx10code_2a) and dx10code_2 ne
' '   then adminsame=1;
run;


/*create three more vars which are if same but specify code*/
data botha;
set botha;
adminsame070=0;
if dxcodea in ('070') and dxcode=dxcodea  then adminsame070=1;
run;

proc sort data=botha;
by adminsame070;
run;
```

```
data botha;
set botha;
adminsame002=0;
if dxcodea in ('002') and dxcode=dxcodea  then adminsame002=1;
run;

proc sort data=botha;
by adminsame002;
run;

data botha;
set botha;
adminsame136=0;
if dxcodea in ('136') and dxcode=dxcodea  then adminsame136=1;
run;

proc sort data=botha;
by adminsame136;
run;


data botha;
set botha;
run;


/*now create a data set of just these vars to merge with original*/
data bothb (keep=ikn firstthree adminsame);
set botha;
run;

data bothb (where=(adminsame=1));
set bothb;
run;

data bothb (where=(firstthree=0));
set bothb;
run;

proc sort data=bothb
out=bothb nodupkey;
by ikn firstthree adminsame;
run;

data bothb (drop=firstthree);
set bothb;
run;

/*Check for duplicate IKNs*/

proc freq data=bothb;
tables ikn/noprint out=keylist;
run;
proc print;
where count ge 2;
run;
```

```
/*070 flag dataset*/
data both070 (keep=ikn firstthree adminsame070 dxcodea);
set botha;
run;

data both070 (where=(adminsame070=1));
set both070;
run;

data both070 (where=(firstthree=0));
set both070;
run;

proc sort data=both070
out=both070 nodupkey;
by ikn firstthree adminsame070;
run;

data both070 (drop=firstthree);
set both070;
run;

/*Check for duplicate IKNs*/

proc freq data=both070;
tables ikn/noprint out=keylist;
run;
proc print;
where count ge 2;
run;

data both070;
set both070;
run;


/*now link both070 to TP and FP datasets for hepA*/

data hepa_fp_admin070;
set DATASETS.final_hepa_fp_02may2017;
run;

data hepa_fp_admin070 (where=(h_sp=1));
set hepa_fp_admin070;
run;

data hepa_fp_admin070;
merge hepa_fp_admin070 (in=fp) both070 (in=flag);
by ikn;
if fp; /*keeps all in cohort*/
run;

proc freq data=hepa_fp_admin070;
```

```
tables adminsame070 admin_code_in_lookback
adminsame070*admin_code_in_lookback c_070 dxcodea;
run; /*3567/15951 FP have 070 in lookback*/


data hepa_tp_admin070;
set DATASETS.final_hepa_tp_02may2017;
run;

data hepa_tp_admin070 (where=(h_sp=1));
set hepa_tp_admin070;
run;

data hepa_tp_admin070;
merge hepa_tp_admin070 (in=tp) both070 (in=flag);
by ikn;
if tp; /*keeps all in cohort*/
run;

proc freq data=hepa_tp_admin070;
tables adminsame070 admin_code_in_lookback
adminsame070*admin_code_in_lookback code070 dxcodea;
run; /*0/41 have 070 in lookback*/

/***/


/*a) Hepatitis A*/

data cohort_fp (where=(iphis=0 and first_admin_code_date ne .));
set cohort;
run;

data cohort_fp;
set cohort_fp;
hep=0;
      if    dxcode    in: ('070' '009' '079' '136' '787')   then hep=1;
      else if dx10code_1 in: ('B15' 'B19' 'A09' 'A083' 'A084' 'A085') then
hep=1;
      else if dx10code_2 in: ('B15' 'B19' 'A09' 'A083' 'A084' 'A085') then
hep=1;
      else if dx10code_3 in: ('B15' 'B19' 'A09' 'A083' 'A084' 'A085') then
hep=1;
      run;

proc freq data=cohort_fp;
tables hep;
run;

data cohort_fp_hep (where=(hep=1));
set cohort_fp;
run;

/*Look at time between first and last date in individuals with >1 episode*/

data cohort_fp_hep;
set cohort_fp_hep;
```

```
datediffo=date-first_admin_code_date;
run;

proc sort data=cohort_fp_hep;
by ikn first_admin_code_date date;
run;

data episodeperiod_hep;
set cohort_fp_hep;
if first.ikn then count=0;
count + 1;
if last.ikn then output;
by ikn;
run;

proc means range data=episodeperiod_hep;
var count;
run;


/*now, flatten so one row per IKN:*/

data cohort_fp_hep (rename=(dx10code_1=dx10code1_ dx10code_2=dx10code2_
dx10code_3=dx10code3_));
set cohort_fp_hep;
run;

%MAKEWIDE(data          = cohort_fp_hep,
          out           = source_wide,
          id            = ikn,
          var           = source);

data source_wide;
set source_wide;
run;

%MAKEWIDE(data          = cohort_fp_hep,
          out           = date_wide,
          id            = ikn,
          var           = date);

data date_wide;
set date_wide;
run;

%MAKEWIDE(data          = cohort_fp_hep,
          out           = dxcode_wide,
          id            = ikn,
          var           = dxcode);

data dxcode_wide;
set dxcode_wide;
run;

%MAKEWIDE(data          = cohort_fp_hep,
          out           = dx10code1_wide,
          id            = ikn,
```

```
            var          = dx10code1_);

data dx10code1_wide;
set dx10code1_wide;
run;

%MAKEWIDE(data           = cohort_fp_hep,
            out          = dx10code2_wide,
            id           = ikn,
            var          = dx10code2_);

data dx10code2_wide;
set dx10code2_wide;
run;

%MAKEWIDE(data           = cohort_fp_hep,
            out          = dx10code3_wide,
            id           = ikn,
            var          = dx10code3_);

data dx10code3_wide;
set dx10code3_wide;
run;


/*now merge together*/
data hepa_wide_fp;
merge source_wide
date_wide
dxcode_wide
dx10code1_wide
dx10code2_wide
dx10code3_wide;
by ikn;
run;


/*1 - highly specificity (h_sp)*/

data ha1;
set hepa_wide_fp;
array dxc[209] $ dxcode1-dxcode209;
array dx10c1[209] $dx10code1_1-dx10code1_209;
array dx10c2[209] $dx10code2_1-dx10code2_209;
array dx10c3[209] $dx10code3_1-dx10code3_209;
h_sp=0;
do i=1 to 209;
      if          dxc[i] in:('070')       then h_sp=1;
      else if dx10c1[i] in: ('B15')       then h_sp=1;
      else if dx10c2[i] in: ('B15')       then h_sp=1;
      else if dx10c3[i] in: ('B15')       then h_sp=1;
      end;
      run;

proc freq data=ha1;
tables h_sp;
run;
```

```
/*2 - moderate specificity (m_sp)*/

data ha1;
set ha1;
array dxc[209] $ dxcode1-dxcode209;
array dx10c1[209] $dx10code1_1-dx10code1_209;
array dx10c2[209] $dx10code2_1-dx10code2_209;
array dx10c3[209] $dx10code3_1-dx10code3_209;
m_sp=0;
do i=1 to 209;
      if           dxc[i] in:('009')             then m_sp=1;
      else if dx10c1[i] in: ('B19')       then m_sp=1;
      else if dx10c2[i] in: ('B19')       then m_sp=1;
      else if dx10c3[i] in: ('B19')       then m_sp=1;
      end;
      run;

proc freq data=ha1;
tables m_sp;
run;


/*3 - low specificity (l_sp)*/

data ha1;
set ha1;
array dxc[209] $ dxcode1-dxcode209;
array dx10c1[209] $dx10code1_1-dx10code1_209;
array dx10c2[209] $dx10code2_1-dx10code2_209;
array dx10c3[209] $dx10code3_1-dx10code3_209;
l_sp=0;
do i=1 to 209;
      if           dxc[i] in: ('079' '136' '787')      then l_sp=1;
      else if dx10c1[i] in: ('A09' 'A083' 'A084' 'A085')    then l_sp=1;
      else if dx10c2[i] in: ('A09' 'A083' 'A084' 'A085')    then l_sp=1;
      else if dx10c3[i] in: ('A09' 'A083' 'A084' 'A085')    then l_sp=1;
      end;
      run;

proc freq data=ha1;
tables l_sp;
run;


data ha1;
set ha1;
array dxc[209] $ dxcode1-dxcode209;
c_070=0;
do i=1 to 209;
      if    dxc[i] in: ('070') then c_070=1;
      end;
      run;

data ha1;
set ha1;
```

```
array dx10c1[209] $dx10code1_1-dx10code1_209;
array dx10c2[209] $dx10code2_1-dx10code2_209;
array dx10c3[209] $dx10code3_1-dx10code3_209;
c_B15=0;
do i=1 to 209;
      if dx10c1[i] in: ('B15')            then c_B15=1;
      else if dx10c2[i] in: ('B15')       then c_B15=1;
      else if dx10c3[i] in: ('B15')       then c_B15=1;
      end;
      run;

data ha1;
set ha1;
array dx10c1[209] $dx10code1_1-dx10code1_209;
array dx10c2[209] $dx10code2_1-dx10code2_209;
array dx10c3[209] $dx10code3_1-dx10code3_209;
c_A084=0;
do i=1 to 209;
      if dx10c1[i] in: ('A084')           then c_A084=1;
      else if dx10c2[i] in: ('A084')      then c_A084=1;
      else if dx10c3[i] in: ('A084')      then c_A084=1;
      end;
      run;

data ha1;
set ha1;
array dx10c1[209] $dx10code1_1-dx10code1_209;
array dx10c2[209] $dx10code2_1-dx10code2_209;
array dx10c3[209] $dx10code3_1-dx10code3_209;
c_A09=0;
do i=1 to 209;
      if dx10c1[i] in: ('A09')            then c_A09=1;
      else if dx10c2[i] in: ('A09')       then c_A09=1;
      else if dx10c3[i] in: ('A09')       then c_A09=1;
      end;
      run;

data ha1;
set ha1;
array dx10c1[209] $dx10code1_1-dx10code1_209;
array dx10c2[209] $dx10code2_1-dx10code2_209;
array dx10c3[209] $dx10code3_1-dx10code3_209;
c_B19=0;
do i=1 to 209;
      if dx10c1[i] in: ('B19')            then c_B19=1;
      else if dx10c2[i] in: ('B19')       then c_B19=1;
      else if dx10c3[i] in: ('B19')       then c_B19=1;
      end;
      run;

data ha1;
set ha1;
array dxc[209] $ dxcode1-dxcode209;
array dx10c1[209] $dx10code1_1-dx10code1_209;
array dx10c2[209] $dx10code2_1-dx10code2_209;
array dx10c3[209] $dx10code3_1-dx10code3_209;
code009=0;
```

```
do i=1 to 209;
      if          dxc[i] in:('009')              then code009=1;
      end;
      run;

data ha1;
set ha1;
array dxc[209] $ dxcode1-dxcode209;
array dx10c1[209] $dx10code1_1-dx10code1_209;
array dx10c2[209] $dx10code2_1-dx10code2_209;
array dx10c3[209] $dx10code3_1-dx10code3_209;
code079=0;
do i=1 to 209;
      if          dxc[i] in:('079')              then code079=1;
      end;
      run;

data ha1;
set ha1;
array dxc[209] $ dxcode1-dxcode209;
array dx10c1[209] $dx10code1_1-dx10code1_209;
array dx10c2[209] $dx10code2_1-dx10code2_209;
array dx10c3[209] $dx10code3_1-dx10code3_209;
code787=0;
do i=1 to 209;
      if          dxc[i] in:('787')              then code787=1;
      end;
      run;


data ha1;
set ha1;
array dxc[209] $ dxcode1-dxcode209;
array dx10c1[209] $dx10code1_1-dx10code1_209;
array dx10c2[209] $dx10code2_1-dx10code2_209;
array dx10c3[209] $dx10code3_1-dx10code3_209;
codea08=0;
do i=1 to 209;
      if dx10c1[i] in: ('A083' 'A084' 'A085')         then codea08=1;
      else if dx10c2[i] in: ('A083' 'A084' 'A085')    then codea08=1;
      else if dx10c3[i] in: ('A083' 'A084' 'A085')    then codea08=1;
      end;
      run;

proc freq data=ha1;
tables c_070 c_B15 c_A084 c_A09 c_B19 code009 code079 code787 codea08;
run;


data ha4;
set ha1;
array s[209] $ source1-source209;
hosp=0;
do i=1 to 209;
      if    s[i] in: ('DAD') then hosp=1;
      end;
      run;
```

```
data ha4;
set ha4;
array s[209] $ source1-source209;
ed=0;
do i=1 to 209;
      if    s[i] in: ('NACRS') then ed=1;
      end;
      run;

data ha4;
set ha4;
array s[209] $ source1-source209;
pc=0;
do i=1 to 209;
      if    s[i] in: ('OHIP') then pc=1;
      end;
      run;

data ha4;
set ha4;
array s[209] $ source1-source209;
sds=0;
do i=1 to 209;
      if    s[i] in: ('SDS') then sds=1;
      end;
      run;

proc freq data=ha4;
tables hosp ed pc sds;
run;


/*now merge with episodeperiod_hep to get datediffo and count summaries*/

data ha4a (keep=ikn);
set ha4;
run;

data epi_hep (keep=ikn datediffo count);
set episodeperiod_hep;
run;

proc sort data=epi_hep;
by ikn;
run;

data ha4_summary;
merge epi_hep (in=period) ha4a(in=hep);
by ikn;
if period and hep;
run;

proc means mean median min max data=ha4_summary;
var datediffo count;
run;
```

```
/* Now merge back with original cohort to describe hep A TP in more depth */

/*create data set from original cohort with 1 obs per patient based on first
health encounter*/
proc sort data=cohort;
by ikn first_admin_code_date;
run;

data uniqueikn;
set cohort;
if first.ikn then count=0;
count + 1;
if last.ikn then output;
by ikn;
run;

data ha3 (keep=ikn cohort_entry age_cohort_entry sex cic_immigrant cic_fsrce
admin_code_in_lookback);
set uniqueikn;
run;

data hep_dis;
merge ha3 (in=cohort) ha4_summary(in=hep);
by ikn;
if cohort and hep;
run;


/*merge together*/

data hep_dis2;
merge hep_dis (in=cohort) ha4(in=hep);
by ikn;
if cohort and hep;
run;


data MYDATA.final_hepa_fp;
set hep_dis2;
run; /*this is the final wide data set merged with key admin vars from the
cohort*/


/*for discriminant analysis*/

/*restrict to high sp*/
data final_hepa_fp;
set MYDATA.final_hepa_fp;
run;

data fake_hep (where=(h_sp=1));
set final_hepa_fp;
run;

proc freq data=fake_hep;
```

```
tables sex cic_immigrant admin_code_in_lookback;
run;

proc means median range data=fake_hep;
var age_cohort_entry datediffo;
run;

data fake_hep;
set fake_hep;
location=0;
if (hosp=1 or ed=1) then location=1;
run;

data fake_hep;
set fake_hep;
type="fake";
run;

/*pare down to vars I need*/
data fake_hep (keep=ikn age_cohort_entry sex cic_immigrant
admin_code_in_lookback datediffo location type);
set fake_hep;
run;


/*now create TP dataset for discriminant analysis*/


data cohort_tp (where=(iphis=1 and first_admin_code_date ne .));
set cohort;
run;

data cohort_tp;
set cohort_tp;
if     iphis_disease in ("HEPATITIS A") then diseasegp=1;
else if iphis_disease in ("MALARIA") then diseasegp=2;
else if iphis_disease in ("PARATYPHOID FEVER" "TYPHOID FEVER") then
diseasegp=3;
run;


/**********HEPA**********/


data cohort_tp_hep2;
set cohort_tp;
hep=0;
      if    dxcode    in: ('070' '009' '079' '136' '787') then hep=1;
      else if dx10code_1 in: ('B15' 'B19' 'A09' 'A083' 'A084' 'A085') then
hep=1;
      else if dx10code_2 in: ('B15' 'B19' 'A09' 'A083' 'A084' 'A085') then
hep=1;
      else if dx10code_3 in: ('B15' 'B19' 'A09' 'A083' 'A084' 'A085') then
hep=1;
      run;

proc freq data=cohort_tp_hep2;
```

```
tables hep;
run;

data cohort_tp_hep2 (where=(diseasegp=1 and hep=1));
set cohort_tp_hep2;
run;

/*Look at time between first and last date in individuals with >1 episode*/

data cohort_tp_hep2;
set cohort_tp_hep2;
datediffo=date-first_admin_code_date;
run;

proc sort data=cohort_tp_hep2;
by ikn first_admin_code_date date;
run;

data episodeperiod_hep2;
set cohort_tp_hep2;
if first.ikn then count=0;
count + 1;
if last.ikn then output;
by ikn;
run;


proc means mean median min max data=episodeperiod_hep2;
var datediffo count;
run;


/*Look at time between iPHIS episode date (onset) and health encounter dates
in admin data*/

data cohort_tp_hep2;
set cohort_tp_hep2;
datediffiphis=date - iphis_accurateepisodedate;
run;

/*based on looking at FN, -40 to 120 seems an appropriate range*/

data cohort_tp_hep2;
set cohort_tp_hep2;
if          datediffiphis < -40 then iphisdategp2=1;
else if datediffiphis ge -40 and datediffiphis le 120 then iphisdategp2=2;
else if datediffiphis > 120   then iphisdategp2=3;
run;


/*Apply rule:  IF codes 787 or 079 or 009 or A099 AND iphisdategp2 = 1 or 3,
exclude*/

data cohort_tp_hep3;
set cohort_tp_hep2;
fn=0;
if    dxcode in: ('787' '079' '009') and (iphisdategp2 ne 2)      then fn=1;
```

```
else if dx10code_1 in: ('A099') and (iphisdategp2 ne 2)          then fn=1;
run;

proc freq data=cohort_tp_hep3;
tables fn;
run;

data cohort_tp_hep4 (where=(fn ne 1));
set cohort_tp_hep3;
run;


/** BY PATIENT ANALYSIS **/

data cohort_tp_hep4 (rename=(dx10code_1=dx10code1_ dx10code_2=dx10code2_
dx10code_3=dx10code3_));
set cohort_tp_hep4;
run;

%MAKEWIDE(data          = cohort_tp_hep4,
          out           = source_wide,
          id            = ikn,
          var           = source);

data source_wide;
set source_wide;
run;

%MAKEWIDE(data          = cohort_tp_hep4,
          out           = date_wide,
          id            = ikn,
          var           = date);

data date_wide;
set date_wide;
run;

%MAKEWIDE(data          = cohort_tp_hep4,
          out           = dxcode_wide,
          id            = ikn,
          var           = dxcode);

data dxcode_wide;
set dxcode_wide;
run;

%MAKEWIDE(data          = cohort_tp_hep4,
          out           = dx10code1_wide,
          id            = ikn,
          var           = dx10code1_);

data dx10code1_wide;
set dx10code1_wide;
run;

%MAKEWIDE(data          = cohort_tp_hep4,
          out           = dx10code2_wide,
```

```
            id          = ikn,
            var         = dx10code2_);

data dx10code2_wide;
set dx10code2_wide;
run;

%MAKEWIDE(data          = cohort_tp_hep4,
            out         = dx10code3_wide,
            id          = ikn,
            var         = dx10code3_);

data dx10code3_wide;
set dx10code3_wide;
run;


/*now merge together*/
data hepa_wide2;
merge source_wide
date_wide
dxcode_wide
dx10code1_wide
dx10code2_wide
dx10code3_wide;
by ikn;
run;


/*Look at constellation*/

/*high*/
data ha1;
set hepa_wide2;
array dxc[15] $ dxcode1-dxcode15;
array dx10c1[15] $dx10code1_1-dx10code1_15;
array dx10c2[15] $dx10code2_1-dx10code2_15;
array dx10c3[15] $dx10code3_1-dx10code3_15;
h_sp=0;
do i=1 to 15;
      if          dxc[i] in:('070')             then h_sp=1;
      else if dx10c1[i] in: ('B15')       then h_sp=1;
      else if dx10c2[i] in: ('B15')       then h_sp=1;
      else if dx10c3[i] in: ('B15')       then h_sp=1;
      end;
      run;

proc freq data=ha1;
tables h_sp;
run;


/*moderate*/
data ha1;
set ha1;
array dxc[15] $ dxcode1-dxcode15;
array dx10c1[15] $dx10code1_1-dx10code1_15;
```

```
array dx10c2[15] $dx10code2_1-dx10code2_15;
array dx10c3[15] $dx10code3_1-dx10code3_15;
m_sp=0;
do i=1 to 15;
      if          dxc[i] in:('009')             then m_sp=1;
      else if dx10c1[i] in: ('B19')       then m_sp=1;
      else if dx10c2[i] in: ('B19')       then m_sp=1;
      else if dx10c3[i] in: ('B19')       then m_sp=1;
      end;
      run;

proc freq data=ha1;
tables m_sp;
run;


/*low*/
data ha1;
set ha1;
array dxc[15] $ dxcode1-dxcode15;
array dx10c1[15] $dx10code1_1-dx10code1_15;
array dx10c2[15] $dx10code2_1-dx10code2_15;
array dx10c3[15] $dx10code3_1-dx10code3_15;
l_sp=0;
do i=1 to 15;
      if          dxc[i]            in: ('079' '136' '787')
      then l_sp=1;
      else if dx10c1[i]       in: ('A09' 'A083' 'A084' 'A085')    then
l_sp=1;
      else if dx10c2[i]       in: ('A09' 'A083' 'A084' 'A085')    then
l_sp=1;
      else if dx10c3[i]       in: ('A09' 'A083' 'A084' 'A085')    then
l_sp=1;
      end;
      run;

proc freq data=ha1;
tables l_sp;
run;


/*Describe codes and sources*/

data ha4;
set ha1;
array dxc[15] $ dxcode1-dxcode15;
array dx10c1[15] $dx10code1_1-dx10code1_15;
array dx10c2[15] $dx10code2_1-dx10code2_15;
array dx10c3[15] $dx10code3_1-dx10code3_15;
code070=0;
do i=1 to 15;
      if          dxc[i] in:('070')       then code070=1;
      end;
      run;

data ha4;
set ha4;
```

```
array dxc[15] $ dxcode1-dxcode15;
array dx10c1[15] $dx10code1_1-dx10code1_15;
array dx10c2[15] $dx10code2_1-dx10code2_15;
array dx10c3[15] $dx10code3_1-dx10code3_15;
codeb15=0;
do i=1 to 15;
      if dx10c1[i] in: ('B15')           then codeb15=1;
      else if dx10c2[i] in: ('B15')      then codeb15=1;
      else if dx10c3[i] in: ('B15')      then codeb15=1;
      end;
      run;

proc freq data=ha4;
tables code070 codeb15;
run;

data ha4;
set ha4;
array dxc[15] $ dxcode1-dxcode15;
array dx10c1[15] $dx10code1_1-dx10code1_15;
array dx10c2[15] $dx10code2_1-dx10code2_15;
array dx10c3[15] $dx10code3_1-dx10code3_15;
code009=0;
do i=1 to 15;
      if          dxc[i] in:('009')             then code009=1;
      end;
      run;

data ha4;
set ha4;
array dxc[15] $ dxcode1-dxcode15;
array dx10c1[15] $dx10code1_1-dx10code1_15;
array dx10c2[15] $dx10code2_1-dx10code2_15;
array dx10c3[15] $dx10code3_1-dx10code3_15;
code079=0;
do i=1 to 15;
      if          dxc[i] in:('079')             then code079=1;
      end;
      run;

data ha4;
set ha4;
array dxc[15] $ dxcode1-dxcode15;
array dx10c1[15] $dx10code1_1-dx10code1_15;
array dx10c2[15] $dx10code2_1-dx10code2_15;
array dx10c3[15] $dx10code3_1-dx10code3_15;
code787=0;
do i=1 to 15;
      if          dxc[i] in:('787')             then code787=1;
      end;
      run;

data ha4;
set ha4;
array dxc[15] $ dxcode1-dxcode15;
array dx10c1[15] $dx10code1_1-dx10code1_15;
array dx10c2[15] $dx10code2_1-dx10code2_15;
```

```
array dx10c3[15] $dx10code3_1-dx10code3_15;
codeb19=0;
do i=1 to 15;
      if dx10c1[i] in: ('B19')           then codeb19=1;
      else if dx10c2[i] in: ('B19')      then codeb19=1;
      else if dx10c3[i] in: ('B19')      then codeb19=1;
      end;
      run;

data ha4;
set ha4;
array dxc[15] $ dxcode1-dxcode15;
array dx10c1[15] $dx10code1_1-dx10code1_15;
array dx10c2[15] $dx10code2_1-dx10code2_15;
array dx10c3[15] $dx10code3_1-dx10code3_15;
codea09=0;
do i=1 to 15;
      if dx10c1[i] in: ('A09')           then codea09=1;
      else if dx10c2[i] in: ('A09')      then codea09=1;
      else if dx10c3[i] in: ('A09')      then codea09=1;
      end;
      run;

data ha4;
set ha4;
array dxc[15] $ dxcode1-dxcode15;
array dx10c1[15] $dx10code1_1-dx10code1_15;
array dx10c2[15] $dx10code2_1-dx10code2_15;
array dx10c3[15] $dx10code3_1-dx10code3_15;
codea08=0;
do i=1 to 15;
      if dx10c1[i] in: ('A083' 'A084' 'A085')         then codea08=1;
      else if dx10c2[i] in: ('A083' 'A084' 'A085')    then codea08=1;
      else if dx10c3[i] in: ('A083' 'A084' 'A085')    then codea08=1;
      end;
      run;

proc freq data=ha4;
tables code009 code079 code787 codeb19 codea09 codea08;
run;



data ha4;
set ha4;
array s[15] $ source1-source15;
hosp=0;
do i=1 to 15;
      if    s[i] in: ('DAD') then hosp=1;
      end;
      run;

data ha4;
set ha4;
array s[15] $ source1-source15;
ed=0;
do i=1 to 15;
```

```
      if    s[i] in: ('NACRS') then ed=1;
      end;
      run;

data ha4;
set ha4;
array s[15] $ source1-source15;
pc=0;
do i=1 to 15;
      if    s[i] in: ('OHIP') then pc=1;
      end;
      run;

data ha4;
set ha4;
array s[15] $ source1-source15;
sds=0;
do i=1 to 15;
      if    s[i] in: ('SDS') then sds=1;
      end;
      run;

proc freq data=ha4;
tables hosp ed pc sds;
run;


/*now merge back with original cohort to describe all TP*/

/*create data set from original cohort with 1 obs per patient based on first
health encounter*/
proc sort data=cohort;
by ikn first_admin_code_date;
run;

data uniqueikn;
set cohort;
if first.ikn then count=0;
count + 1;
if last.ikn then output;
by ikn;
run;

data h (keep=ikn cohort_entry age_cohort_entry sex cic_immigrant cic_fsrce
admin_code_in_lookback);
set uniqueikn;
run;

data h2;
merge h (in=cohort) ha4 (in=hep);
by ikn;
if cohort and hep;
run;


/*now merge with episodeperiod_hep to get datediffo and count summaries*/
```

```
data ha4a (keep=ikn);
set ha4;
run;

data epi_hep (keep=ikn datediffo count);
set episodeperiod_hep2;
run;

proc sort data=epi_hep;
by ikn;
run;

data ha4_summary;
merge epi_hep (in=period) ha4a(in=hep);
by ikn;
if period and hep;
run;

proc means mean median min max data=ha4_summary;
var datediffo count;
run;


data hep_dis_tp;
merge h2 (in=cohort) ha4_summary (in=hep);
by ikn;
if cohort and hep;
run;


data MYDATA.final_hepa_tp;
set hep_dis_tp;
run; /*this is the final wide data set merged with key admin vars from the
cohort*/


/*for discriminant analysis*/

/*restrict to high sp*/
data final_hepa_tp;
set MYDATA.final_hepa_tp;
run;

data real_hep (where=(h_sp=1));
set final_hepa_tp;
run;

proc freq data=real_hep;
tables sex cic_immigrant admin_code_in_lookback;
run;

proc means mean median min max data=real_hep;
var age_cohort_entry datediffo;
run;

data real_hep;
set real_hep;
```

```
location=0;
if (hosp=1 or ed=1) then location=1;
run;

data real_hep;
set real_hep;
type="real";
run;

/*pare down to vars I need*/
data real_hep (keep=ikn age_cohort_entry sex cic_immigrant
admin_code_in_lookback datediffo location);
set real_hep;
run;



/*now for discriminant analysis*/

data combine_hep;
set fake_hep real_hep;
run;

data combine_hep;
set combine_hep;
sex2=0;
if sex='M' then sex2=0;
else if sex='F' then sex2=1;
run;


proc discrim data=combine_hep pool=test crossvalidate;
class type;
var age_cohort_entry sex2 cic_immigrant admin_code_in_lookback datediffo
location;
priors "real"=0.0027 "fake"=0.9973;
run;


data MYDATA.combine_hep;
set combine_hep;
run;



/*Capture Recapture Numbers - July 6 2017*/

data cohort;
set MYDATA.admin_codes_iphis_02may2017;
run;

/*select all hosp with B15 for hep A*/

data capture;
set cohort;
if (dx10code_1 in: ('B15') or dx10code_2 in: ('B15') or dx10code_3 in:
('B15')) and source='DAD' then hepahosp=1;
```

```
else if hepahosp=. then hepahosp=0;
run;


proc freq data=capture;
tables hepahosp;
run;

data capture_hep (where=(hepahosp=1));
set capture;
run;

proc freq data=capture_hep;
tables iphis;
run;


/*how many unique individuals?*/

proc sort data=capture_hep;
by ikn first_admin_code_date;
run;

data uniqueikn_hep;
set capture_hep;
if first.ikn then count=0;
count + 1;
if last.ikn then output;
by ikn;
run;

proc freq data=uniqueikn_hep;
tables iphis;
run;

/*for TP, select only those with HEPATITIS A disease in iPHIS*/
proc freq data=uniqueikn_hep;
tables iphis*iphis_disease;
run;


/*select all hosp with B5 for malaria*/

data capture;
set capture;
if (dx10code_1 in: ('B5') or dx10code_2 in: ('B5') or dx10code_3 in: ('B5'))
and source='DAD' then malhosp=1;
else if malhosp=. then malhosp=0;
run;

proc freq data=capture;
tables malhosp;
run;

data capture_mal (where=(malhosp=1));
set capture;
run;
```

```
proc freq data=capture_mal;
tables iphis;
run;


/*how many unique individuals?*/

proc sort data=capture_mal;
by ikn first_admin_code_date;
run;

data uniqueikn_mal;
set capture_mal;
if first.ikn then count=0;
count + 1;
if last.ikn then output;
by ikn;
run;

proc freq data=uniqueikn_mal;
tables iphis;
run;

/*for TP, select only those with MALARIA disease in iPHIS*/
proc freq data=uniqueikn_mal;
tables iphis*iphis_disease;
run;


/*select all hosp with A01 for T/PT*/

data capture;
set capture;
if (dx10code_1 in: ('A01') or dx10code_2 in: ('A01') or dx10code_3 in:
('A01')) and source='DAD' then tpthosp=1;
else if tpthosp=. then tpthosp=0;
run;

proc freq data=capture;
tables tpthosp;
run;

data capture_tpt (where=(tpthosp=1));
set capture;
run;

proc freq data=capture_tpt;
tables iphis;
run;

/*how many unique individuals?*/

proc sort data=capture_tpt;
by ikn first_admin_code_date;
run;
```

```
data uniqueikn_tpt;
set capture_tpt;
if first.ikn then count=0;
count + 1;
if last.ikn then output;
by ikn;
run;

proc freq data=uniqueikn_tpt;
tables iphis;
run;

/*for TP, select only those with MALARIA disease in iPHIS*/
proc freq data=uniqueikn_tpt;
tables iphis*iphis_disease;
run;



/*check to see if 87 hosp but not all have A01*/

data check;
set cohort;
tpt=0;
      if dxcode in: ('002' '009' '003' '005' '136' '784' '787') then tpt=1;
      else if dx10code_1 in: ('A01' 'A029' 'A021' 'A049' 'A059' 'A09' 'A499')
then tpt=1;
      else if dx10code_2 in: ('A01' 'A029' 'A021' 'A049' 'A059' 'A09' 'A499')
then tpt=1;
      else if dx10code_3 in: ('A01' 'A029' 'A021' 'A049' 'A059' 'A09' 'A499')
then tpt=1;
      run;

proc freq data=check;
tables tpt;
run;

data check (where=((iphis_disease='TYPHOID FEVER' or
iphis_disease='PARATYPHOID FEVER') and tpt=1));
set check;
run;


/*need to correct for two cases with two distinct disease episodes*/

data duplicates;
set check;
dup=0;
if ikn=X or ikn=X then dup=1;
run;

data duplicates (where=(dup=1));
set duplicates;
run;

data duplicates (drop=iphis_accurateepisodedate iphis_disease
iphis_countryofbirth iphis_travelassociated iphis_traveldestination1);
```

```
set duplicates;
run;

data duplicates;
set duplicates;
length rec $20;
rec='rec2';
run;

data duplicates;
set duplicates;
length ikn2 $20;
ikn2=catx("_", of ikn rec);
run;

data duplicates (drop=ikn dup _a rec);
set duplicates;
run;

data duplicates;
rename ikn2=ikn iphis_accurateepisodedate_rec2=iphis_accurateepisodedate
iphis_disease_rec2=iphis_disease
iphis_countryofbirth_rec2=iphis_countryofbirth
iphis_travelassociated_rec2=iphis_travelassociated
iphis_traveldestination1_rec2=iphis_traveldestination1;
set duplicates;
run;

data duplicates;
set duplicates;
run;

/*now append to cohort of TP*/

data cohort_tp_tpt3 (drop=iphis_accurateepisodedate_rec2 iphis_disease_rec2
iphis_countryofbirth_rec2 iphis_travelassociated_rec2
iphis_traveldestination1_rec2);
set check;
run;

data cohort_tp_tpt3;
set cohort_tp_tpt3;
length ikn2 $20;
ikn2=ikn;
run;

data cohort_tp_tpt3 (drop=ikn);
set cohort_tp_tpt3;
run;

data cohort_tp_tpt3 (rename=ikn2=ikn);
set cohort_tp_tpt3;
run;

proc append base=cohort_tp_tpt3 data=duplicates;
run;
```

```
data cohort_tp_tpt_append;
set cohort_tp_tpt3;
run;


data cohort_tp_tpt_append;
retain ikn iphis iphis_accurateepisodedate iphis_disease iphis_countryofbirth
iphis_travelassociated iphis_traveldestination1
first_admin_code_date cohort_entry age_cohort_entry sex cic_immigrant
cic_fsrce   admin_code_in_lookback elig_at_iphis elig_1yr
elig_2yr elig_3yr source date ddate dxcode dx10code_1 dxtype_1 dxpref_1
      dx10code_2 dxtype_2     dxpref_2 dx10code_3     dxtype_3 dxpref_3
peel_region age   diseasegp tpt;
set cohort_tp_tpt3;
run;

data cohort_tp_tpt_append;
set cohort_tp_tpt_append;
run;

/*how many unique individuals?*/

proc sort data=cohort_tp_tpt_append;
by ikn first_admin_code_date;
run;

data uniqueikn_tpt;
set cohort_tp_tpt_append;
if first.ikn then count=0;
count + 1;
if last.ikn then output;
by ikn;
run;


data cohort_tp_tpt_append;
set cohort_tp_tpt_append;
run;

data cohort_tp_tpt_append;
set cohort_tp_tpt_append;
if source='DAD' then hosp=1;
else if hosp=. then hosp=0;
run;

data hosp (where=(hosp=1));
set cohort_tp_tpt_append;
run;

proc sort data=hosp;
by ikn first_admin_code_date;
run;

data uniqueikn_tpt_hosp;
set hosp;
if first.ikn then count=0;
count + 1;
```

```
if last.ikn then output;
by ikn;
run;

/*now narrow to only A01*/

data c;
set uniqueikn_tpt_hosp;
if dx10code_1 in: ('A01') or dx10code_2 in: ('A01') or dx10code_3 in: ('A01')
then tpthosp=1;
else if tpthosp=. then tpthosp=0;
run;

proc freq data=c;
tables tpthosp;
run;


/*overall, how many FP were hosp with B15, B5 or A01?*/

proc freq data=capture;
tables iphis*hepahosp iphis*malhosp iphis*tpthosp;
run;

data capture;
set capture;
if iphis=0 and (hepahosp=1 or malhosp=1 or tpthosp=1) then fphosp=1;
else if fphosp=. then fphosp=0;
run;

proc freq data=capture;
tables fphosp;
run;
```
