# Supplementary material for: Accuracy of health administrative data to identify cases of reportable travel or migration-related infectious diseases in Ontario, Canada
Source: PLoS One. 2018 Nov 7;13(11):e0207030. doi: 10.1371/journal.pone.0207030 (PMC6221317; doi:10.1371/journal.pone.0207030)

Supporting Information File 5: Sensitivity Analyses

Fig 1. The absolute change in a) sensitivity and b) positive predictive value estimates following four sensitivity analyses: 1 - excluding individuals with a questionable diagnosis prefix in hospitalization (DAD) or emergency department (NACRS) data (which indicates uncertainty of diagnosis), 2 - excluding individuals with a generic immunization billing code (G538) 0-60 days prior to diagnosis in outpatient physician data (OHIP), 3 - excluding individuals with a generic immunization billing code (G538) on the same date as diagnosis in OHIP, and 4 - excluding individuals with a 070 diagnostic code during both the study and lookback period of 3-24 months prior in OHIP.

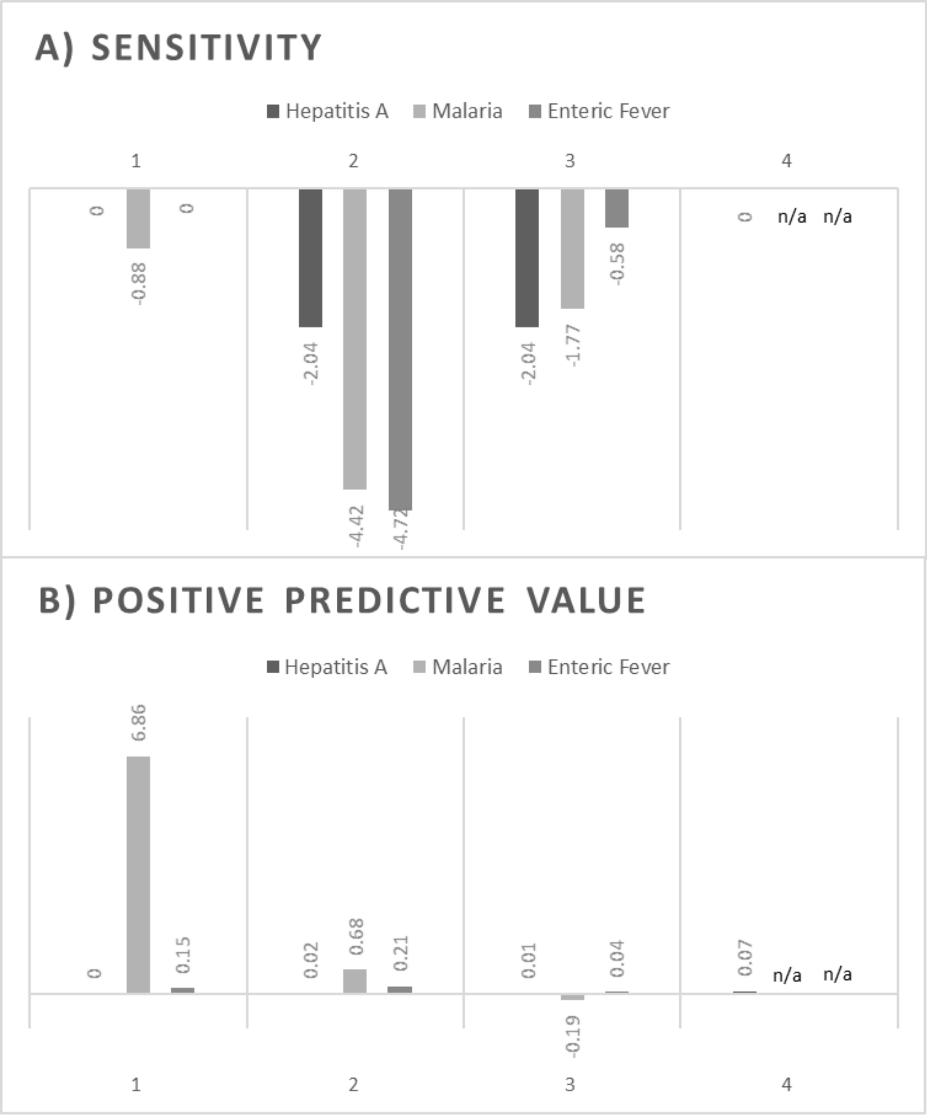

Supplement: S5 File — (PDF) [file pone.0207030.s005.pdf]
